# β-Turn Induction by a Diastereopure Azepane-Derived Quaternary Amino Acid

**DOI:** 10.1021/acs.joc.3c01689

**Published:** 2023-09-29

**Authors:** Diego Núñez-Villanueva, Adrián Plata-Ruiz, Ignacio Romero-Muñiz, Ignacio Martín-Pérez, Lourdes Infantes, Rosario González-Muñiz, Mercedes Martín-Martínez

**Affiliations:** † Instituto de Química Médica (IQM-CSIC), Juan de la Cierva 3, 28006 Madrid, Spain; ‡ Universidad Autónoma de Madrid, Química Orgánica, Francisco Tomás y Valiente, 7, 28049 Madrid, Spain; § Instituto de Química Física Rocasolano (IQFR-CSIC), Serrano 119, 28006 Madrid, Spain

## Abstract

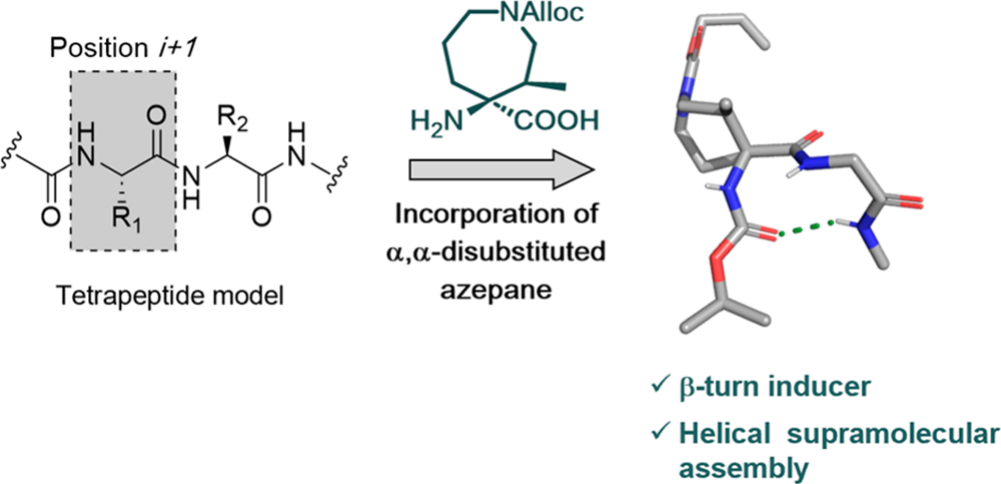

β-Turns are one of the most common secondary structures found in proteins. In the interest of developing novel β-turn inducers, a diastereopure azepane-derived quaternary amino acid has been incorporated into a library of simplified tetrapeptide models in order to assess the effect of the azepane position and peptide sequence on the stabilization of β-turns. The conformational analysis of these peptides by molecular modeling, NMR spectroscopy, and X-ray crystallography showed that this azepane amino acid is an effective β-turn inducer when incorporated at the *i* + 1 position. Moreover, the analysis of the supramolecular self-assembly of one of the β-turn-containing peptide models in the solid state reveals that it forms a supramolecular helical arrangement while maintaining the β-turn structure. The results here presented provide the basis for the use of this azepane quaternary amino acid as a strong β-turn inducer in the search for novel peptide-based bioactive molecules, catalysts, and biomaterials.

## Introduction

**Figure 1:**
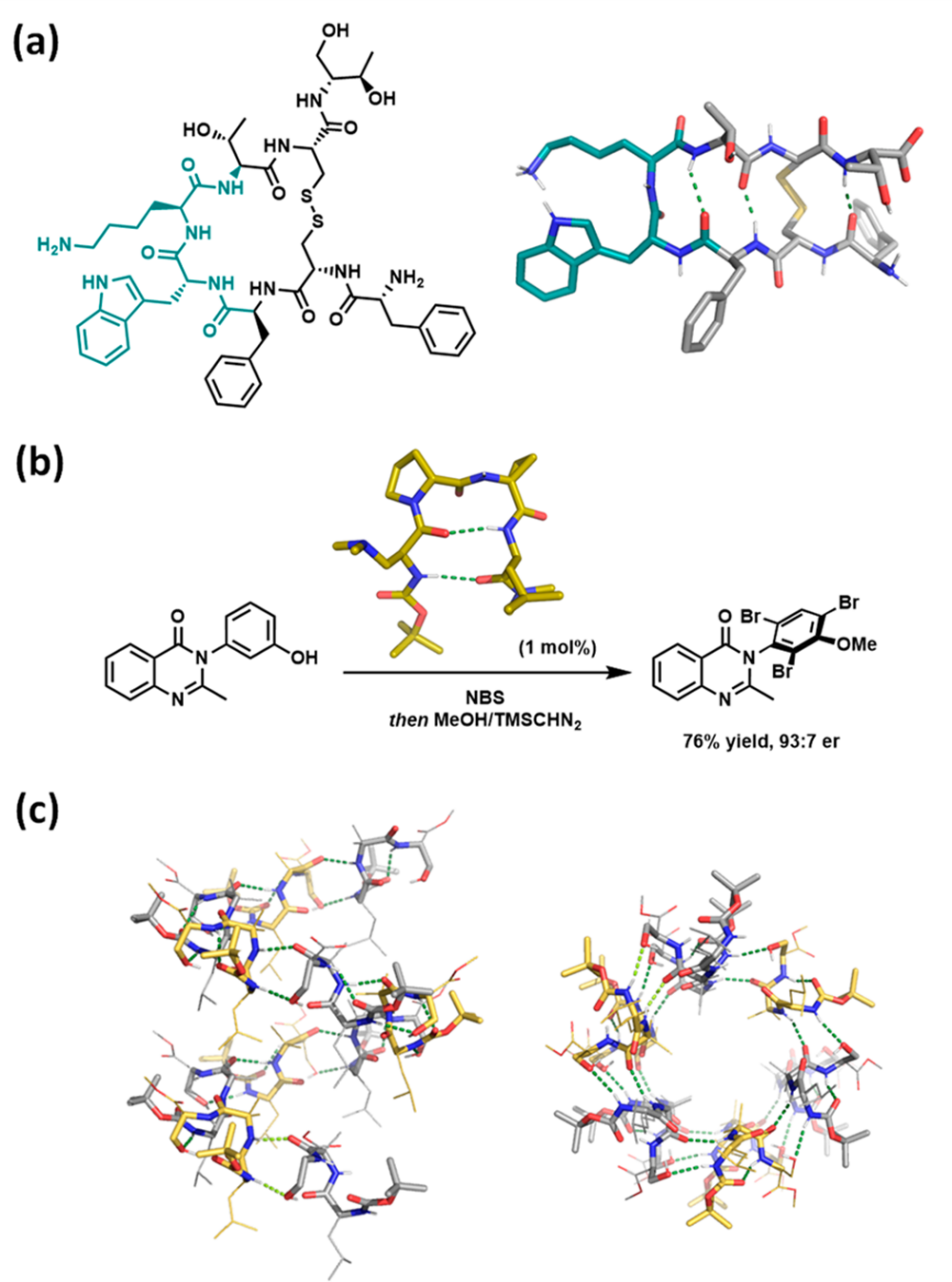
(a) Chemical and X-ray structures of octreotide (PDB 6VC1).^22^ (b) Atroposelective bromination of 3-arylquinazolin-4(3*H*)-ones catalyzed by a β-turn-containing tetrapeptide (X-ray structure shown, CCDC 1412920). Adapted with permission from ref (15). Copyright 2015 American Chemical Society. (c) Two views of the X-ray structure of the tetrapeptide model Boc-Leu-Aib-Ser-OMe showing an intermolecular H-bonded supramolecular helical column (CCDC 1434362). Adapted from ref (19) with permission from the Royal Society of Chemistry. 3D structure images were created with PyMOL.^23^

Protein–protein and peptide–protein interactions mediate essential cellular processes, including antigen–antibody interactions, cell signaling networks, or programmed cell death.^1^ Misregulation of these interactions is often implicated in disease states, so they constitute promising therapeutic targets via either their inhibition or stabilization.^2^ Despite the large and often flat contact surfaces implicated in these interactions, a few key residues with a defined secondary structure often contribute to the majority of the binding affinity.^3,4^ These interactions can therefore be modulated by small-molecule mimetics or stabilizers of such secondary structure elements, as the conformation and binding capabilities of the native sequences are not retained when they lack the structural reinforcement provided by the protein environment.^5^ Among common elements of secondary structure found in proteins, the β-turn accounts for more than 20% of protein residues.^6,7^ It reverses the peptide chain direction, facilitating the formation of globular structures.^8^ In addition to its key role in protein folding and the stabilization of tertiary structures, β-turns are often implicated in molecular recognition.^9^ For example, octreotide is a synthetic octapeptide mimicking natural somatostatin and is used for the treatment of carcinoid syndrome and neuroendocrine tumors (Figure 1a).^10,11^ It possesses enhanced biological affinity and metabolic stability due to the β-turn stabilization provided by substitution of l-Trp by d-Trp.^12^ Another relevant example is the type II′ β-turn arrangement adopted by the cyclic pentapeptide c(RGDfV), which is essential for the inhibition of integrins implicated in angiogenesis and metastasis.^13^ A cyclic, conformationally restricted analogue of α-melanocyte-stimulating hormone (α-MSH) displaying a β-turn conformation is four orders of magnitude more active than the native hormone.^14^ Interestingly, the β-turn structure was found to be crucial for other applications apart from medicinal chemistry, such as peptide-based catalysis and self-assembly. Figure 1b shows an example of a β-turn-containing short peptide able to catalyze the atroposelective bromination of pharmaceutically relevant quinazolinones with high levels of enantioinduction.^15,16^ In addition, β-turns have been exploited in the search of novel peptide-based materials.^17−21^ As an example, a tetrapeptide model containing Leu, Aib (α-aminoisobutyric acid), and Ser adopts a type-III′ β-turn, which self-assembles to form a supramolecular nanotube in the solid state (Figure 1c).^19^

The role of β-turns in the regulation of key biological processes and as useful scaffolds in peptide-based catalysts and biomaterials has prompted the development of strategies to mimic or stabilize such peptide secondary structure over the last decades. A classic approach toward β-turn mimetics has been the substitution of the characteristic H-bond between residues *i* and *i* + 3 for a covalent bond, as well as the use of rigid synthetic scaffolds able to display the side chains in the appropriate geometry.^24−35^ Nevertheless, the most common strategy for the stabilization of β-turns is the incorporation of constrained amino acids into short peptide sequences.^36,37^ Of particular interest is the incorporation of C^α,α^-tetrasubstituted amino acids in peptide sequences, which is able to bias the conformational preferences toward helical and turn structures.^38−40^ Cyclic quaternary amino acids provide greater conformational restriction compared with linear analogs, as well as showing enhanced metabolic stability.^38,41−43^ In this sense, studies on cycloaliphatic and heterocyclic tetrasubstituted amino acids have demonstrated their ability to induce β-turns or helical conformations in short peptides.^38,43−52^ However, in most cases, the constrained residue is used in combination with other restricted amino acids such as proline or Aib.

**Scheme 1:**
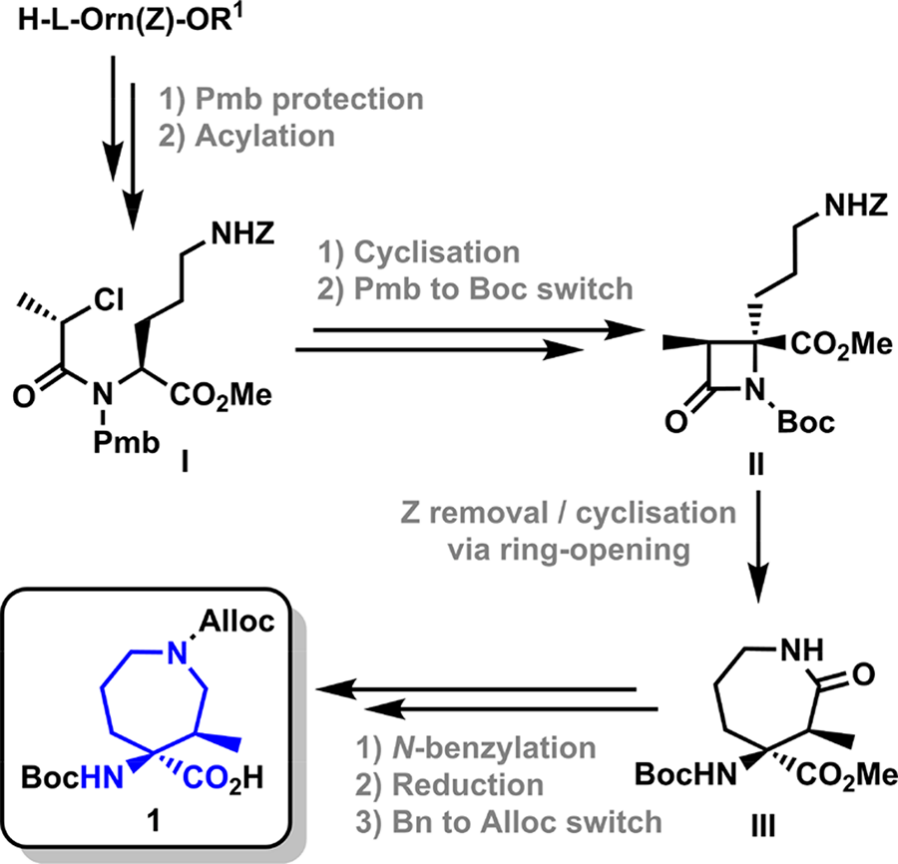
Reported Stereoselective Synthesis of α,α-Disubstituted Azepane Amino Acid **1**(53)

We have previously described the stereoselective synthesis of a quaternary azepane amino acid from commercial ornithine derivatives (Scheme 1).^53^ The key intermediate is the diastereopure β-lactam **II** synthesized via stereoselective intramolecular cyclization of the corresponding chloropropionyl derivative **I**. The spontaneous intramolecular β-lactam ring opening reaction, after deprotection of the ornithine side chain, leads to the formation of the 2-oxoazepane amino acid **III**. Selective reduction of the 2-oxoazepane ring yields diastereomerically pure (3*R*,4*S*)-4-amino-4-carboxy-3-methylazepane (Aze) **1**. This quaternary amino acid displays a set of properties that make it especially suited for its application as peptide secondary structure inducer. There are not many studies on the ability of seven-membered quaternary amino acids to constrain peptide conformations on their own, i.e., without the synergistic effect of additional restricted residues.^48,49,54,55^ The azepane heterocycle in **1** also enables the incorporation of structural diversity and functionality in the constrained peptides through the Alloc-protected nitrogen, for example, fluorescent dyes or affinity labels for visualization and immobilization. In addition, it contains two chiral centers, which can be easily defined in the synthesis step opening the possibility of studying the effect of chirality in the induction of secondary structure elements. In fact, we have reported the synthesis of the four possible diastereoisomers of the 2-oxoazepane derivative **III**.^53^ In a previous report, we have also demonstrated that the azepane **1** is an effective inducer of 3_10_ helices in short alanine-based pentapeptide models.^56^ In this article, we report the induction of single β-turns by this quaternary amino acid in the shortest possible peptide model. We present a study of the key factors governing the folding of the peptide chain into a β-turn, such as the position of the constrained amino acid in the sequence and the compatibility with proteinogenic amino acids in the remaining central position of the turn.

## Results and Discussion

The conformational preferences of tetrapeptide models incorporating the azepane amino acid **1** were first analyzed using molecular dynamics (MD) simulations with the Amber 10 suite of programs.^57^ Simplified tetrapeptide systems were used in which the *N*-terminal amino acid (*i*) was replaced by an acetyl group and the *C*-terminal (*i* + 3) by a *N*-methylamide. The β-turn induction was evaluated through its characteristic topographic parameters, i.e., H-bond distance CO^*i*^–NH^*i*+3^ and the backbone dihedral angles of the central residues. We first studied the influence of the position of the constrained amino acid within the central residues of alanine-based tetrapeptide models. As a reference, it is worth mentioning that the conformational properties of alanine-based short peptides have been analyzed in depth both theoretically and experimentally, showing a strong tendency for extended conformations.^58−60^ When the azepane residue was incorporated at the *i* + 1 position, molecular modeling results showed that the 100% of the conformers within a 3 kcal·mol^–1^ window from the global minimum stabilize type I or III β-turns. At the *i* + 2 position, the global minimum also displays a β-turn, but there is another family of conformers within a 3 kcal·mol^–1^ window in which the characteristic β-turn H-bond is not present (see the Supporting Information for details). Thus, MD studies suggest that the incorporation of **1** at the *i* + 1 position is preferred over that at the *i* + 2 position, with a higher degree of β-turn induction. This is in agreement with the preferred position for other constrained amino acids, as the restriction in the dihedral angles imposed by these residues at such position facilitates the β-turn nucleation.^61,62^

With the azepane fixed at the *i* + 1 position, we next evaluated the effect of the amino acid side chain at position *i* + 2, again using MD simulations. Calculations were performed in tetrapeptide models incorporating the azepane residue at the *i* + 1 position and the 20 proteinogenic amino acids at the *i* + 2 position. MD studies suggested that the azepane derivative is a strong β-turn inducer, except when it is combined with Gln, Arg, and Asp at the *i* + 2 position. In these cases, less than 50% of the conformers within a 3 kcal·mol^–1^ window from the global minimum display a β-turn structure. The presence of polar residues tends to destabilize the β-turn due to competing H-bond formation between the side chain and the peptide backbone (see the Supporting Information for details). Taking these results into account, we selected a representative set of amino acids to prepare a library of Boc-Aze(Alloc)-Xaa-NHMe tetrapeptide models, where Xaa was chosen to be alanine, valine, leucine, or phenylalanine (residues with a high tendency to adopt β-turns) as well as serine, lysine, or glycine (with a medium tendency toward β-turns). This small library covers from hydrophobic aliphatic and aromatic residues to polar ones, displaying different side chain branching patterns. Additionally, we also prepared the corresponding alanine-based tetrapeptide model Boc-Ala-Aze(Alloc)-NHMe to experimentally assess the influence of the azepane position in the reverse turn induction.

**Scheme 2:**
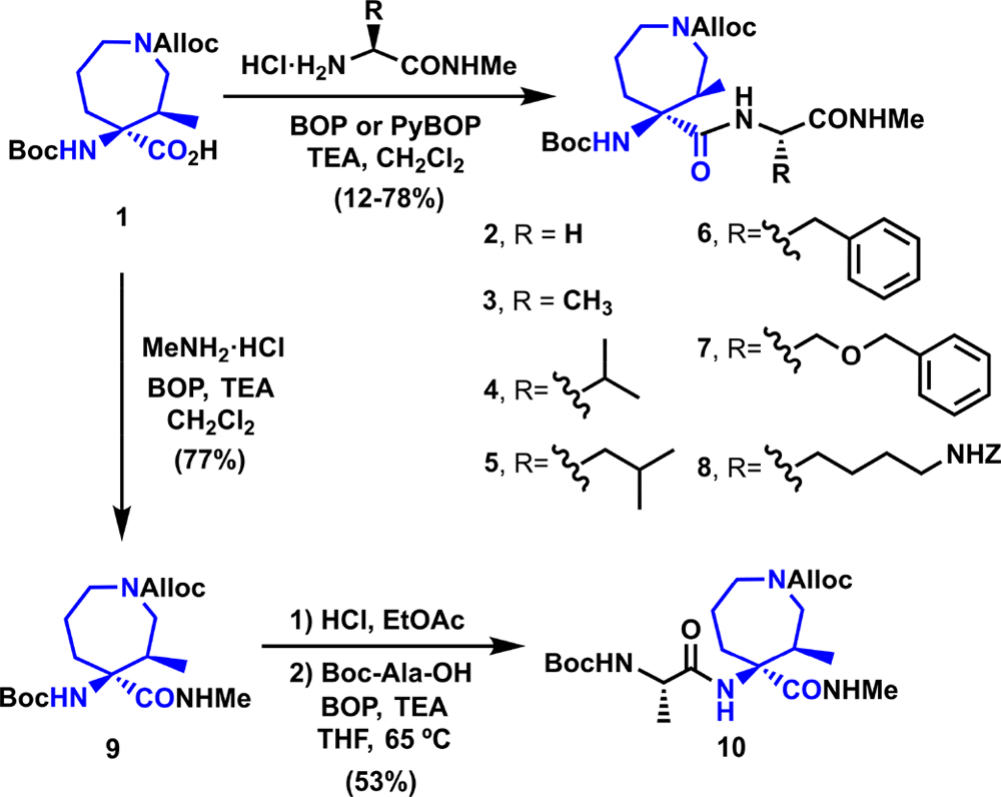
Synthesis of Tetrapeptide Models **2**–**8** and **10**

Scheme 2 shows the synthetic pathway for the preparation of the designed small peptide library via classical solution peptide synthesis. Compounds **2**-**8** were obtained through peptide coupling between the azepane residue **1**(56) and H-Xaa-NHMe, with Xaa being Gly, Ala, Val, Leu, Phe, Ser(Bn) and Lys(Z). Alternatively, peptide coupling between azepane **1** and MeNH_2_ yielded derivative **9** in a good yield. The removal of the Boc group followed by amide coupling with Boc-Ala-OH provided dipeptide **10**.

**Figure 2:**
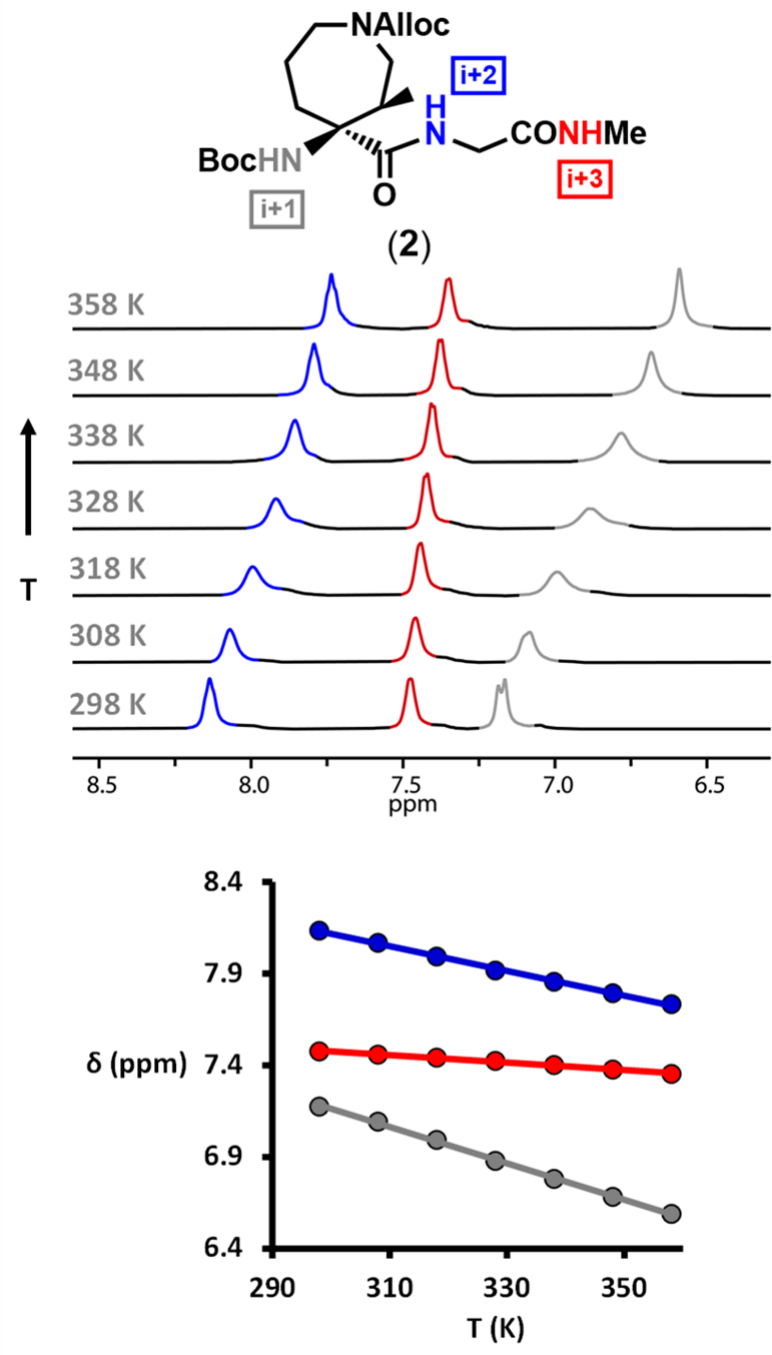
Amide proton region of the ^1^H NMR spectra at different temperatures for Boc-Aze(Alloc)-Gly-NHMe **2** (DMSO-*d*_6_, 400 MHz) and plot of Δδ against Δ*T* for all the NH protons (NH^*i*+1^ in gray, NH^*i*+2^ in blue, and NH^*i*+3^ in red). The slopes of the fitted lines correspond to the temperature coefficient for each NH and are listed in Table 1.

The conformational preferences in solution of tetrapeptide models **2**–**8** and **10** were evaluated by variable-temperature NMR experiments. β-Turns are usually stabilized by an intramolecular H-bond between the carbonyl oxygen of residue *i* and the NH proton of residue *i* + 3. The existence of this H-bond in compounds **2**–**8** and **10** was assessed by the variation of the amide proton chemical shift with temperature. In DMSO-*d*_6_, it has been established that the values of temperature coefficients (Δδ/Δ*T*) for amide protons equal to or lower than 3 ppb·K^–1^ (in absolute value) are indicative of such NHs participating in an intramolecular H-bond.^63,64^ In contrast, solvent-exposed amide protons typically display values over 4 ppb·K^–1^, while values within 3–4 ppb·K^–1^ are not conclusive. Figure 2 shows the amide proton region of the ^1^H NMR spectra for compound **2** at different temperatures, as well as the plot of the chemical shift variation with temperature (Δδ/Δ*T*). The observed linear correlation was used to calculate the temperature coefficient for all the amide protons. For compound **2**, only NH^*i*+3^ has a temperature coefficient compatible with the presence of an intramolecular H-bond, suggesting the adoption of a β-turn in solution. It is worth pointing out that the splitting of the NH signal corresponding to the *i* + 1 residue at the lowest temperature is likely due to rotamers of the *N*-Alloc group, which coalesce when the temperature increases.

Following the same protocol as that for compound **2**, the temperature coefficients for all the amide protons in derivatives **3**–**8** and **10** were extracted from VT-NMR experiments (Table 1 and Figures S2–S9). Compounds **3** (Ala), **5** (Leu), and **7** (Ser) show the same behavior as the glycine analog **2**. Only the NH^*i*+3^ proton participates in an intramolecular H-bond, suggesting the stabilization of the β-turn structure. They all contain a β-methylene group in the side chain, thus providing the necessary flexibility for the adoption of the β-turn structure. For compounds **4** (Val), **6** (Phe), and **8** (Lys), the NH^*i*+3^ temperature coefficient falls in the uncertainty range. Although we cannot fully conclude the formation of the β-turn in solution, the temperature coefficients for Phe (**6**) and Lys (**8**) are close to 3 ppb·K^–1^, suggesting some degree of β-turn folding. The valine-derived tetrapeptide model **4** clearly shows distinct behavior, with the NH^*i*+2^ forming an intramolecular bond. This is compatible with the stabilization of a γ-turn instead of a β-turn. γ-Turns are the second most common reverse turn in proteins after β-turns, formed by three consecutive residues with a H-bond between the CO^*i*^ and the NH^*i*+2^.^65^ Thus, this result demonstrates that branching at the β-position of the side chain seems to destabilize the β-turn conformation.

**Table 1:** Temperature Coefficients for NH Protons of Compounds **2**–**8** and **10**

	|Δδ/Δ*T*| (ppb/K)^b^,^c^			
compound^a^	NH^*i*+1^	NH^*i*+2^	NH^*i*+3^	NH_side-chain_
Boc-Aze(Alloc)-Gly-NHMe (**2**)	9.9	6.8	**2.0**	
Boc-Aze(Alloc)-Ala-NHMe (**3**)	8.8	6.6	**2.6**	
Boc-Aze(Alloc)-Val-NHMe (**4**)	7.1	**1.9**	3.7	
Boc-Aze(Alloc)-Leu-NHMe (**5**)	8.6	7.7	**2.8**	
Boc-Aze(Alloc)-Phe-NHMe (**6**)	8.4	7.5	3.3	
Boc-Aze(Alloc)-Ser(Bn)-NHMe (**7**)	8.5	5.6	**2.2**	
Boc-Aze(Alloc)-Lys(Z)-NHMe (**8**)	8.7	7.8	3.2	6.5
Boc-Ala-Aze(Alloc)-NHMe (**10**)	4.8	3.3	3.5	

a Aze: (3*R*,4*S*)-4-amino-4-carboxy-3-methylazepane.

b Δδ measured in DMSO-*d*_6_, range from 25 to 85 °C, samples at 7–10 mM concentration.

c The average temperature coefficients are reported when there is splitting in the NMR signals due to the existence of rotamers. Temperature coefficients equal to or lower than 3 ppb·K^–1^ are in bold.

For compound **10** with the azepane residue at the *i* + 2 position, both NH^*i*+2^ and NH^*i*+3^ have temperature coefficients in the uncertainty range, so there is no strong evidence of the formation of either a γ- or β-turn. Comparing the NMR data of **3** and **10**, the incorporation of the constrained amino acid at the *i* + 1 position seems to be more favorable for the stabilization of the β-turn, in line with the data obtained from the molecular modeling studies.

**Figure 3:**
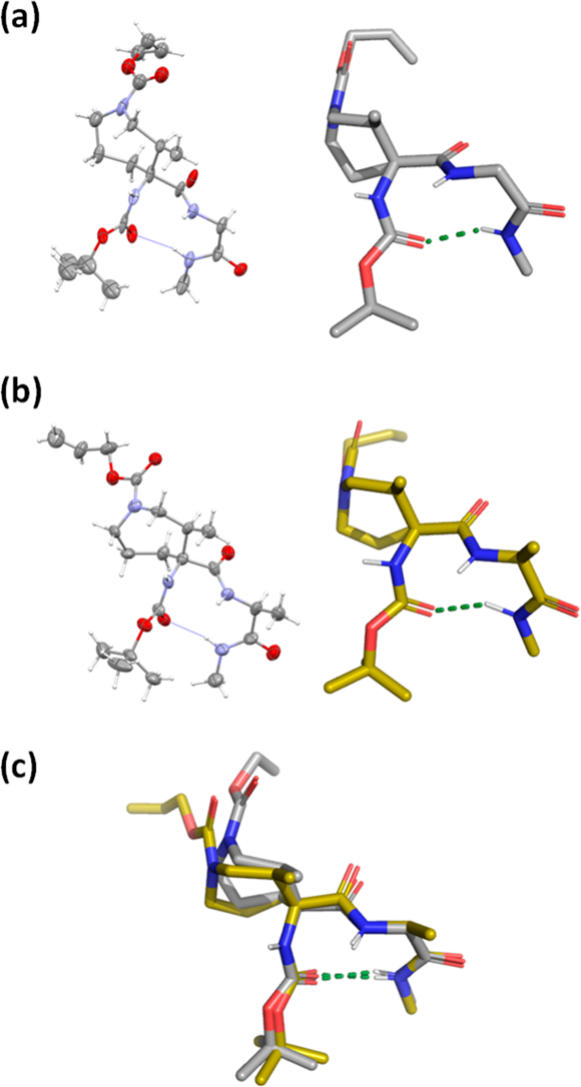
X-ray structure of a representative molecule from the asymmetric units of (a) Boc-Aze(Alloc)-Gly-NHMe **2** and (b) Boc-Aze(Alloc)-Ala-NHMe **3**, both in ORTEP (left) showing 30% probability displacement ellipsoids for non-H atoms, fixed-size spheres of radius 0.1 Å for hydrogen atoms, and stick (right) representation. (c) Superposition of structures **2** (gray) and **3** (yellow), RMSD = 0.14 (for superposition of backbone atoms). ORTEP and stick representations were created with Mercury and PyMOL, respectively.^23,66^

To gain further insight into the 3D structures of these tetrapeptide models, we attempted the crystallization of all of the prepared peptides. We obtained suitable crystals for X-ray diffraction for compounds **2** and **3**. Figure 3a and b shows the X-ray structure of a representative molecule from the asymmetric unit. In both compounds, there is an intramolecular H-bond between the amide group of the *N*-methylamide moiety (residue *i* + 3) and the Boc carbonyl oxygen (residue *i*), characteristic of a β-turn. Both β-turn structures are quite similar as observed when the two crystal structures are superimposed (Figure 3c). Deeper analysis of the asymmetric unit for both crystals allowed us to extract the topographic parameters of the observed β-turns. In particular, we measured the backbone dihedral angles of the central residues for all of the conformers (Table 2). In all cases, they are consistent with the formation of a type I β-turn. The solid-state structures of **2** and **3** are therefore in agreement with the NMR and molecular modeling data. These results confirm the ability of the azepane residue to induce β-turns in short peptides.

**Table 2:** Characteristic Torsional Angles of the Different Conformers of **2** and **3** Found in the Asymmetric Units of the Crystal Structure

compound	conformer	φ_***i***+1_	ψ_***i***+1_	φ_***i***+2_	ψ_***i***+2_
**2**	I	–58.3	–32.9	–91.9	13.1
	II	–57.8	–35.3	–85.8	10.3
	III	–59.2	–30.8	–87.1	4.3
	IV	–61.0	–27.3	–92.9	12.8
	V	–58.5	–32.7	–95.3	18.4
**3**	I	–67.3	–8.7	–106.6	–0.9
	II	–75.1	–6.7	–111.6	20.7
type I β-turn^a^		–60	–30	–90	0

a Canonical values for type I β-turns. A deviation of ±30° from these canonical values is allowed on three of these angles, and the fourth can deviate by ±45°.^67^

**Figure 4:**
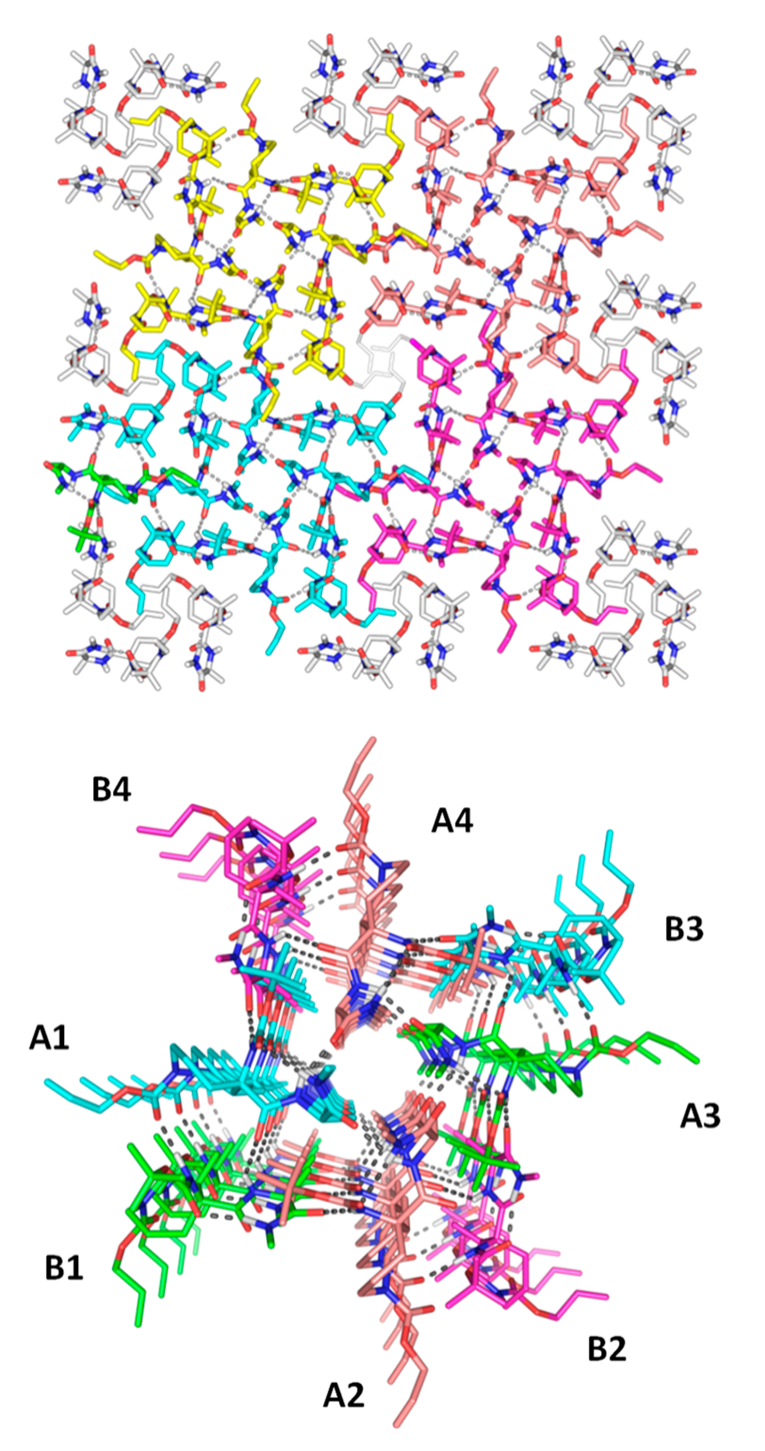
Molecular packing of Boc-Aze(Alloc)-Ala-NHMe **3**. General projection on axis *c* showing the repetitive helical arrangement (top) and detailed projection of one of the helices (bottom). For the sake of clarity, only polar hydrogens are shown. The image was created with PyMOL.^23^

The growing interest in peptide-based supramolecular materials encouraged us to analyze the molecular packing of the obtained crystal structures. For the glycine analogue **2**, the crystal belongs to the *P*2_1_ space group, with a dimeric columnar structure stabilized by a bifurcated intermolecular H-bond between CO^*i*+1^ and both the NH^*i*+1^ and NH^*i*+2^ of different molecules (see the Supporting Information for details). In contrast, the supramolecular arrangement observed in the crystal structure of peptide derivative **3** belongs to the tetragonal system. It forms supramolecular right-handed helical assemblies with small internal pores (Figure 4, top). The development of β-peptides, cyclic peptides, peptoids, and synthetic foldamers able to form helices or tubular structures has attracted great attention in the last few decades.^68−71^ However, the fabrication of supramolecular helical assemblies from short α-peptides with a defined secondary structure is relatively rare.^17−20^ In this case, it is composed by four dimers stabilized by H-bonds between the CO (Alloc) and CO^*i*+1^ of molecule A and the NH^*i*+1^ and NH^*i*+2^ of molecule B, respectively (Figure 4, bottom). In addition, the structure is stabilized by a set of interdimer H-bonds. For example, in the case of dimers A1-B1, the molecule A1 participates in one H-bond between the CO^i+2^ and the NH^*i*+2^ of A2, a second H-bond between the NH^*i*+2^ and the CO^*i*+2^ of A4, and a third H-bond between the NH^*i*+1^ and the CO^*i*+2^ of molecule B4. In addition, there is an interdimer H-bond between the CO^*i*+2^ of molecule B1 and NH^*i*+1^ of A2. Hydrophobic interactions drive the stacking of the allyl groups and packing between adjacent helices.

The analysis of the crystal packing for **2** and **3** suggests that subtle changes in the molecular structure led to significant differences in the observed supramolecular assemblies in the solid state. Considering that both compounds display a nearly identical β-turn structure in the solid state and that both crystals were grown in the same solvent under the same conditions, the changes in the intermolecular H-bond pattern are likely to be determined by the replacement of one hydrogen for a methyl group. The fact that the self-assembly properties of these peptide models could be modulated by only varying the side chain substituents opens the way to expand the present study and determine the key structural features driving the self-assembly of β-turn containing peptides. Understanding the relationship between chemical structure and self-assembly in these models could provide hints not only to study how more complex proteins assemble but also to design novel peptide-based materials for drug delivery, tissue engineering or catalysis.^72^

## Conclusions

The stabilization of the peptide secondary structure elements is of interest to achieve a fundamental understanding of complex protein–protein interactions in the search of novel therapeutic agents. In particular, β-turns are some of the most common secondary structures found in proteins. They play a key role in protein folding and stabilization as well as in protein–protein and peptide–protein recognition. Moreover, they are an attractive scaffold with relevant applications in peptide-based catalysis and in supramolecular materials. The use of constrained quaternary amino acids is one of the most common strategies used to induce reverse turns in short peptide sequences. In this context, we have previously reported the stereoselective synthesis of a novel azepane-derived quaternary amino acid.^53^ Here, we present a systematic study of its ability to stabilize β-turns in the shortest possible peptide models. The effect of the position of the azepane and peptide sequence has been assessed using molecular modeling and NMR and X-ray crystallography. We have demonstrated that the azepane amino acid is an effective β-turn inducer when incorporated preferentially at the *i* + 1 position of tetrapeptide models. The same effect was observed with other amino acids at the *i* + 2 position, such as alanine, leucine, and serine. Only branching at the β-position of the *i* + 2 side chain (valine) seems to largely disrupt the β-turn. We have also paid attention to the supramolecular self-assembly behavior of two β-turn-containing peptide models in the solid state. When alanine is incorporated at the *i* + 2 residue, the tetrapeptide model self-assembles into a supramolecular right-handed helical structure, stabilized by a defined network of intermolecular H-bonds along with the characteristic β-turn intramolecular H-bond. The results here presented therefore provide the basis for the use of azepane quaternary amino acids as effective β-turn inducers with widespread potential applications in the development of novel bioactive molecules, catalysts, and biomaterials.

## Experimental Section

### Synthesis of Compounds **2** and **3**

#### Boc-Aze(Alloc)-Gly-NHMe (**2**)

A solution of the azepane-derived amino acid **1** (0.098 g, 0.28 mmol) in dry THF (9 mL) was treated with H-Gly-NHMe·TFA (0.120 g, 0.59 mmol), (benzotriazol-1-yloxy)tripyrrolidinophosphonium hexafluorophosphate (PyBOP, 0.287 g, 0.55 mmol), and triethylamine (0.152 mL, 1.10 mmol). After the mixture was stirred at room temperature for two days, the solvent was evaporated to dryness. The crude was dissolved in EtOAc and washed successively with 10% aq. soln. citric acid (2×), 10% aq. soln. NaHCO_3_ (2×), H_2_O (1×), and brine (1×). The organic phase was dried over MgSO_4_ and evaporated to dryness. The residue was purified by centrifugal thin-layer chromatography using a gradient from 200:1 to 10:1 of MeOH/CH_2_Cl_2_, yielding **2** as a white, amorphous solid (0.016 g, 14%). HPLC: *t*_R_ = 6.26 min (gradient from 15% to 95% CH_3_CN/0.1% formic acid in H_2_O/0.1% formic acid over 10 min). ^1^H NMR (400 MHz, DMSO-*d*_6_, 50 °C; peak assignments based on COSY, HSQC and HMBC spectra provided in the Supporting Information): δ 7.96 (sa, 1H, α-NH, Gly), 7.44 (bs, 1H, N*H*Me), 6.95 (bs, 1H, 4-NH), 5.93 (ddt, 1H, *J* = 17.0, 10.5 and 5.0, 2′-H, Alloc), 5.27 (dq, 1H, *J* = 17.0 and 1.5, 3′-H, Alloc), 5.18 (dq, 1H, *J* = 10.5 and 1.5, 3′-H, Alloc), 4.53 (d, 2H, *J* = 5.0, 1′-H, Alloc), 3.70 (m, 1H, α-H, Gly), 3.52–3.56 (m, 3H, 2-H, 7-H, α-H, Gly), 3.35 (m, 1H, 2-H), 3.09 (m, 1H, 7-H), 2.58 (d, 1H, *J* = 4.5, N*-*CH_3_), 2.27 (m, 1H, 5-H), 2.22 (m, 1H, 3-H), 1.69 (m, 1H, 5-H), 1.65 (m, 1H, 6-H), 1.51 (m, 1H, 6-H), 1.43 (s, 9H, CH_3_, Boc), 0.82 (d, 3H, *J* = 7.0, 3-CH_3_). ^13^C{^1^H} NMR (100 MHz, DMSO-*d*_6_, 50 °C, two rotamers, Mr/mr = 1.2:1; peak assignments based on COSY, HSQC and HMBC spectra provided in the Supporting Information): δ 173.6 and 169.3 (CONH), 155.3 (CO, Alloc, Mr), 155.6 (CO, Boc), 155.1 (CO, Alloc, mr), 133.5 (2′-C, Alloc), 116.4 (3′-C, Alloc), 78.7 (C, Boc), 64.8 (1′-C, Alloc), 63.4 (4-C), 47.2 (2-C, Mr), 46.9 (2-C, mr), 45.6 (7-C, mr), 45.3 (7-C, Mr), 42.4 (α-C), 39.1 (3-C), 30.2 (5-C, mr), 30.5 (5-C, Mr), 28.0 (CH_3_, Boc), 25.1 (N–CH_3_), 20.6 (6-C, Mr), 20.1 (6-C, mr), 13.0 (3-CH_3_). MS (ES+): *m*/*z* = 427.19 [M + H]^+^, 853.43 [2M+H]^+^. Elemental analysis: calcd (%) for C_20_H_34_N_4_O_6_ C 56.32, H 8.04, N, 13.14. Found (%): C 56.25, H 8.08, N 13.19.

#### Boc-Aze(Alloc)-Ala-NHMe (**3**)

A solution of the azepane-derived amino acid **1** (0.070 g, 0.20 mmol) in dry CH_2_Cl_2_ (7 mL) was treated with (benzotriazol-1-yloxy)tris(dimethylamino)phosphonium hexafluorophosphate (BOP, 0.133 g, 0.30 mmol), H-Ala-NHMe·HCl (0.041 g, 0.30 mmol), and triethylamine (0.083 mL, 0.60 mmol). After the reaction mixture was stirred at room temperature for 15 h, the solution was diluted with EtOAc and washed successively with 10% aq. soln. citric acid (2×), 10% aq. soln. NaHCO_3_ (2×), H_2_O (1×), and brine (1×). The organic phase was dried over Na_2_SO_4_ and evaporated to dryness. The residue was purified on a silica gel column using EtOAc/hexane (8:1) as solvent, yielding **3** as a white, amorphous solid (0.067 g, 78%). HPLC: *t*_R_ = 10.99 min (gradient from 5% to 80% CH_3_CN/0.05% TFA in H_2_O/0.05% TFA over 20 min). *Caution!* The use of BOP as coupling reagent is discouraged because of the formation of carcinogenic HMPA, with PyBOP being a convenient alternative. ^1^H NMR (400 MHz, DMSO-*d*_6_, two rotamers, Mr/mr = 1.1:1; peak assignments based on COSY and HSQC spectra provided in the Supporting Information): δ 7.83 (d, 1H, *J* = 7.0, α-NH, Ala, mr), 7.80 (d, 1H, *J* = 7.0, α-NH, Ala, Mr), 7.62 (bs, 1H, N*H*CH_3_), 7.00 (s, 1H, 4-NH, mr), 6.94 (s, 1H, 4-NH, Mr), 5.92 (ddt, 1H, *J* = 17.0, 10.5 and 5.0, 2′-H, Alloc), 5.26 (dq, 1H, *J* = 17.0 and 1.5, 3′-H, Alloc), 5.18 (dq, 1H, *J* = 10.5 and 1.5, 3′-H, Alloc), 4.52 (m, 2H, 1′-H, Alloc), 4.24 (quint., 1H, *J* = 7.0, α-H, Ala, mr), 4.23 (quint., 1H, *J* = 7.0, α-H, Ala, Mr), 3.58 (m, 1H, 7-H), 3.45 (m, 1H, 2-H), 3.29 (m, 1H, 2-H), 3.11 (td, 1H, *J* = 13.5 and 6.5, 7-H, Mr), 3.01 (td, 1H, *J* = 13.0 and 7.0, 7-H, mr), 2.56 (d, 3H, *J* = 4.5, NCH_3_), 2.30 (m, 1H, 5-H), 2.07 (m, 1H, 3-H), 1.68 (m, 1H, 5-H), 1.63 (m, 1H, 6-H), 1.46 (m, 1H, 6-H), 1.42 (s, 9H, CH_3_, Boc), 1.21 (d, 3H, *J* = 7.0, α-CH_3_, Ala), 0.82 (d, 3H, *J* = 6.8, 3-CH_3_, mr), 0.80 (d, 3H, *J* = 6.8, 3-CH_3_, Mr). ^13^C{^1^H} NMR (75 MHz, DMSO-*d*_6_; peak assignments based on COSY and HSQC spectra provided in the Supporting Information): δ 173.1 and 172.5 (CONH), 155.8 and 155.4 (CO, Alloc and Boc, Mr), 155.7 and 155.2 (CO, Alloc and Boc, mr), 133.6 (2′-C, Alloc), 116.7 (3′-C, Alloc, Mr), 116.5 (3′-C, Alloc, mr), 78.9 (C, Boc, mr), 78.8 (C, Boc, Mr), 65.0 (1′-C, Alloc), 63.7 (4-C), 48.2 (α-CH, Ala), 47.7 (2-C, mr), 47.1 (2-C, Mr), 45.5 (7-C, mr), 45.3 (7-C, Mr), 40.8 (3-C), 30.0 (5-C), 28.2 (CH_3_, Boc), 25.5 (NCH_3_), 20.8 (6-C, mr), 20.1 (6-C, Mr), 17.6 (α-CH_3_, Ala), 13.3 (3-CH_3_, Mr), 13.2 (3-CH_3_, mr). MS (ES+): *m*/*z* = 441.2 [M + H]^+^, 463.3 [M + Na]^+^, 881.7 [2M+H]^+^, 903.5 [2M+Na]^+^. Elemental analysis: calcd (%) for C_21_H_36_N_4_O_6_ C 57.25, H 8.24, N 12.72. Found (%): C 57.08, H 8.07, N 12.80.

### Variable-Temperature NMR Experiments

Solutions of compounds **2**–**8** and **10** at 7–10 mM concentration in DMSO-*d*_6_ were prepared and transferred to an NMR tube. ^1^H NMR spectra were acquired at different temperatures (25–85 and 10 °C steps). The variation of the amide proton chemical shifts (Δδ, ppb) was plotted against the variation of temperature (Δ*T*, K), and the data fitted to a straight line. The slope of the equation represents the temperature coefficient (Δδ/Δ*T*, ppb·K^–1^).

### Preparation of Single Crystals for X-ray Diffraction Analysis

#### Compound **2** [Boc-Aze(Alloc)-Gly-NHMe]

Pure compound **2** (1.5 mg) was dissolved in MeOH (1 mL) and the mixture was put in a crystallizing dish, resulting in spontaneous crystallization after 12 days at 4 °C in a closed jar. X-ray diffraction was performed at the BL13-XALOC beamline at ALBA Synchrotron with the collaboration of ALBA staff.

#### Compound **3** [Boc-Aze(Alloc)-Ala- NHMe]

Pure compound **3** (5 mg) was dissolved in MeOH (5 mL) and the mixture was put in a crystallizing dish, resulting in spontaneous crystallization after 20 days at 4 °C in a closed jar. X-ray diffraction was performed in a Bruker MicroStar 2.7 kW, with a four-circle goniometer, with κ-geometry, and Bruker CCD detector, using CuKα radiation.

## Data Availability

The data underlying this study are available in the published article and its Supporting Information.

## Supporting Information Available

General experimental details; molecular modeling methods; synthesis and characterization of compounds, including copies of ^1^H, ^13^C and 2D NMR spectra; VT NMR experimental details; and X-ray crystallography data for compounds **2** and **3** (PDF)
