# Supplementary material for: β-Turn Induction by a Diastereopure Azepane-Derived Quaternary Amino Acid
Source: J Org Chem. 2023 Sep 29;88(20):14688–96. doi: 10.1021/acs.joc.3c01689 (PMC10594656; doi:10.1021/acs.joc.3c01689)
Supplement: Supplementary file 1 — jo3c01689_si_001.pdf [file jo3c01689_si_001.pdf]

Supporting information

β-Turn induction by a diastereopure azepane-derived quaternary amino acid

Diego Núñez-Villanueva,[a]* Adrián Plata-Ruiz,[a] Ignacio Romero-Muñiz,[a,b] Ignacio Martín-Pérez,[a] Lourdes Infantes,[c] Rosario González-Muñiz,[a] Mercedes Martín-Martínez[a]*

[a] *Instituto de Química Médica (IQM-CSIC), Juan de la Cierva 3, 28006 Madrid, Spain.*
[b] *Universidad Autónoma de Madrid, Química Orgánica, Francisco Tomás y Valiente, 7, Madrid, 28049, Spain.*
[c] *Instituto de Química Física Rocasolano (IQFR-CSIC), Serrano 119, 28006 Madrid, Spain.*

E-mail: diegonunez@iqm.csic.es; mmartin@iqm.csic.es

TABLE OF CONTENTS **Page**

1. **Abbreviations** S2
2. **Molecular modelling** S3
3. **Synthesis and characterization of compounds 2-10** S5
4. **Variable temperature NMR experiments** S51
5. **X-ray crystallography** S54
6. **References** S58

1. Abbreviations

Aze: (3*R*,4*S*)-4-amino-4-carboxy-3-methylazepane.

BOP: (Benzotriazol-1-yloxy)tris(dimethylamino)phosphonium hexafluorophosphate.

PyBOP: (Benzotriazol-1-yloxy)tripyrrolidinophosphonium hexafluorophosphate.

HATU: (1-[Bis(dimethylamino)methylene]-1H-1,2,3-triazolo[4,5-b]pyridinium 3-oxide hexafluorophosphate.

Mr: major rotamer.

mr: minor rotamer.

TEA: triethylamine.

2. Molecular modelling

2.1. General details

Molecular dynamics (MD) simulations were carried out using the Amber 10 suite of programs, with the ff99SB force field.[S1] A library for azepane residues was created using the program R.E.D.-III.4 and then incorporated in AMBER. Antechamber was used to assign atom types to model dipeptides, and to calculate a set of point charges using the AM1-BCC charge model. The Hawkins, Cramer, Truhlar pairwise generalized Born model was used to simulate implicit waters. The molecules were relaxed by energy minimization and then a MD simulation was carried out during 40 ps at constant temperature (300 K). Then the system was heated to 1000 K during 350 ps and allowed to stay at this temperature for 100 ps. The structures were subsequently cooled slowly to 300 K in steps, in each step the temperature was lowered by 100 K, and the system was allowed to stay at the new temperature for 100 ps, with 200 ps extra at 300 K. The final conformation obtained was energy-refined using steepest descent followed by conjugate gradient algorithm with a final gradient of 0.001 Kcal/mol as the convergence criteria. The conformers were stored and used to start a new simulation at high temperature. This procedure afforded samples of 100 energy-minimized conformations, which were compared to each other to eliminate the identical ones. The resulting structures were visually inspected using the computer program PyMOL and analyzed using the *ptraj* analysis program within Amber.

2.2. Analysis of the influence of azepane position in tetrapeptide models upon inverse turn induction

MD simulations were performed on simplified tetrapeptide models to ascertain the influence of the position of (3*R*,4*S*)-4-amino-4-carboxy-3-methylazepane (Aze) for the induction of inverse turns. In particular, we analyzed the incorporation of Aze in positions *i*+1 and *i*+2 of alanine-based tetrapeptide systems, in which the *N*-terminal amino acid (*i*) was replaced by an acetyl group and the *C*-terminal (*i*+3) by a *N*-methylamide. To evaluate the existence of γ or β-turns a series of topographic parameters were measured: the distance between the carbonyl oxygen of the first residue and the amide proton of the third for γ-turns (CO^{i}-NH^{i+2} < 2.5 Å), or the fourth

for β turns (CO^{i}-NH^{i+3} < 2.5 Å); and the values of the dihedral angles of the turn central residues to classify the type of turn (Table S1).

Table S1. Topographic parameters for the minimum energy conformers of Ac-Aze-Ala-NHMe (**I**) and Ac-Ala-Aze-NHMe (**II**).

Tetrapeptide models[a]	Conf.	ΔE[b] (Kcal·mol^{-1})	ϕ_{i+1}	ψ_{i+1}	ϕ_{i+2}	ψ_{i+2}	d (CO^{i}-NH^{i+3}) Å	β-turn type
**Ac-Aze-Ala-NHMe**	**I-1**	0	-53.6	-19.3	-67.6	-17.1	1.95	I or III
	**I-2**	0.51	-54.2	-14.6	-132.4	14.9	2.00	I or III
**Ac-Ala-Aze-NHMe**	**I-1**	0	-57.7	-25.2	-55.1	-18.7	2.01	I or III
	**I-2**	2.71	-65.2	-12.5	-52.7	-20.2	3.09	-

[a] Aze: (3*R*,4*S*)-4-amino-4-carboxy-3-methylazepane. [b] ΔE with respect to the global minimum within each family.

The analysis of topographic parameters of the minimum energy conformers (within a +3 Kcal·mol^{-1} window from the global minimum) showed an extraordinary tendency of the azepane residue to stabilize β-turn conformations at *i*+1 position (**I**), for which 100% of the conformers stabilize type I or III β-turn (Fig. S1). Although the global minimum for the model with the azepane residue at *i*+2 position corresponds to a type I or III β-turn, a second family of conformers adopt an open β-turn.

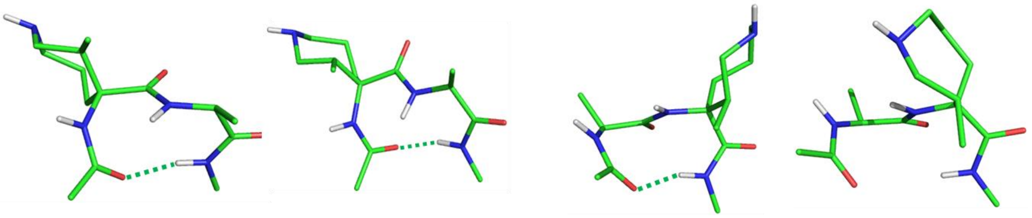

I-1 **I-2** **II-1** **II-2**

Fig S1. Minimum energy conformers for tetrapeptides models containing conformational restricted azepane derived amino acids.

2.3. Effect of the *i*+2 side chain in tetrapeptide models with Aze residue at *i*+1 position.

Using the tetrapeptide model I (Aze at position *i*+1) as reference, the alanine residue at the position *i+2* was substituted for each one of the twenty proteinogenic amino acids. Again, the conformers within a 3 Kcal·mol^{-1} window from the global minimum were analyzed to ascertain their β-turn propensity (Table S2). A high tendency to β-turn structures was observed for most of the model tetrapeptides independently of the *i*+2 amino acid, with percentages above 50%. Only for three amino acids (Gln, Asp and Arg), β-turn conformations are below this threshold due to the formation of H-bonds between this amino acid side chain and the peptide backbone. Based on this data, we prepared the corresponding tetrapeptide models for Ala, Val, Leu and Phe (> 75% induction) as well as for Ser, Lys and Gly (50-75% induction) in position i+2, in order to obtain experimental evidence on the induction of β-turn structures.

Table S2. Percentage of β-turn in tetrapeptide models (Ac-Aze-Xaa-NHMe) estimated from molecular modelling data.

Xaa	β-turn induction[a]	Xaa	β-turn induction[a]
Ala	> 75%	Glu	50-75%
Leu		Gly	
Tyr		Ile	
Trp		Ser	
Val		Thr	
Met		Lys	
Pro		Gln	< 50%
Phe		Arg	
His		Asp	
Cys			
Asn			

[a]*Percentage of reverse turns within a 3 kcal·mol^{-1} window from the global minimum.*

3. Synthesis and characterization of compounds 2-10.

3.1. General experimental details

All the reagents and materials used in the synthesis of the compounds described below were bought from commercial sources, without prior purification. THF and CH_2Cl_2 were dried and purified by standard methods.

Thin layer chromatography (TLC) was performed on aluminium sheets coated with silica gel 60 F245 (thickness 0.25 mm, Merck), with visualization by UV light (λ 245 nm). Compounds were purified by column chromatography (silica gel 60, 230-400 mesh), flash chromatography (Biotage Isolera One using prepacked 60µ Biotage or Varian silica cartridges) and centrifugal thin-layer chromatography (silica gel 60 PF254 in discs of 1-2 mm thickness). Reactions under microwave irradiation were performed in sealed vessels in a Biotage Initiator EXP EU 355301. NMR spectroscopy was carried out on a Bruker-AVANCE 300 MHz, Varian-INOVA 400 MHz, Varian-MERCURY 400 MHz or a Bruker AVANCE DRX 400 MHz instrument, using the residual solvent as the internal standard. All chemical shifts (δ) are quoted in ppm and coupling constants given in Hz. Splitting patterns are given as follows: s (singlet), bs (broad singlet), d (doublet), t (triplet), q (quadruplet), m (multiplet). Analytical HPLC was performed in an Agilent 1100 using a Sunfire C18 column (4.6 × 50 mm, 3.5 µm) or Eclipse Plus C18 column (4.6 × 150 mm, 5 µm). Mobile phase: gradient from 5% to 80% of CH_3CN (0.05% TFA) in H_2O (0.05% TFA) over 20 min, with a flow rate of 1-1.5 mL/min (UV detector at 220 or 254 nm). HPLC-MS was performed in a Waters 2695 with a reverse phase Sunfire C18 (4.6 × 50 mm, 3.5 m) connected to a Waters Micromass ZQ spectrometer. Mobile phase: gradient from 15% to 95% of CH_3CN (0.1% formic acid) in H_2O (0.1% formic acid) over 10 min, with a flow rate of 1 mL/min (UV detector at 220 or 254 nm), unless otherwise indicated. Melting points were measured in a Mettler Toledo MP70, equipped with a Leica confocal microscope and a KL 1500 LCD cold light source.

L-H-Gly-NHMe·TFA,[S2] L-H-Ala-NHMe·HCl,[S3] L-H-Val-NHMe·TFA,[S4] L-H-Leu-NHMe·TFA,[S5] L-H-Phe-NHMe·TFA,[S6] L-H-Ser(Bn)-NHMe·TFA[S7] and L-H-Lys(Z)-NHMe·TFA[S8] were prepared following known procedures. Compound **1** has been previously described.[S9]

3.2. Boc-Aze(Alloc)-Gly-NHMe (2).

^{1}H NMR (400 MHz, DMSO-d_6, 50 °C)

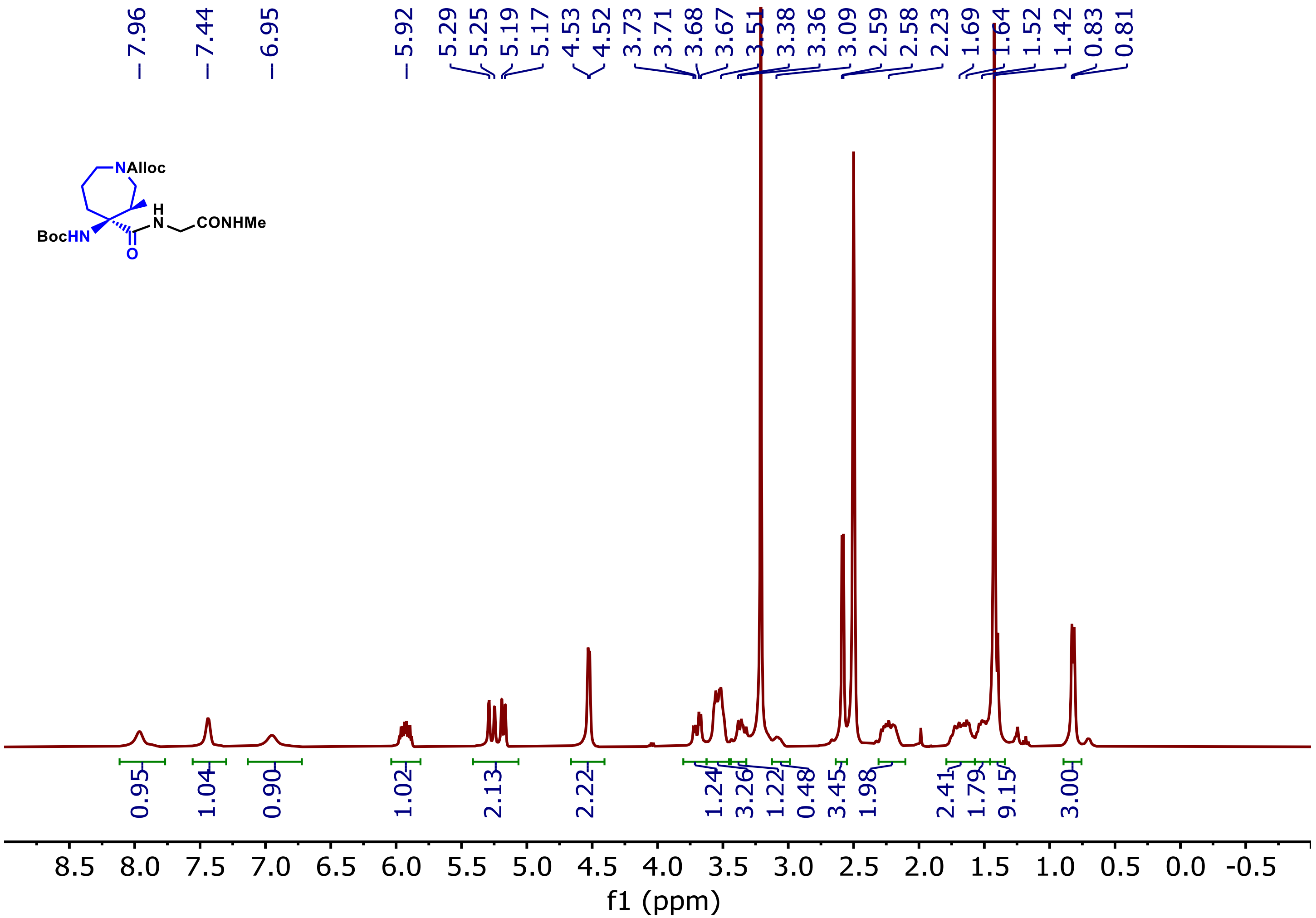

$^{13}C\{^{1}H\}$ NMR (100 MHz, DMSO-d_6, 50 °C) compound 2

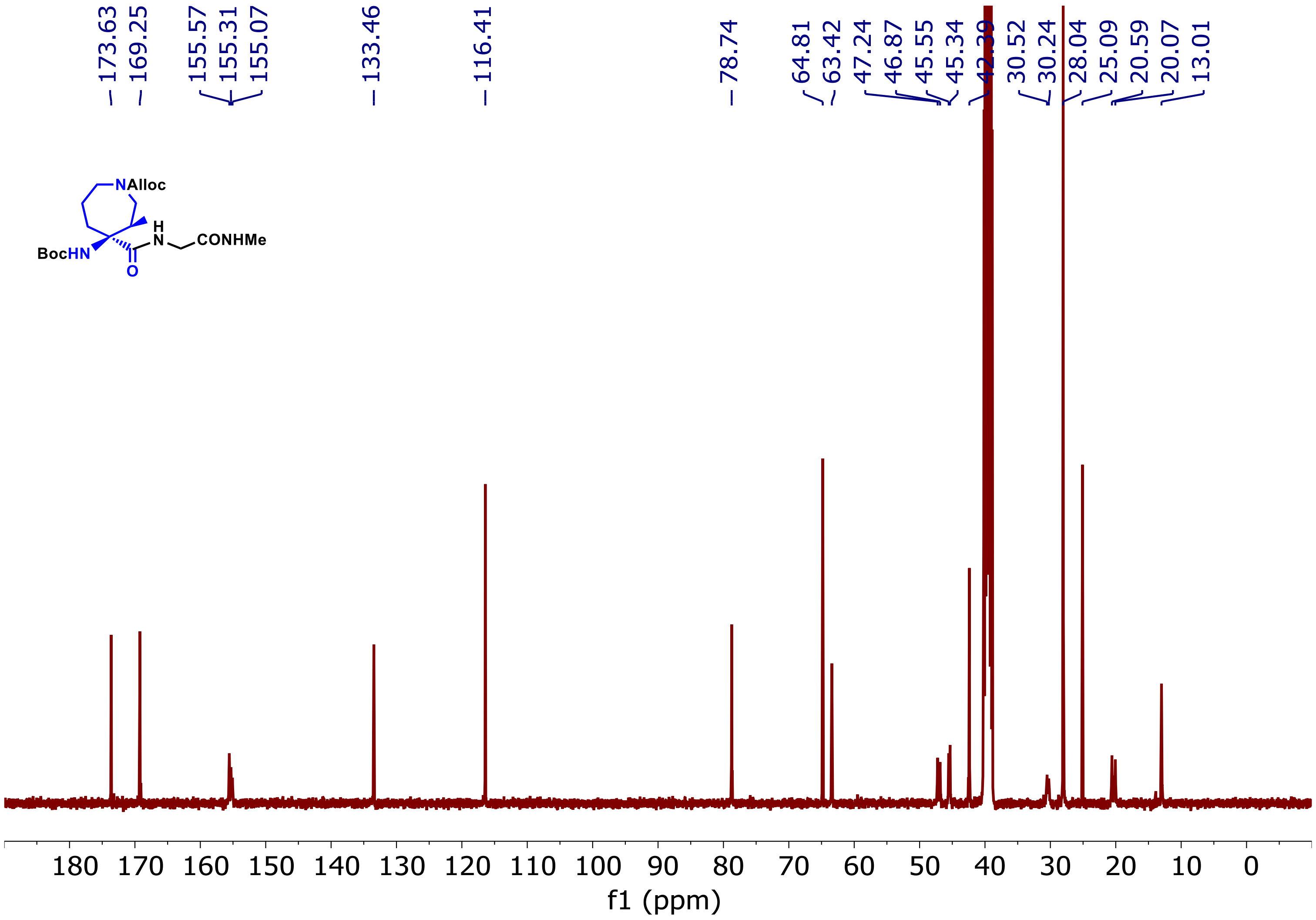

COSY compound 2

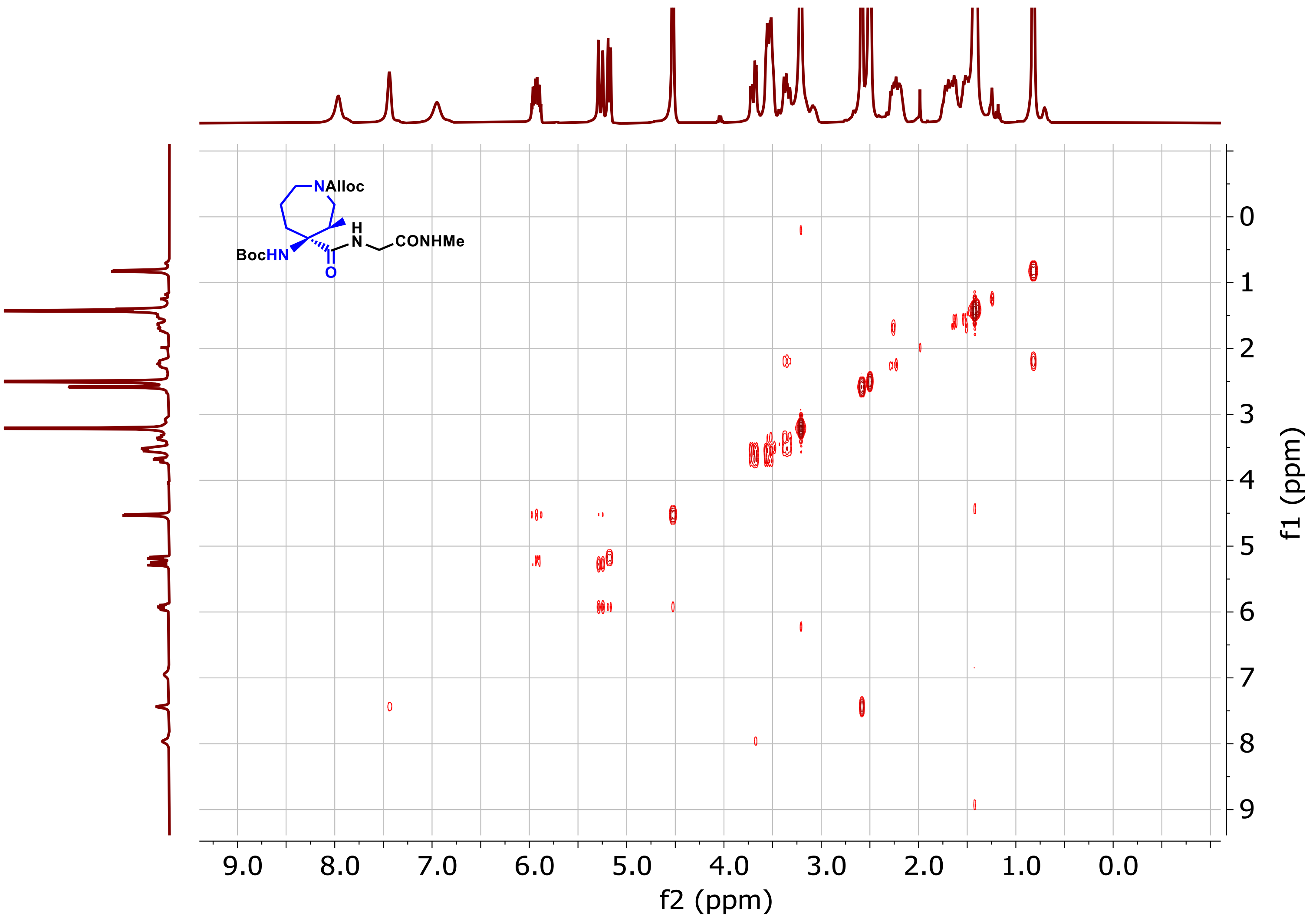

HSQC compound 2

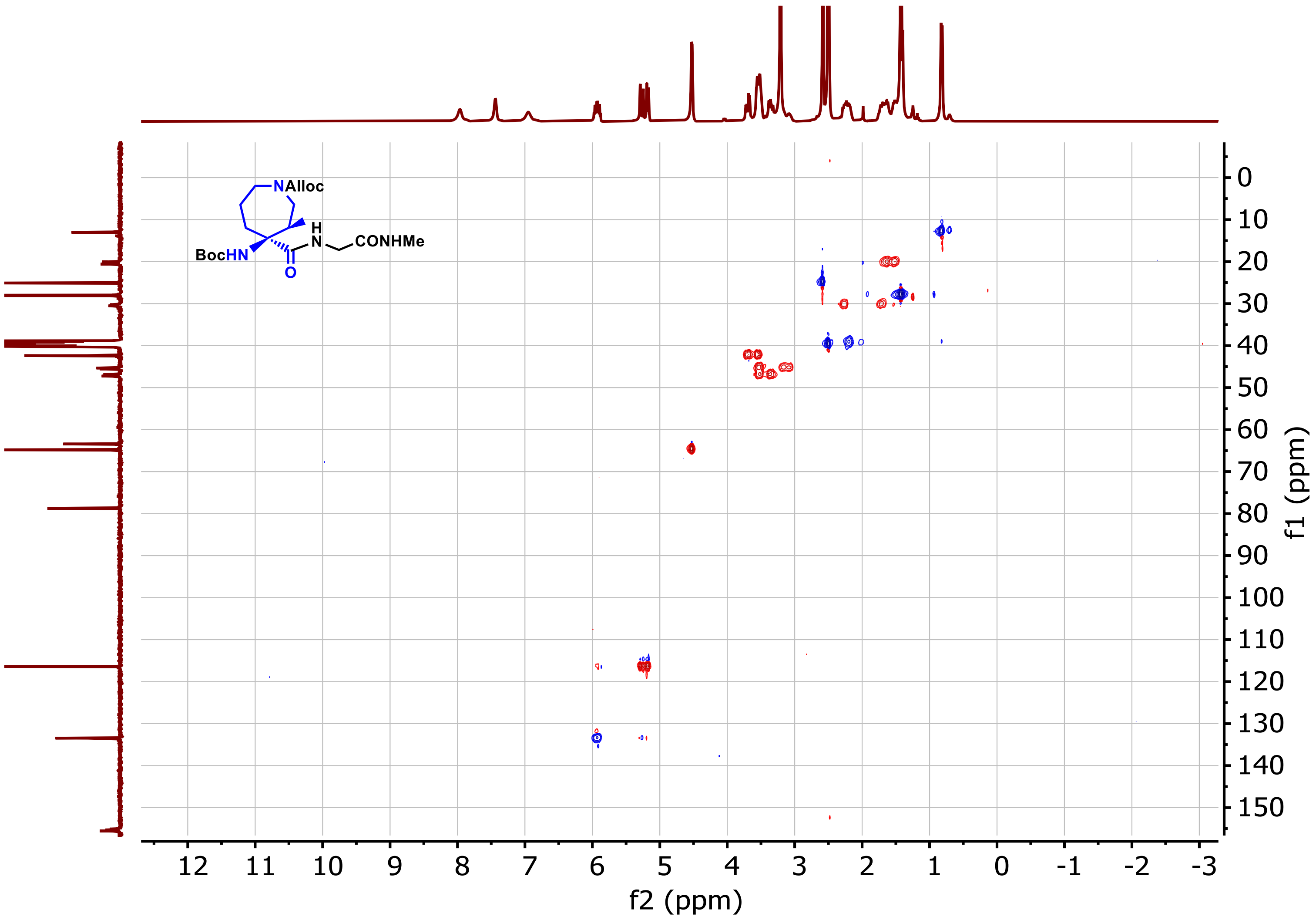

HMBC compound 2

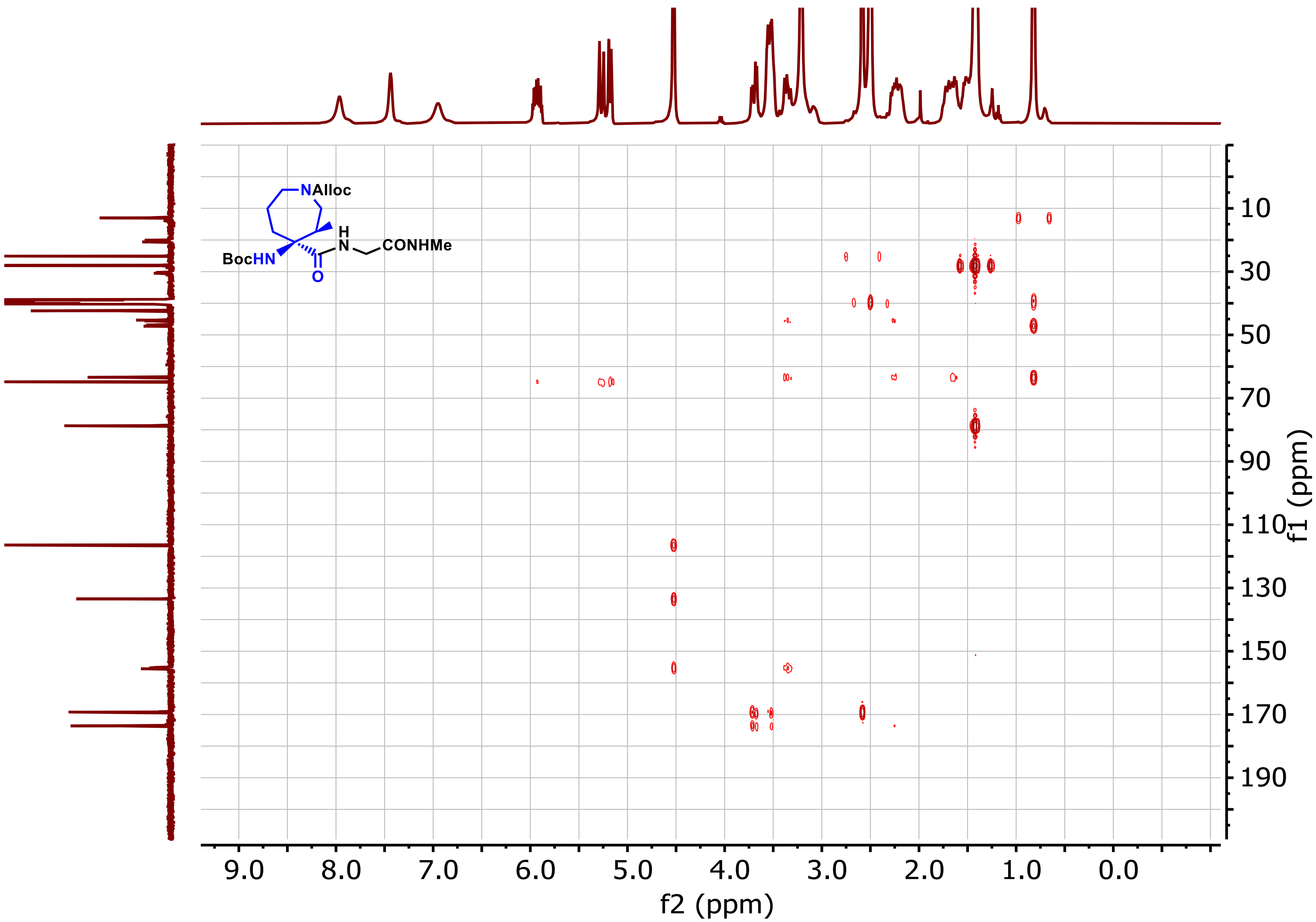

3.3. Boc-Aze(Alloc)-Ala-NHMe (3).

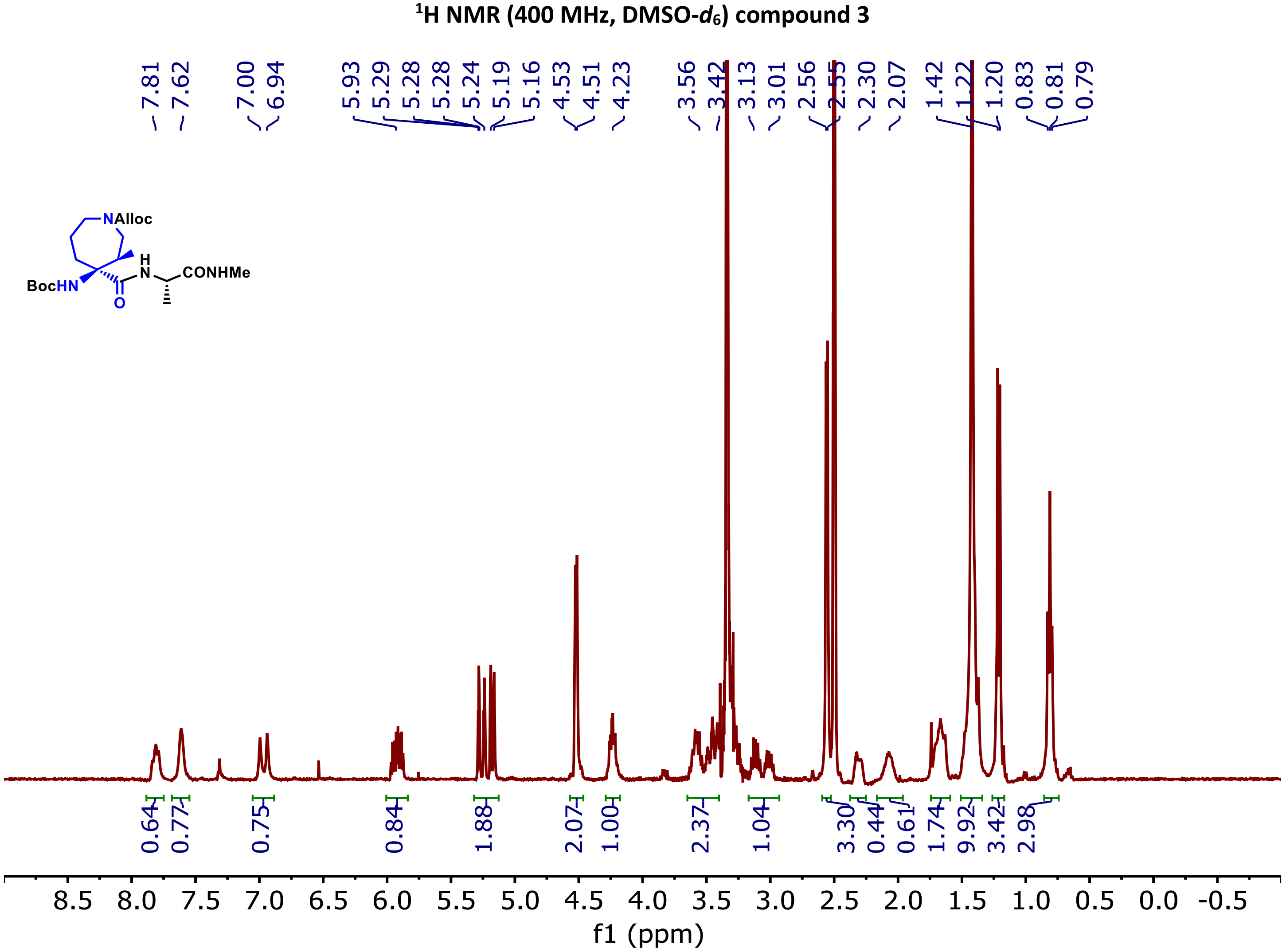

$^{13}C\{^{1}H\}$ NMR (75 MHz, DMSO-d_6) compound 3

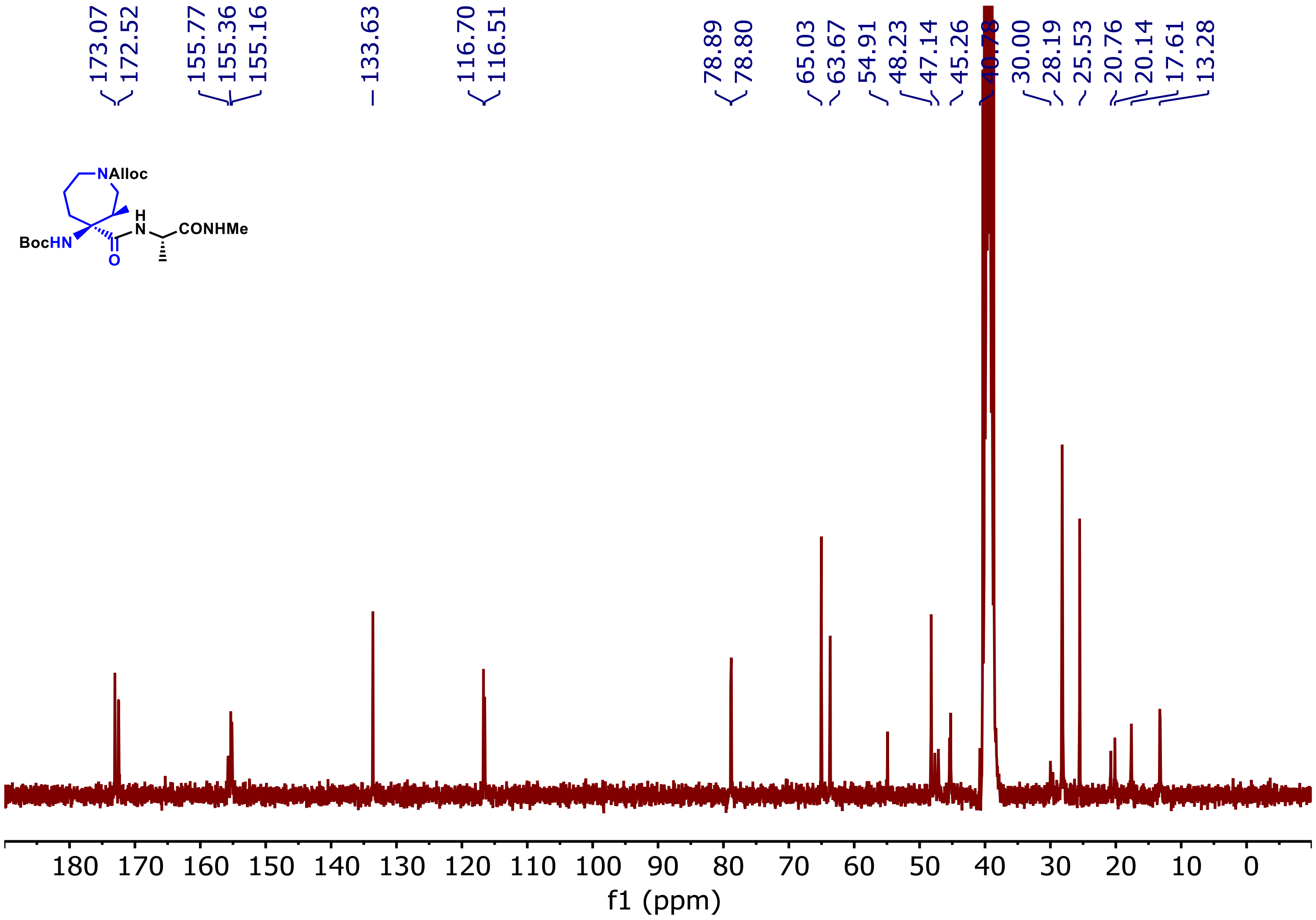

COSY compound 3

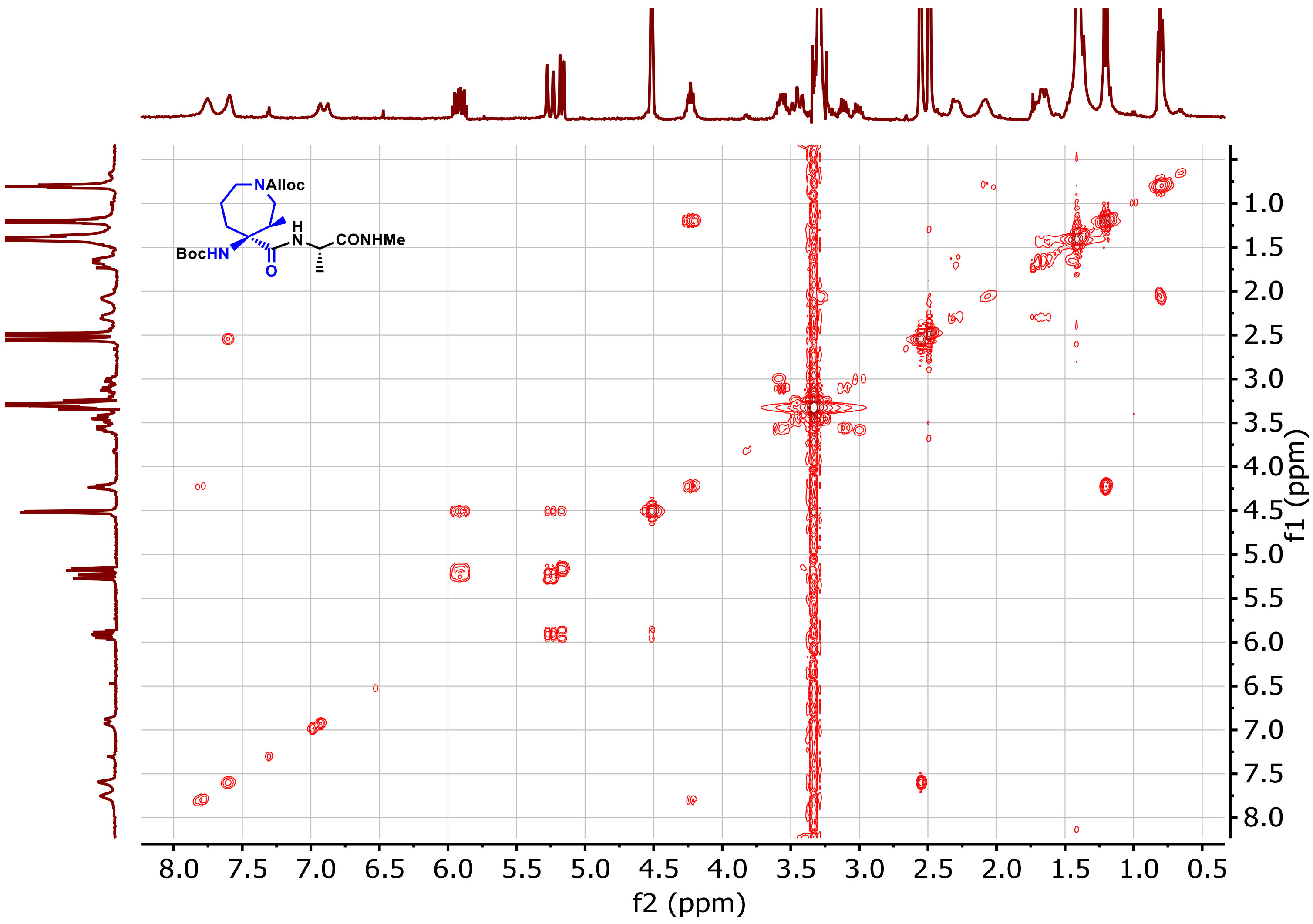

HSQC compound 3

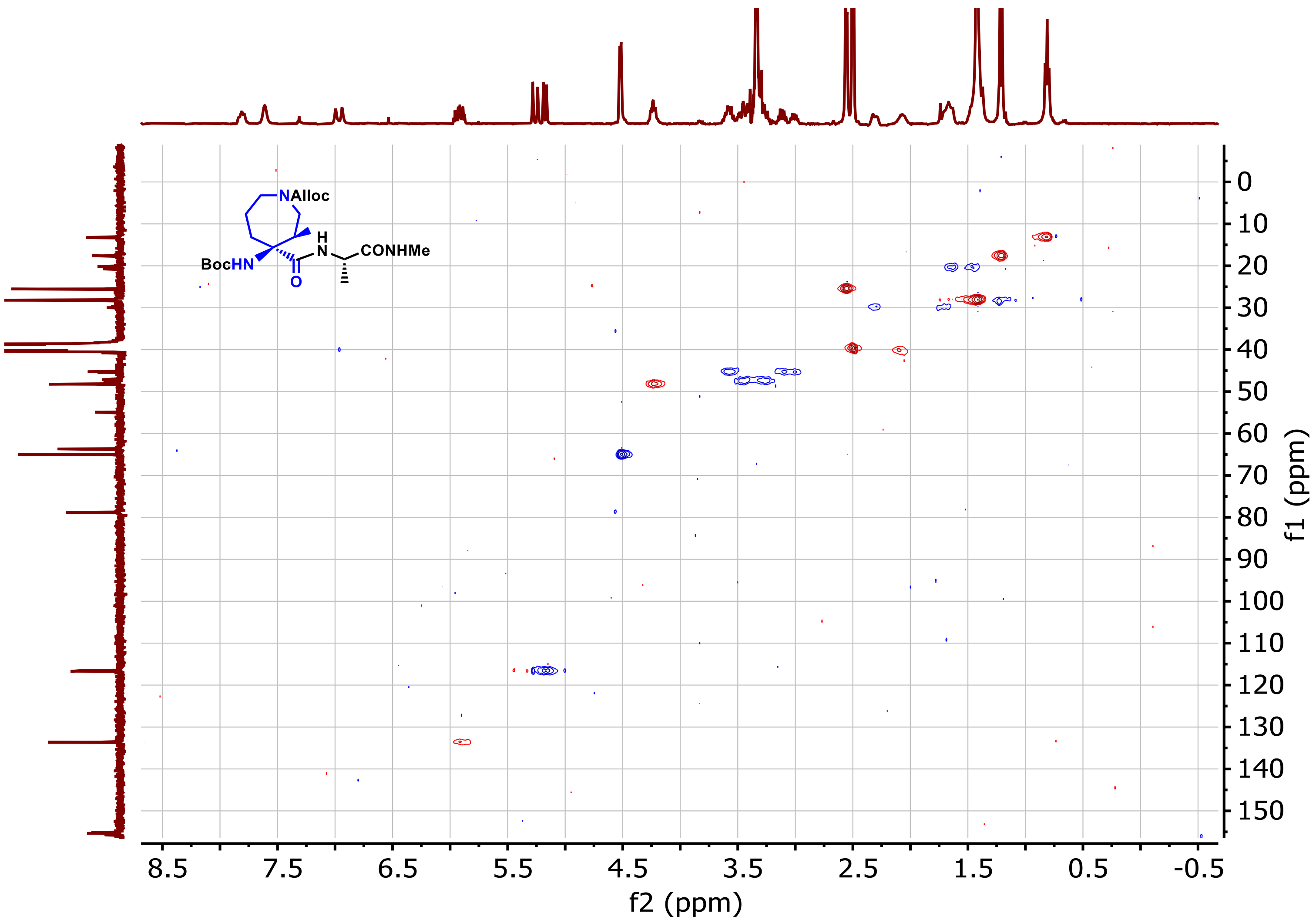

3.4. Synthesis of Boc-Aze(Alloc)-Val-NHMe (4).

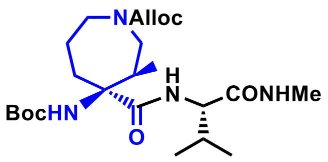

A solution of the azepane-derived amino acid **1** (0.048 g, 0.14 mmol) in dry CH_2Cl_2 (4 mL) was treated with (benzotriazol-1-yloxy)tripyrrolidinophosphonium hexafluorophosphate (PyBOP, 0.141 g, 0.27 mmol), H-Val-NHMe·TFA (0.065 g, 0.27 mmol) and triethylamine (0.076 mL, 0.54 mmol). After being stirred at room temperature for 4 days, the solution was diluted with EtOAc and washed successively with 10% aq. soln. citric acid (2x), 10% aq. soln. $NaHCO_3$ (2x), H_2O (1x) and brine (1x). The organic phase was dried over Na_2SO_4 and evaporated to dryness. The residue was purified on a silica gel column, using a gradient from 1:4 to 3:1 of EtOAc:hexane as solvent, yielding **4** as a white amorphous solid (0.029 g, 45%).

HPLC: t_R = 7.25 min (gradient from 15% to 95% of CH_3CN-0.1% formic acid in H_2O-0.1% formic acid over 10 min).

^{1}H NMR (400 MHz, $CDCl_3$, 50 °C, two rotamers, Mr/mr = 1.2:1): δ 7.05 (bs, 1H, N*H*Me, Mr), 6.97 (bs, 1H, N*H*Me, mr), 6.50 (bs, 1H, α-NH, Val), 5.95 (ddt, 1H, *J* = 17.0, 10.5 and 5.5, 2'-H, Alloc), 5.30 (dq, 1H, *J* = 17.0 and 1.5, 3'-H, Alloc), 5.22 (dq, 1H, *J* = 10.4 and 1.5, 3'-H, Alloc), 4.87 (bs, 1H, 4-NH, Mr), 4.69 (bs, 1H, 4-NH, mr), 4.62 (m, 2H, 1'-H, Alloc), 4.34 (dd, 1H, *J* = 9.0 and 4.0, α-H, Val), 3.64 (m, 1H, 2-H), 3.24 (m, 1H, 7-H), 3.22 (m, 1H, 7-H), 2.93 (m, 1H, 2-H), 2.78 (d, 3H, *J* = 5.0, NCH_3), 2.63 (m, 1H, 5-H), 2.48 (m, 2H, 3-H; β-H, Val), 2.03 (m, 1H, 5-H), 1.82 (m, 1H, 6-H), 1.55 (m, 1H, 6-H), 1.48 (s, 9H, CH_3, Boc), 0.96 (d, 6H, *J* = 7.0, 3-CH3; β-CH_3, Val), 0.84 (d, 3H, *J* = 7.0, β-CH_3, Val).*

^{13}C{^{1}H} NMR (100 MHz, $CDCl_3$, 50 °C): δ 173.4 and 171.6 (CONH), 156.5 and 156.1 (CO, Alloc and Boc), 133.2 (2'-C, Alloc), 117.6 (3'-C, Alloc), 81.6 (C, Boc), 66.4 (1'-C, Alloc), 65.7 (4-C), 58.9 (α-CH, Val), 48.5 (2-C, Mr), 47.8 (2-C, mr), 46.6 (7-C), 40.9 (3-C), 32.1 (5-C), 29.3 (β-C, Val), 28.4 (CH_3, Boc), 26.2 (NCH_3), 22.6 (6-C), 20.0 (β-CH_3, Val), 15.1 (3-CH_3, Mr), 14.6 (3-CH_3, mr).*

MS (ES+): *m/z* = 469.20 $[M+H]^+$, 937.62 $[2M+H]^+$.

Elemental analysis: calcd (%) for $C_{23}H_{40}N_4O_6$: C 58.95, H 8.60, N 11.96. Found (%): C 58.92, H 8.56, N 12.01.

* 2D methods used for NMR peak assignments: TOCSY and HSQC (spectra provided)

^{1}H NMR (400 MHz, $CDCl_3$, 50 °C) compound 4

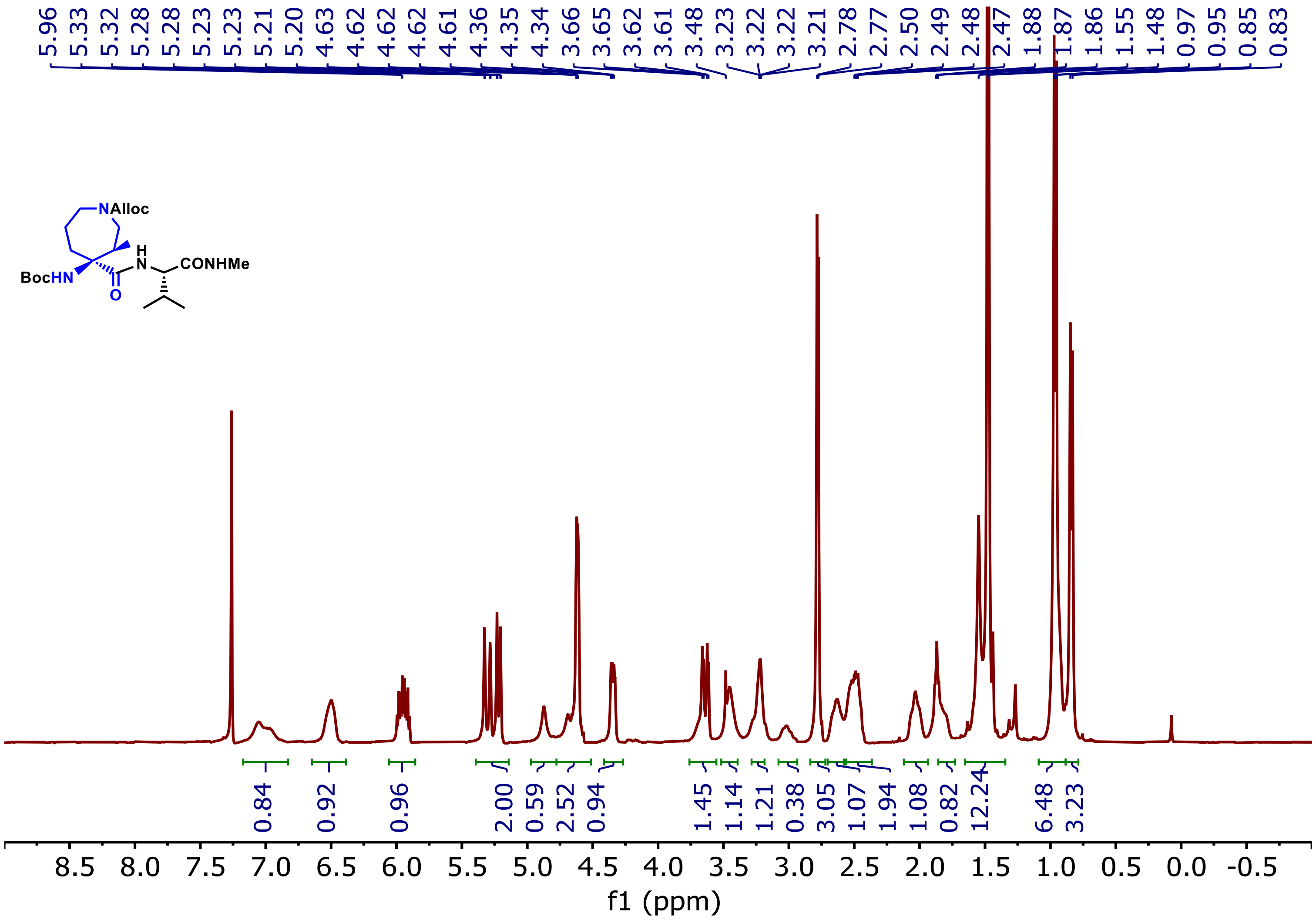

$^{13}C\{^{1}H\}$ NMR (100 MHz, $CDCl_3$, 50 °C) compound 4

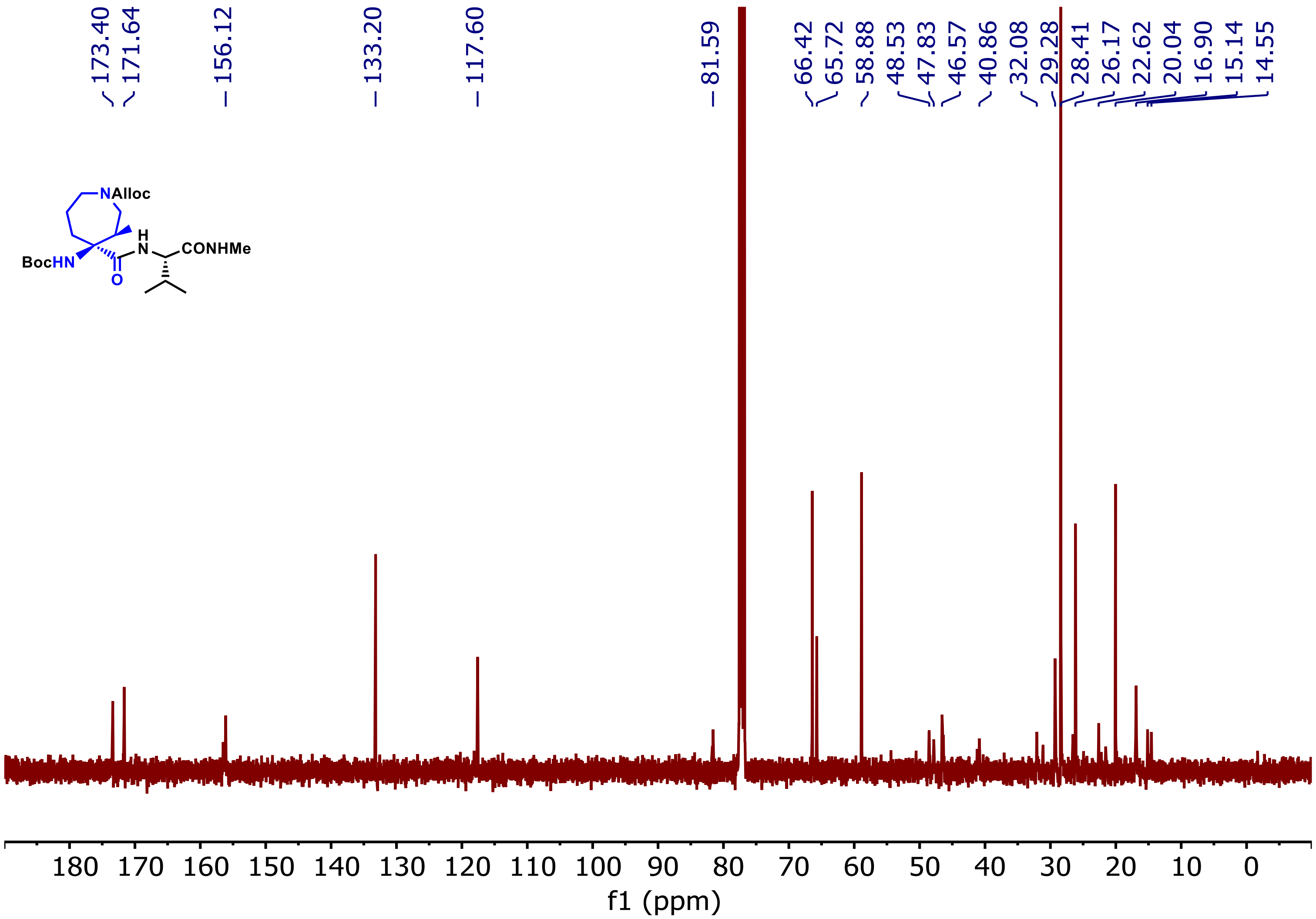

TOCSY compound 4

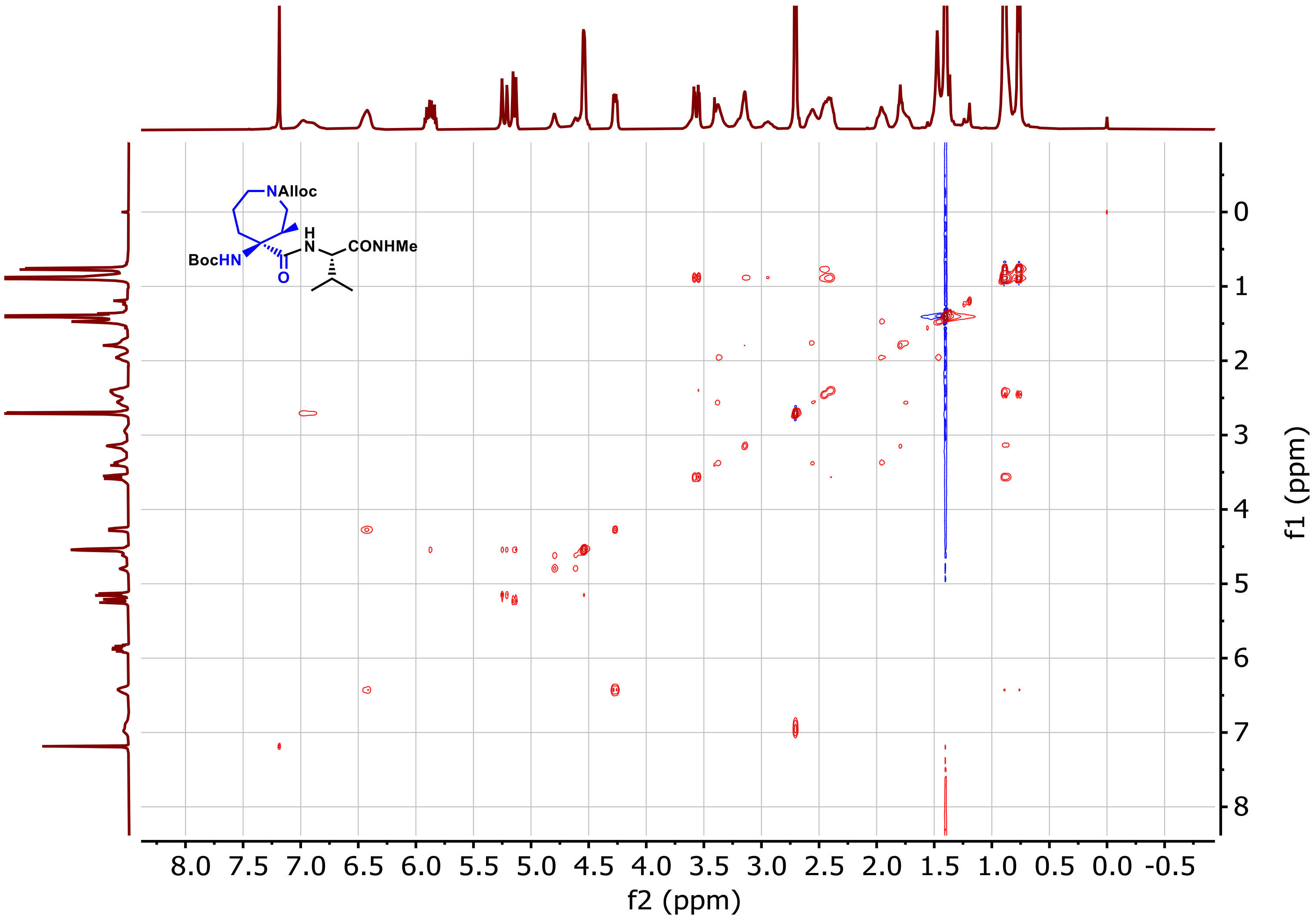

HSQC compound 4

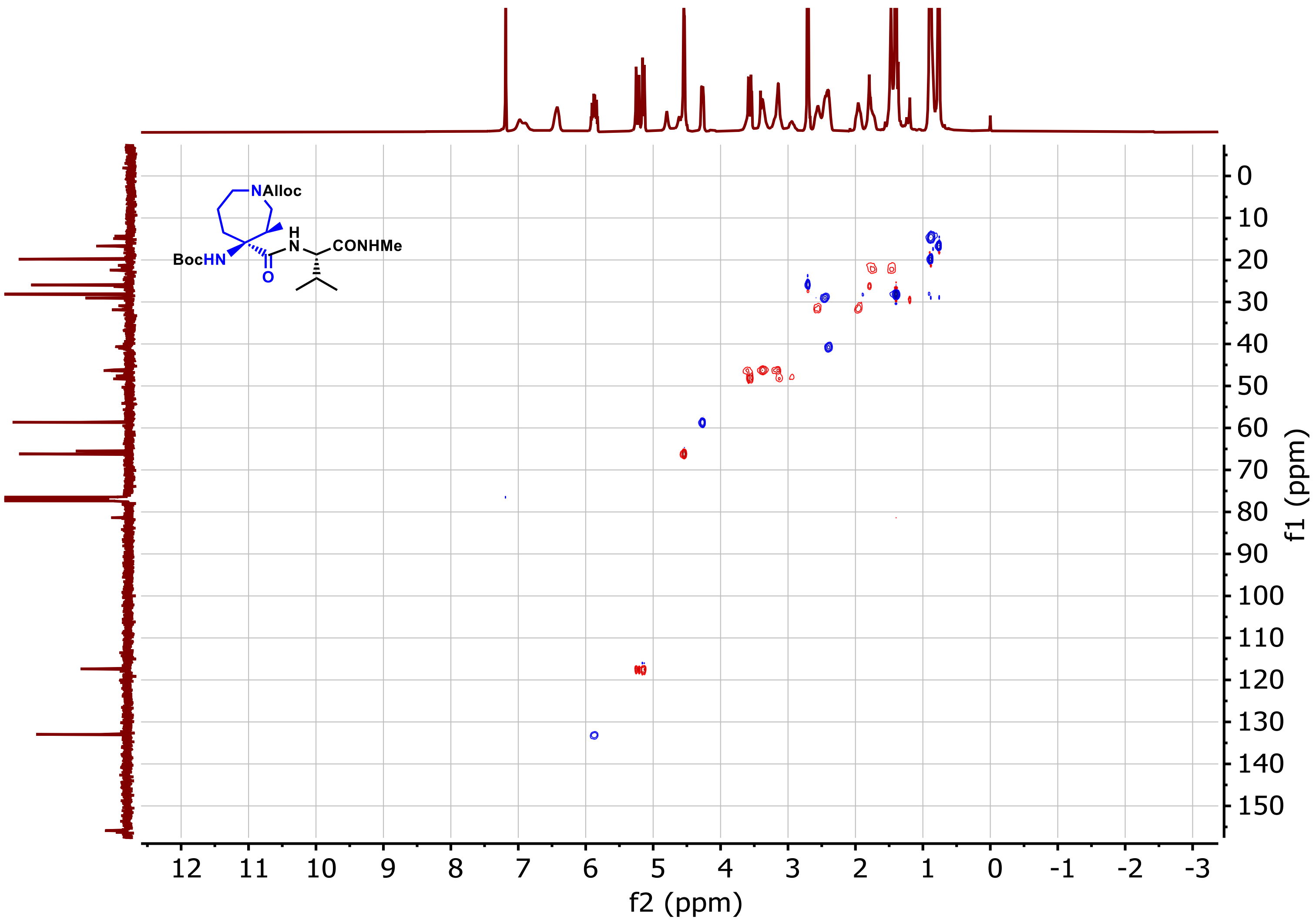

3.5. Synthesis of Boc-Aze(Alloc)-Leu-NHMe (5)

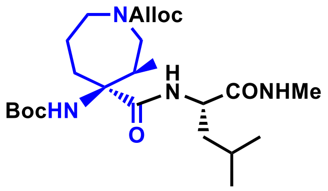

A solution of the azepane-derived amino acid **1** (0.145 g, 0.41 mmol) in dry CH_2Cl_2 (15 mL) was treated with (benzotriazol-1-yloxy)tripyrrolidinophosphonium hexafluorophosphate (PyBOP, 0.423 g, 0.81 mmol), H-Leu-NHMe·TFA (0.210 g, 0.81 mmol) and triethylamine (0.225 mL, 1.62 mmol). After being stirred at room temperature for 3 days, the solution was diluted with EtOAc and washed successively with 10% aq. soln. citric acid (2x), 10% aq. soln. $NaHCO_3$ (2x), H_2O (1x) and brine (1x). The organic phase was dried over Na_2SO_4 and evaporated to dryness. The residue was purified on a silica gel column, using a gradient from 1:4 to 3:1 of EtOAc:hexane as solvent, yielding **5** as a white amorphous solid (0.021 g, 11%).

HPLC: t_R = 7.79 min (gradient from 15% to 95% of CH_3CN-0.1% formic acid in H_2O-0.1% formic acid over 10 min).

^{1}H NMR (400 MHz, DMSO-*d$_6$*, 50 °C): δ 7.61 (bs, 1H, N*H*Me), 7.59 (bs, 1H, α-NH, Leu), 6.70 (bs, 1H, 4-NH), 5.93 (ddt, 1H, *J* = 15.5, 10.5 and 5.0, 2'-H, Alloc), 5.27 (dq, 1H, *J* = 15.5 and 1.5, 3'-H, Alloc), 5.18 (dq, 1H, *J* = 10.5 and 1.5, 3'-H, Alloc), 4.53 (d, 2H, *J* = 5.0, 1'-H, Alloc), 4.24 (m, 1H, α-H), 3.54 (m, 1H, 7-H), 3.49 (m, 1H, 2-H), 3.33 (m, 1H, 2-H), 3.08 (m, 1H, 7-H), 2.56 (d, 3H, *J* = 4.5, N-CH_3), 2.27 (m, 1H, 5-H), 2.21 (m, 1H, 3-H), 1.76 (m, 1H, 5-H), 1.50-1.65 (m, 5H, β-H, γ-H, 6-H), 1.42 (s, 9H, CH_3, Boc), 0.87 (d, 3H, *J* = 6.0, δ-CH_3), 0.85 (d, 6H, *J* = 6.1, 3-CH_3, δ-CH_3).*

^{13}C{^{1}H} NMR (100 MHz, DMSO-*d$_6$*, 50 °C, two rotamers, Mr/mr = 1.2:1): δ 173.2 and 172.2 (CONH), 155.4 and 155.0 (CO, Alloc and Boc), 133.5 (2'-C, Alloc), 116.4 (3'-C, Alloc), 78.6 (C, Boc, Mr), 64.8 (1'-C, Alloc), 63.7 (4-C), 51.3 (α-CH, Ala), 47.3 (2-C, mr), 46.8 (2-C, Mr), 45.5 (7-C, mr), 45.3 (7-C, Mr), 40.2 (β-C, Leu), 39.5 (3-C), 30.3 (5-C), 28.0 (CH_3, Boc), 25.3 (N-CH_3), 24.1 (γ-CH, Leu), 22.9 (δ-CH_3), 21.0 (δ-CH_3, Leu), 20.6 (6-C, Mr), 20.1 (6-C, mr), 12.9 (3-CH_3).*

MS (ES+): *m/z* = 483.30 $[M+H]^+$, 965.83 $[2M+H]^+$.

Elemental analysis: calcd (%) for $C_{24}H_{42}N_4O_6$: C 59.73, H 8.77, N 11.61. Found (%): C 59.76, H 8.73, N 11.60.

***** 2D methods used for NMR peak assignments: TOCSY and HSQC (spectra provided)

^1H NMR (400 MHz, DMSO-d_6, 50 °C) compound 5

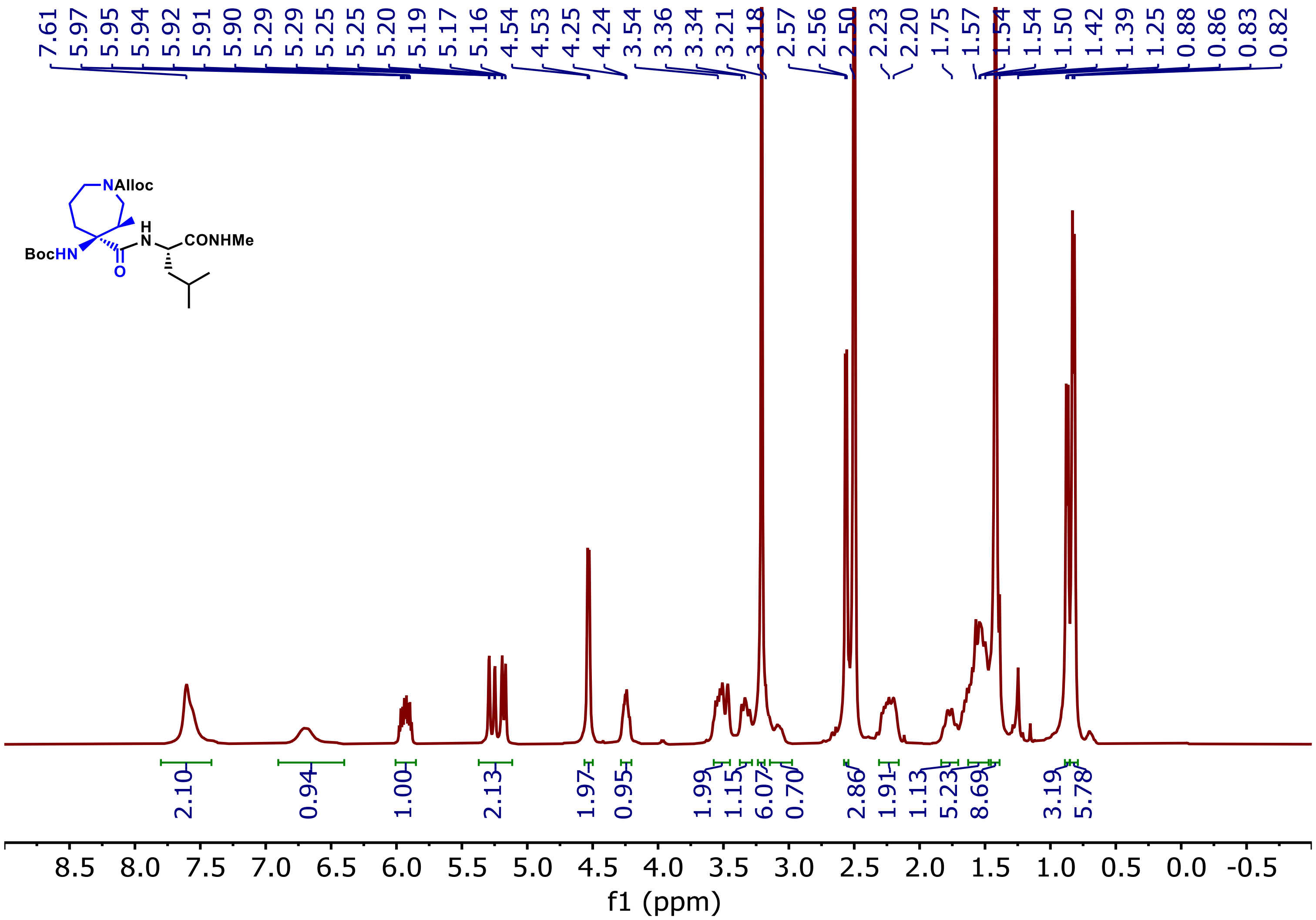

$^{13}C\{^{1}H\}$ NMR (100 MHz, DMSO-d_6, 50 °C) compound 5

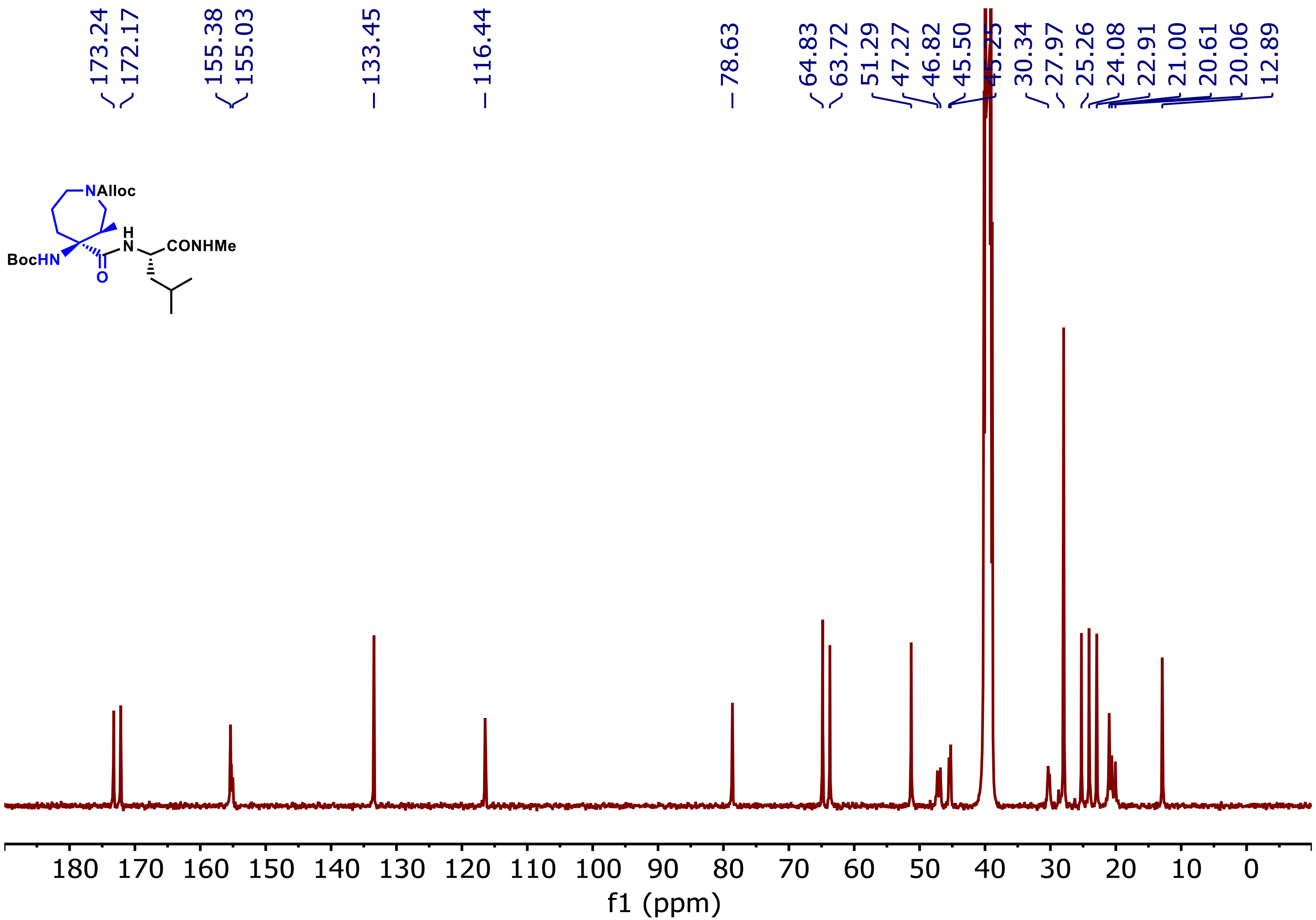

TOCSY compound 5

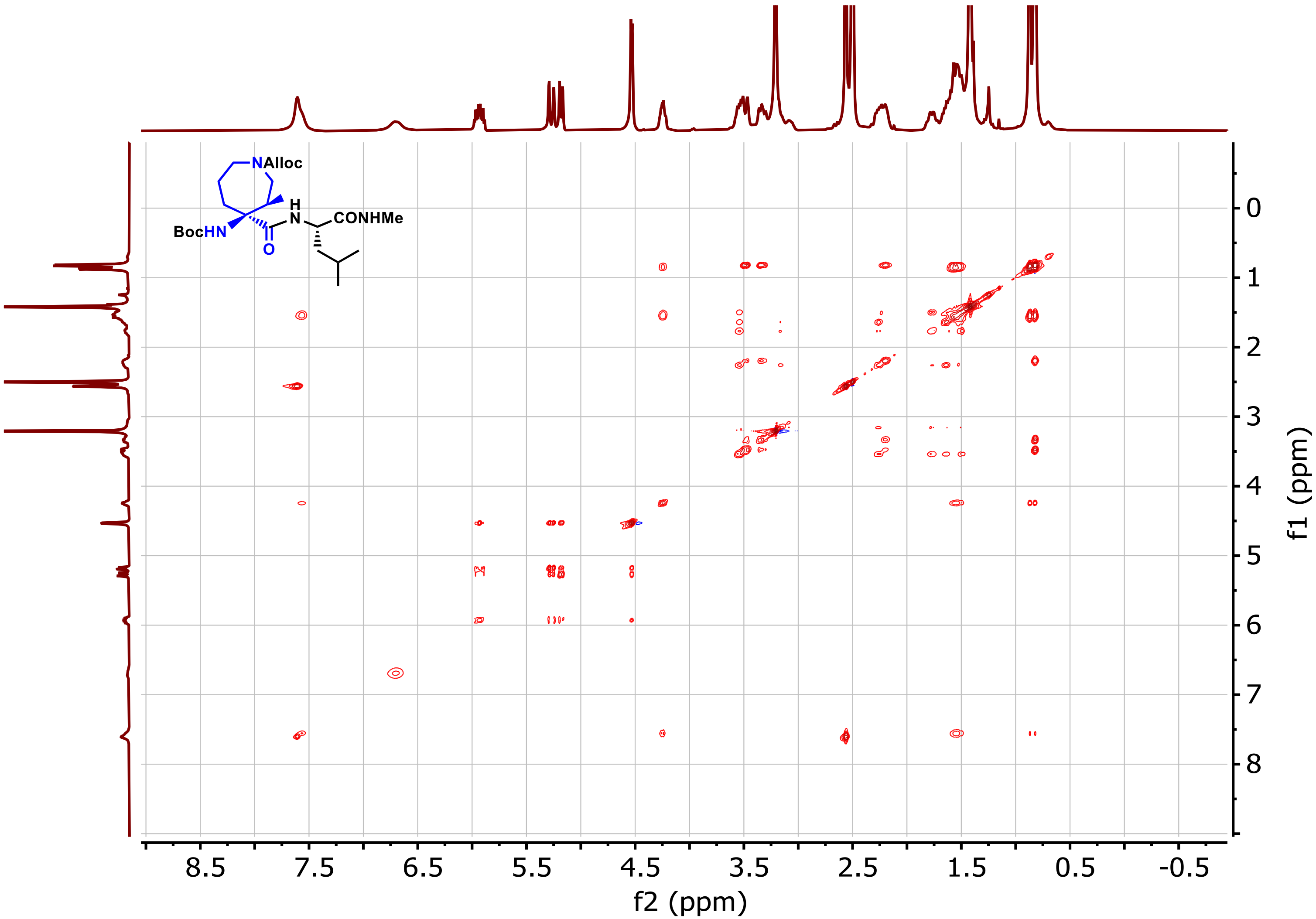

HSQC compound 5

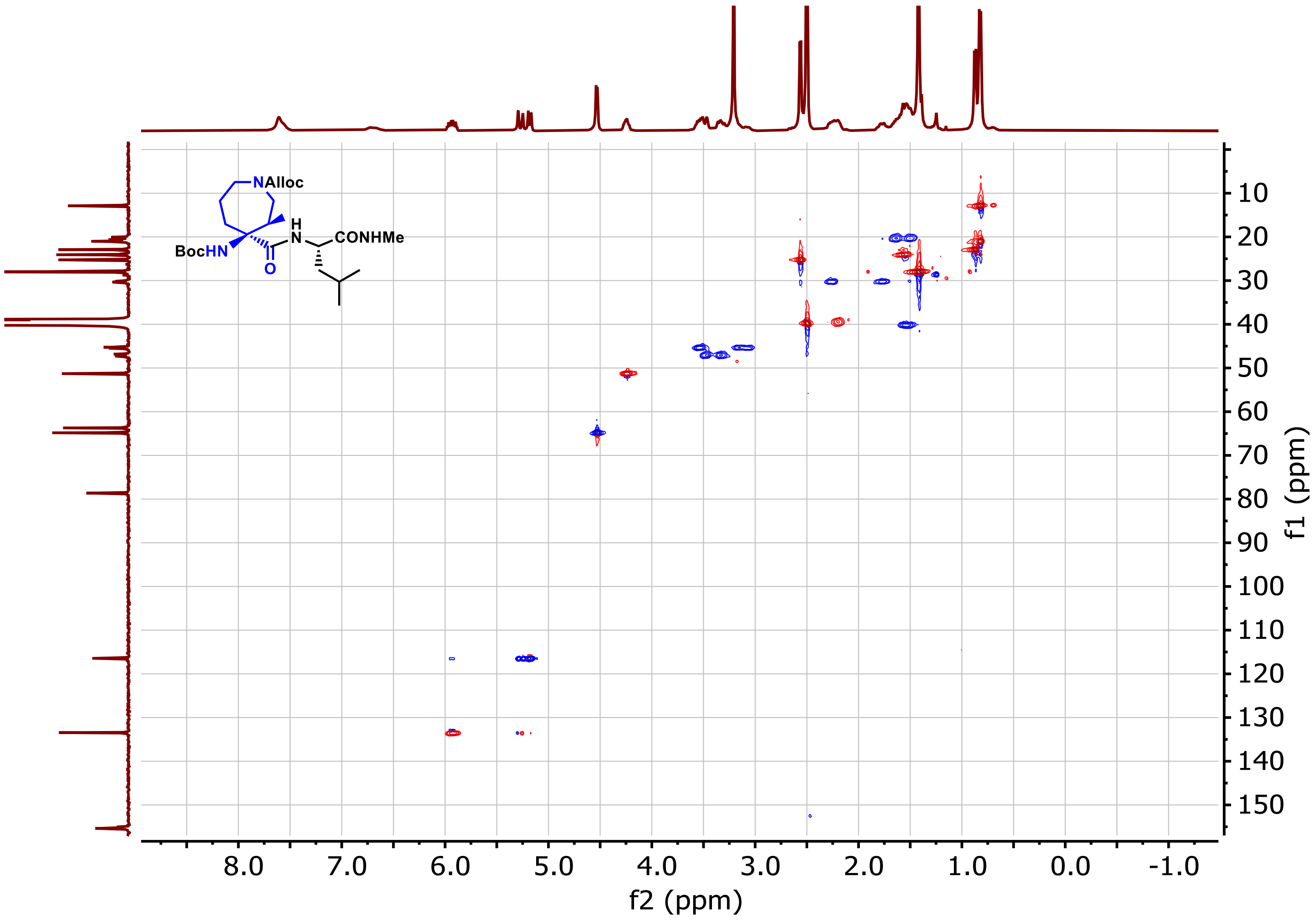

3.6. Synthesis of Boc-Aze(Alloc)-Phe-NHMe (6)

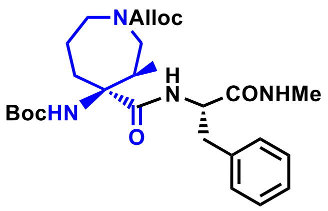

A solution of the azepane-derived amino acid **1** (0.070 g, 0.20 mmol) in dry DMF (2 mL) was treated with (1-[bis(dimethylamino)methylene]-1H-1,2,3-triazolo[4,5-b]pyridinium 3-oxide hexafluorophosphate (HATU, 0.089 g, 0.24 mmol), H-Phe-NHMe·TFA (0.056 g, 0.31 mmol) and *N*,*N*-diisopropylethylamine (0.092 mL, 0.53 mmol). After being stirred at 40 °C under microwave irradiation in a sealed vessel for 2 h, the solvent was removed under vacuum. The residue was dissolved in EtOAc and washed successively with 10% aq. soln. citric acid (2x), 10% aq. soln. $NaHCO_3$ (2x), H_2O (1x) and brine (1x). The organic phase was dried over Na_2SO_4 and evaporated to dryness. The residue was purified on a silica gel column, using a gradient from 100:1 to 10:1 of MeOH:CH_2Cl_2 as solvent, yielding **6** as a white amorphous solid (0.021 g, 20%).

HPLC: t_R = 8.04 min (gradient from 15% to 95% of CH_3CN-0.1% formic acid in H_2O-0.1% formic acid over 10 min).

^{1}H NMR (400 MHz, DMSO-*d*$_6$, 50 °C): δ 7.59 (bs, 1H, NH*Me*), 7.45 (bs, 1H, α-NH, Phe), 7.21 (m, 5H, Ph, Phe), 6.80 (s, 1H, 4-NH), 5.91 (ddt, 1H, *J* = 17.0, 10.5 and 5.0, 2'-H, Alloc), 5.25 (dq, 1H, *J* = 17.0 and 1.5, 3'-H, Alloc), 5.18 (dq, 1H, *J* = 10.5 and 1.5, 3'-H, Alloc), 4.57 (m, 1H, α-CH, Phe), 4.52 (m, 2H, 1'-H, Alloc), 3.51 (m, 1H, 7-H), 3.33 (m, 1H, 2-H), 3.25 (m, 1H, 2-H), 3.12 (m, 1H, β-CH_2, Phe), 3.04 (m, 1H, 7-H), 2.97 (m, 1H, β-CH_2, Phe), 2.58 (d, 3H, *J* = 4.5, NCH_3), 2.23 (m, 1H, 5-H), 2.07 (m, 1H, 3-H), 1.68 (m, 1H, 5-H), 1.54 (m, 1H, 6-H), 1.42 (m, 1H, 6-H), 1.39 (s, 9H, CH_3, Boc), 0.53 (d, 3H, *J* = 7.0, 3-CH_3).*

^{13}C{^{1}H} NMR (100 MHz, DMSO-*d*$_6$, 50 °C, two rotamers, Mr/mr = 1.2:1): δ 173.1 and 171.1 (CONH), 155.7 (CO, Boc), 155.2 (CO, Alloc, Mr), 154.9 (CO, Alloc, mr), 137.9 (1'-C, Ph, Phe), 133.4 (2'-C, Alloc), 128.6, 127.9 and 126.0 (Ph, Phe), 116.5 (3'-C, Alloc, Mr), 116.3 (3'-C, Alloc, mr), 78.9 (C, Boc), 64.8 (1'-C, Alloc), 63.9 (4-C), 53.7 (α-C, Phe), 47.3 (2-C, mr), 46.8 (2-C, Mr), 45.2 (7-C, mr), 45.1 (7-C, Mr), 39.3 (3-C), 36.5 (β-C, Phe), 30.1 (5-C, Mr), 29.8 (5-C, mr), 28.0 (CH_3, Boc), 25.3 (NCH_3), 20.6 (6-C, Mr), 20.0 (6-C, mr), 12.7 (3-CH_3).*

MS (ES+): *m*/*z* = 517.36 $[M+H]^+$, 1033.79 $[2M+H]^+$.

Elemental analysis: calcd (%) for $C_{27}H_{40}N_4O_6$: C 62.77, H 7.80, N 10.84. Found (%): C 62.75, H 7.84, N 10.81.

* 2D methods used for NMR peak assignments: COSY and HSQC (spectra provided)

^{1}H NMR (400 MHz, DMSO-d_6, 50 °C) compound 6

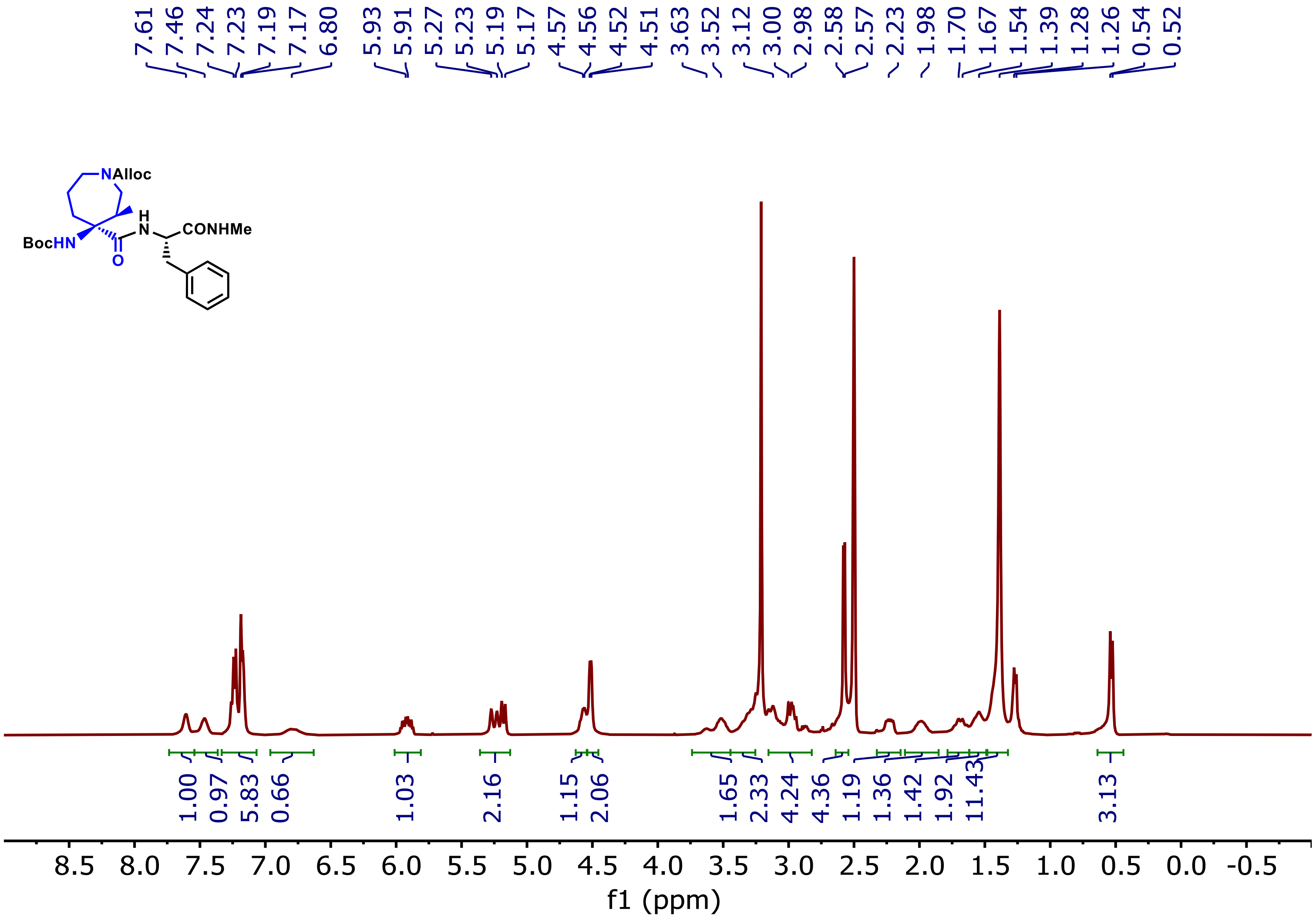

$^{13}C\{^{1}H\}$ NMR (100 MHz, DMSO-d_6, 50 °C) compound 6

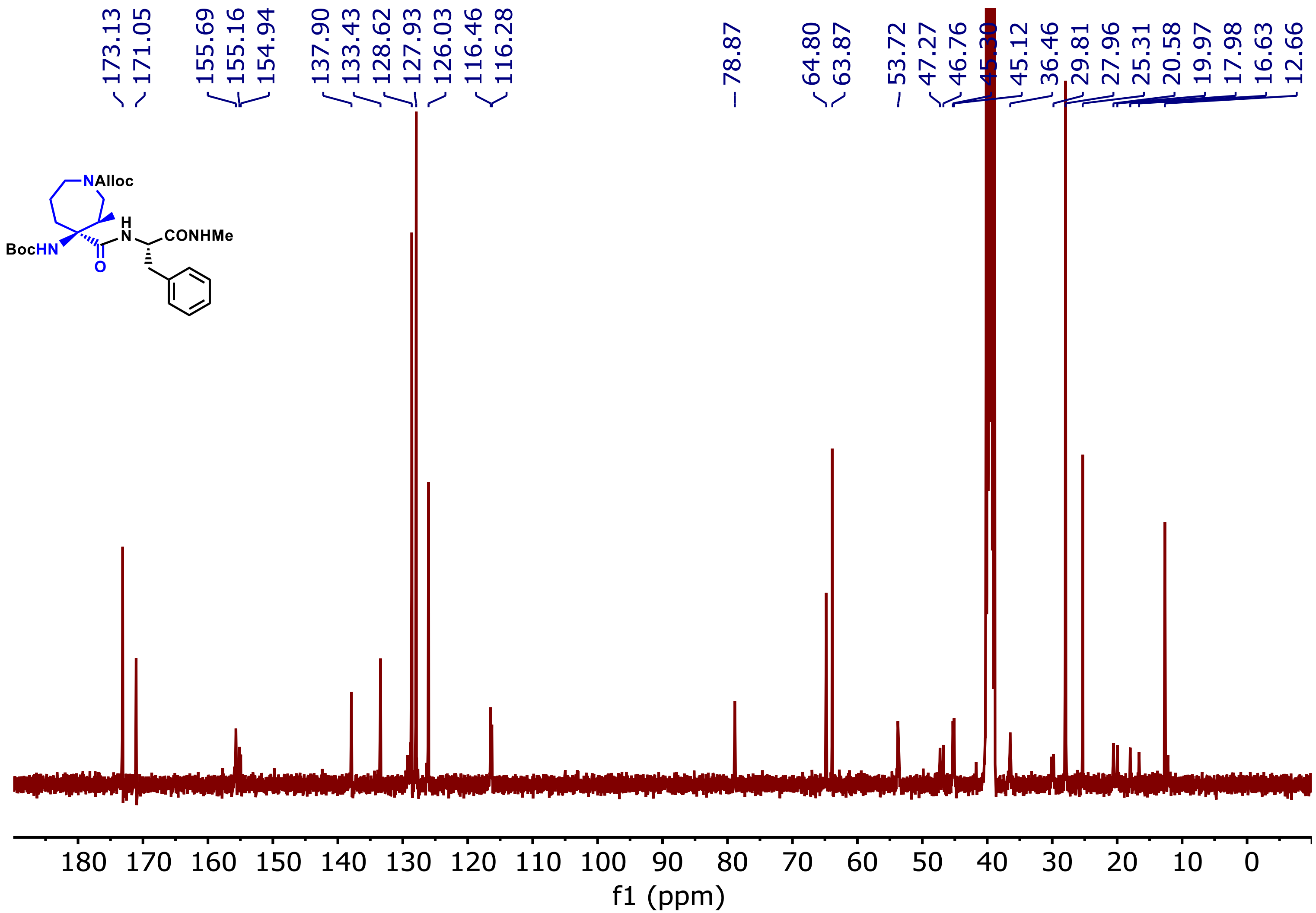

COSY compound 6

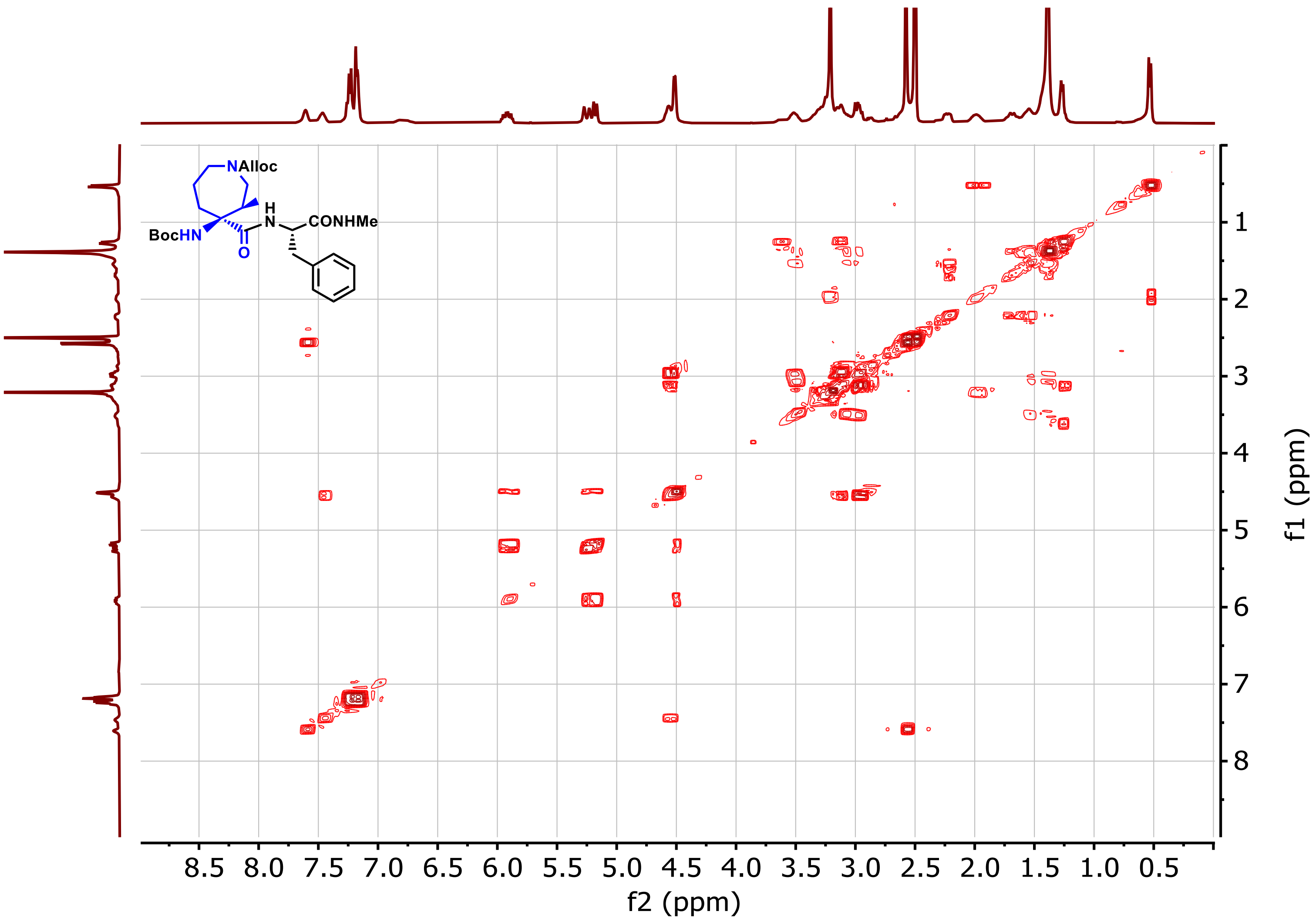

HSQC compound 6

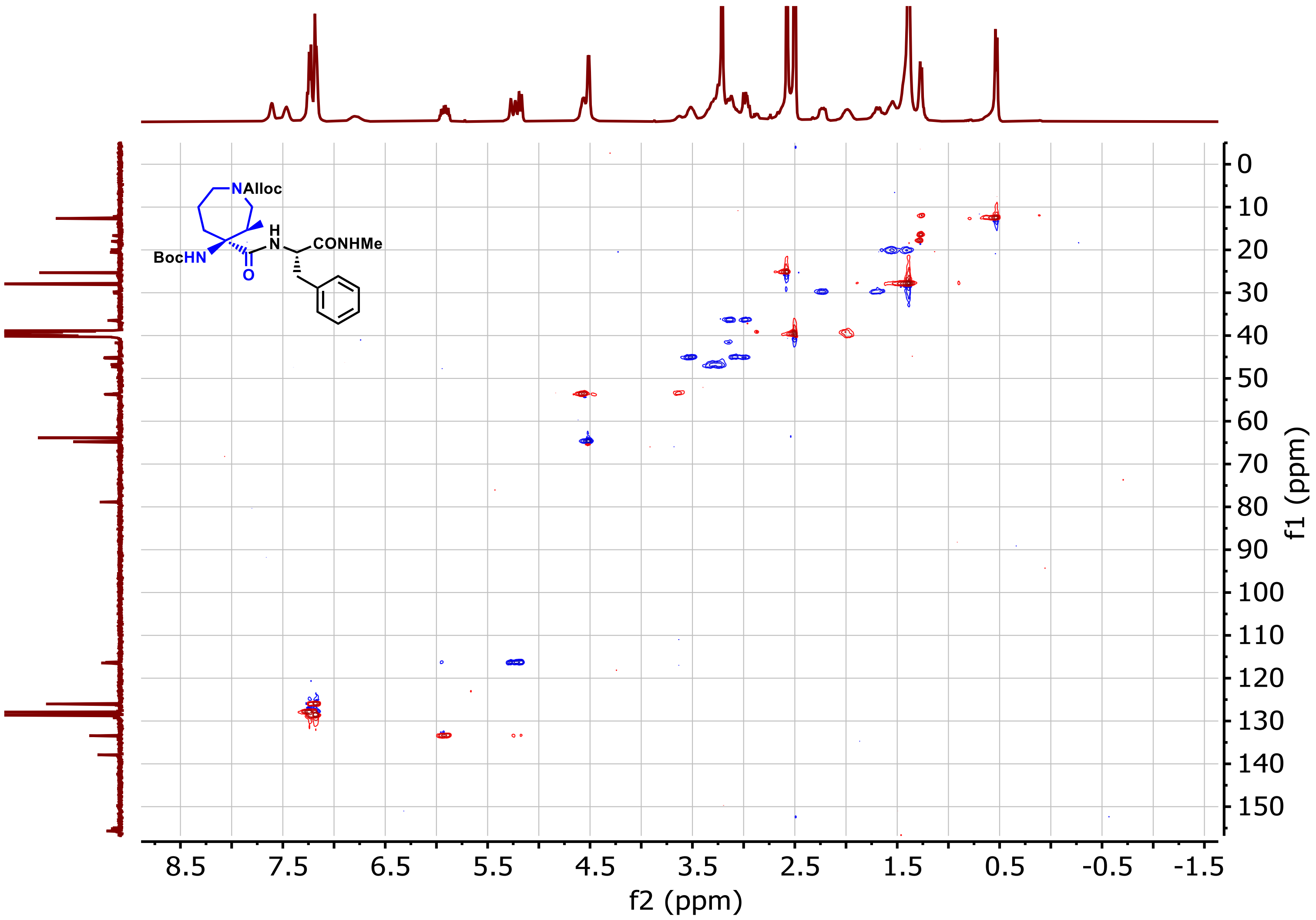

3.7. Synthesis of Boc-Aze(Alloc)-Ser(Bzl)-NHMe (7)

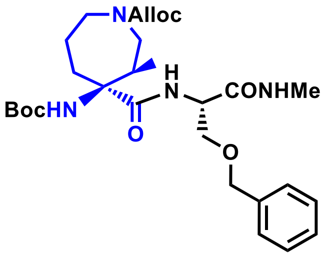

A solution of the azepane-derived amino acid **1** (0.200 g, 0.56 mmol) in dry THF (18 mL) was treated with H-Ser(Bzl)-NHMe·TFA (0.365 g, 1.13 mmol), (benzotriazol-1-yloxy)tripyrrolidinophosphonium hexafluorophosphate (PyBOP, 0.580 g, 1.13 mmol) and triethylamine (0.311 mL, 2.24 mmol). After being stirred at room temperature for 2 days, the solvent was evaporated to dryness. The crude was dissolved in EtOAc and washed successively with 10% aq. soln. citric acid (2x), 10% aq. soln. $NaHCO_3$ (2x), H_2O (1x) and brine (1x). The organic phase was dried over $MgSO_4$ and evaporated to dryness. The residue was purified by centrifugal thin-layer chromatography, using a gradient from 1:6 to 3:1 of EtOAc:hexane as solvent, yielding **7** as a white amorphous solid (0.048 g, 16%).

HPLC: t_R = 8.07 min (gradient from 15% to 95% of CH_3CN-0.1% formic acid in H_2O-0.1% formic acid over 10 min).

^{1}H NMR (400 MHz, DMSO-*d*$_6$, 50 °C): δ 7.62 (bs, 1H, N*H*Me), 7.47 (bs, 1H, α-NH, Ser), 7.36-7.26 (m, 5H, Ph, Bn), 7.00 (bs, 1H, 4-NH), 5.93 (ddt, 1H, *J* = 17.0, 10.5 and 5.0, 2'-H, Alloc), 5.27 (dq, 1H, *J* = 17.0 and 1.5, 3'-H, Alloc), 5.18 (dq, 1H, *J* = 10.5 and 1.5, 3'-H, Alloc), 4.53 (m, 2H, 1'-H, Alloc), 4.44 (m, 3H, α-CH and O-CH_2, Ser), 3.78 (m, 1H, β-H, Ser), 3.66 (m, 1H, β-H, Ser), 3.59 (m, 1H, 7-H), 3.46 (m, 1H, 2-H), 3.29 (m, 1H, 2-H), 3.07 (m, 1H, 7-H), 2.60 (d, 3H, *J* = 4.5, NCH_3), 2.35 (m, 1H, 5-H), 2.07 (m, 1H, 3-H), 1.71 (m, 1H, 5-H), 1.71 (m, 1H, 6-H), 1.46 (m, 1H, 6-H), 1.40 (s, 9H, CH_3, Boc), 0.83 (d, 3H, *J* = 7.0, 3-CH_3).*

^{13}C{^{1}H} NMR (100 MHz, DMSO-*d*$_6$, 50 °C, two rotamers, Mr/mr = 1.1:1): δ 173.1 and 169.6 (CONH), 155.7 (CO, Boc), 155.1 (CO, Alloc, Mr), 155.0 (CO, Alloc, mr), 137.9 (1'-C, Bn), 133.4 (2'-C, Alloc), 128.0, 127.2 and 127.2 (Ph, Bn), 116.5 (3'-C, Alloc, Mr), 116.3 (3'-C, Alloc, mr), 78.9 (C, Boc), 71.8 (O-CH_2,Ser), 64.8 (1'-C, Alloc), 63.9 (4-C), 69.3 (β-C, Ser), 52.6 (α-C, Ser), 47.6 (2-C, mr), 47.0 (2-C, Mr), 45.2 (7-C, mr), 45.1 (7-C, Mr), 40.3 (3-C), 29.8 (5-C, Mr), 29.5 (5-C, mr), 28.0 (CH_3, Boc), 25.3 (NCH_3), 20.7 (6-C, Mr), 20.0 (6-C, mr), 13.0 (3-CH_3).*

MS (ES+): *m/z* = 547.04 $[M+H]^+$, 1093.49 $[2M+H]^+$.

Elemental analysis: calcd (%) for $C_{28}H_{42}N_4O_7$: C 61.52, H 7.74, N 10.25. Found (%): C 61.48, H 7.80, N 10.22.

* 2D methods used for NMR peak assignments: COSY, HSQC and HMBC (spectra provided)

^{1}H NMR (400 MHz, DMSO-d_6, 50 °C) compound 7

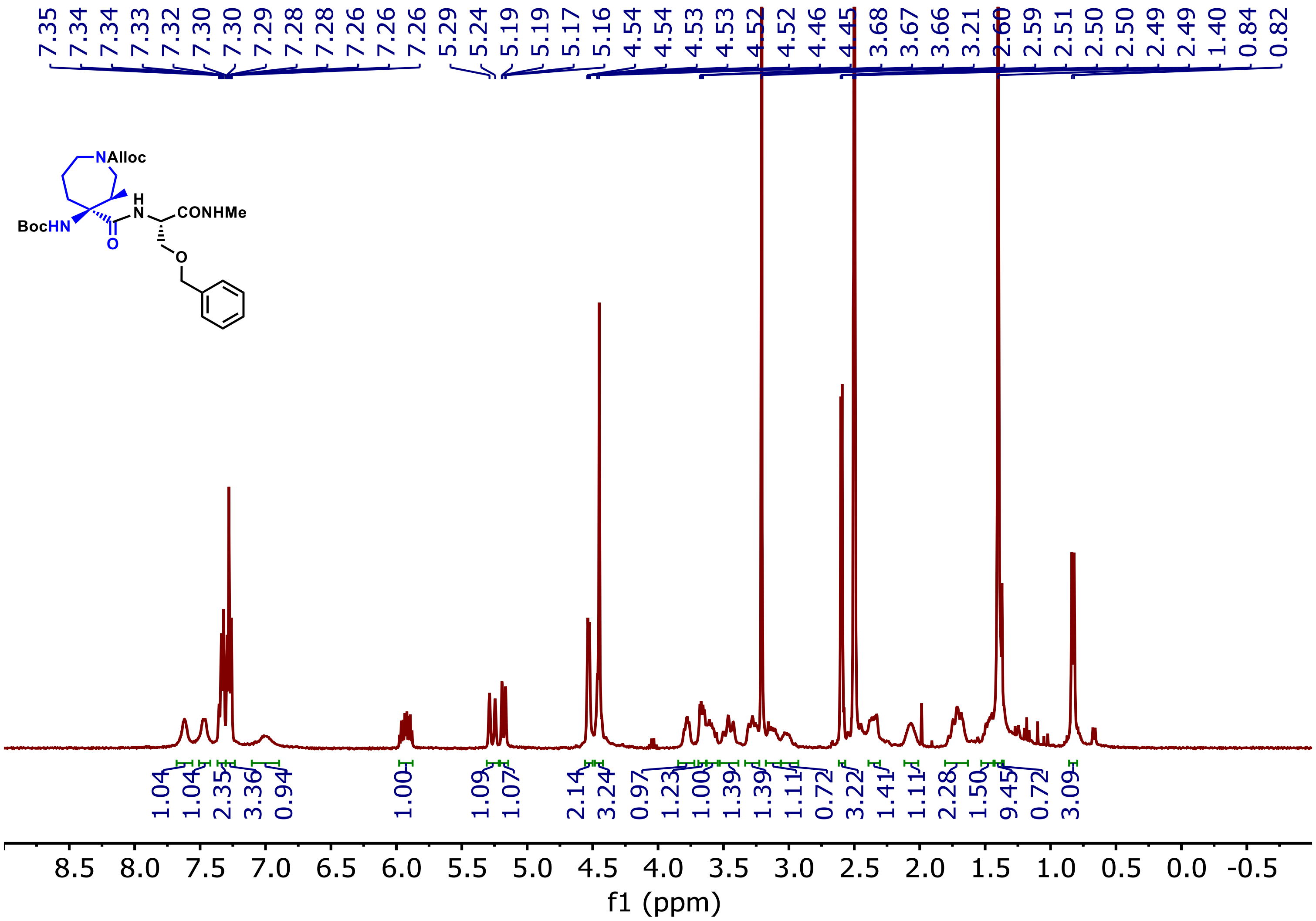

$^{13}C\{^1H\}$ NMR (100 MHz, DMSO-d_6, 50 °C) compound 7

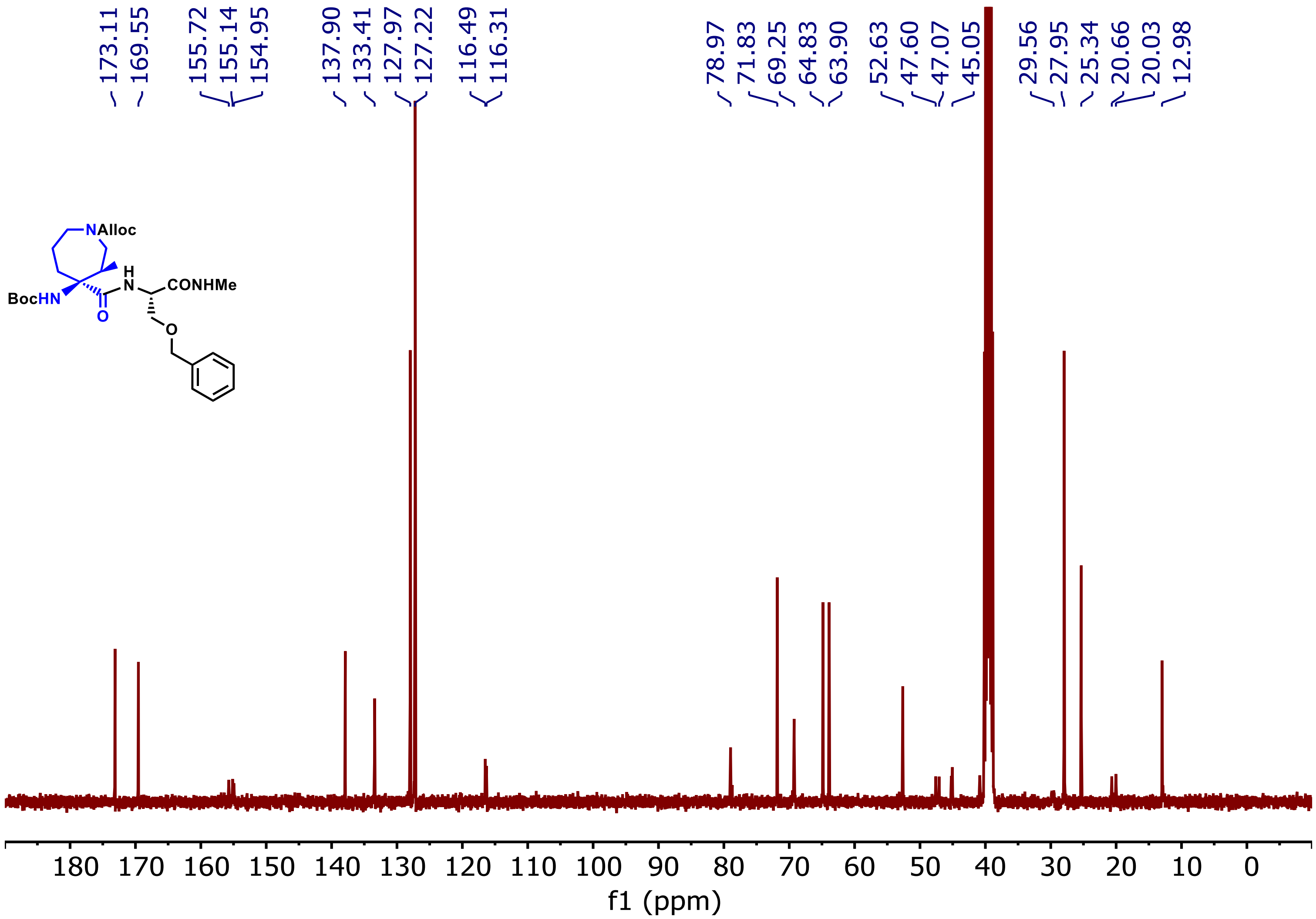

COSY compound 7

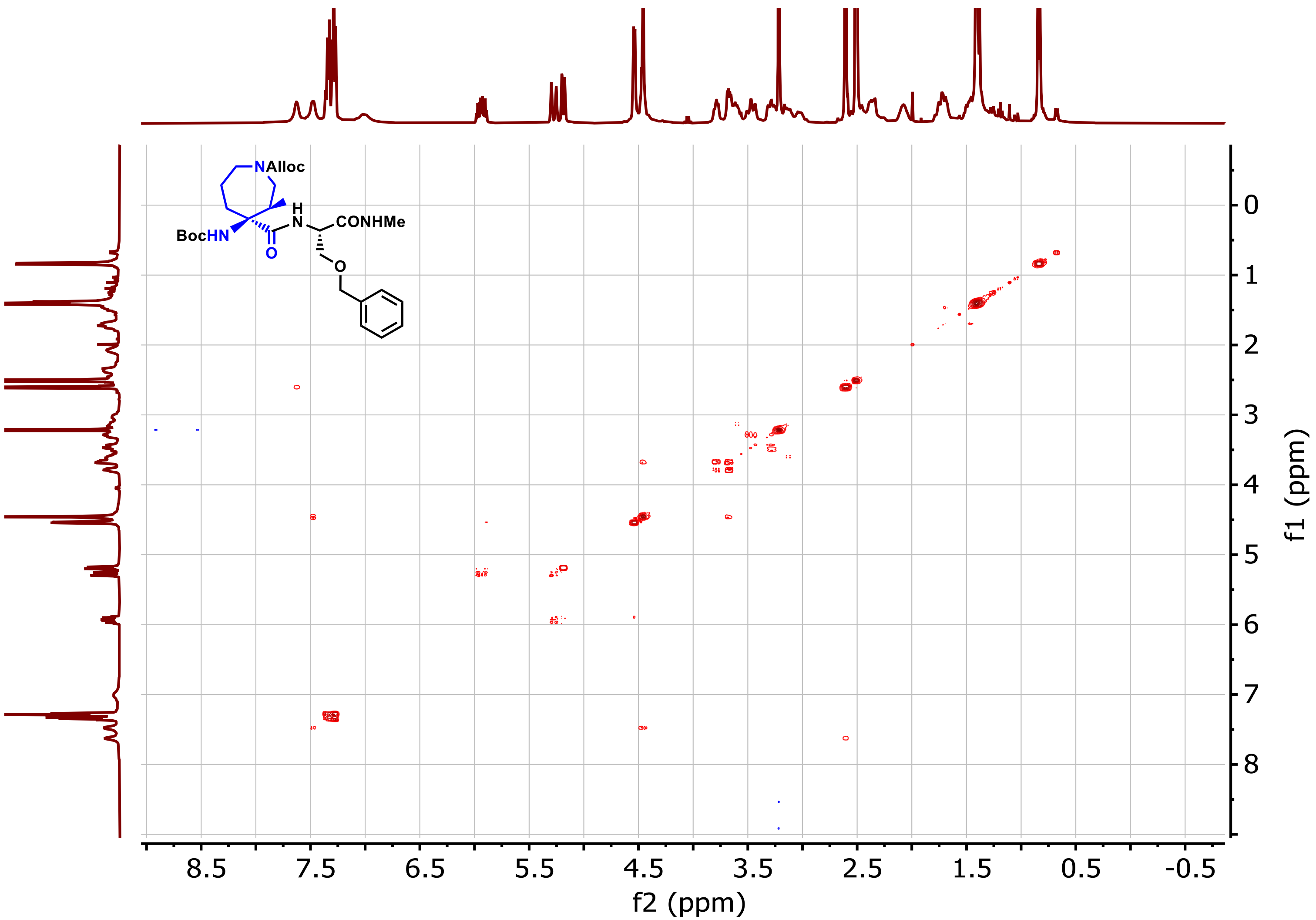

HSQC compound 7

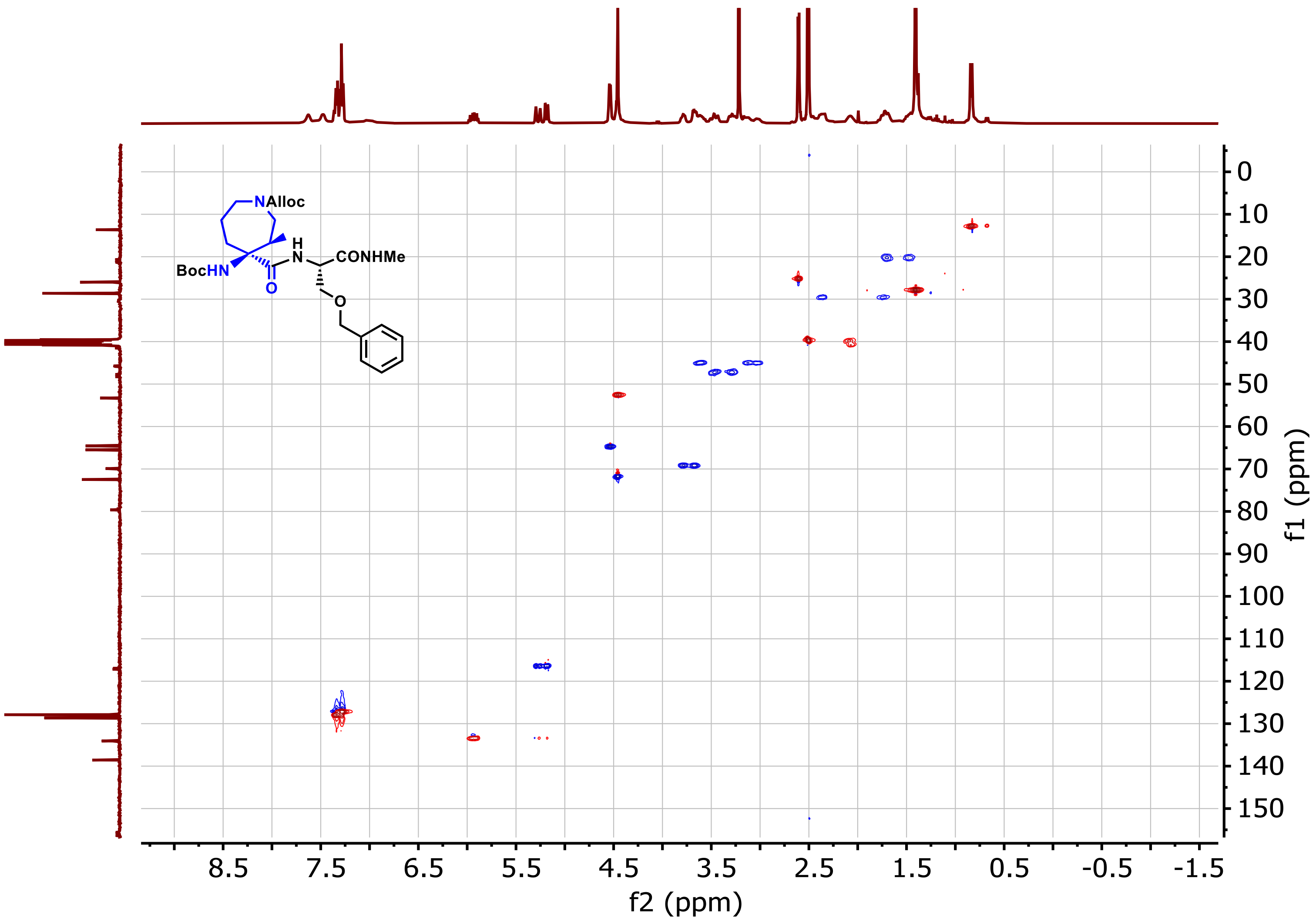

HMBC compound 7

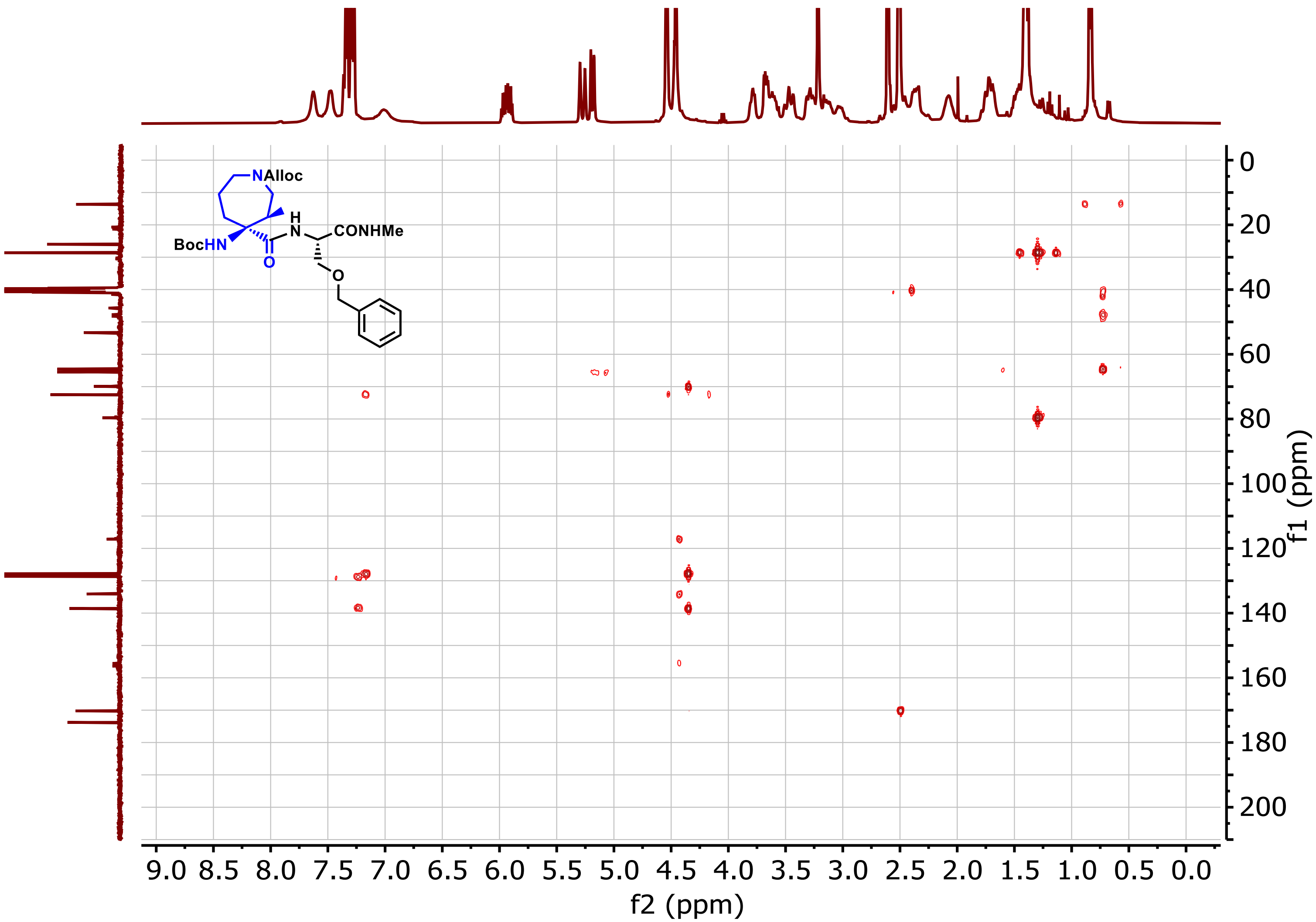

3.8. Synthesis of Boc-Aze(Alloc)-Lys(Z)-NHMe (8).

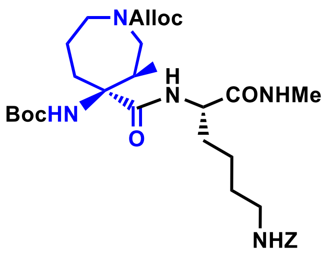

A solution of the azepane-derived amino acid **1** (0.070 g, 0.20 mmol) in dry DMF (2 mL) was treated with (1-[bis(dimethylamino)methylene]-1H-1,2,3-triazolo[4,5-b]pyridinium 3-oxide hexafluorophosphate (HATU, 0.089 g, 0.24 mmol), H-Lys(Z)-NHMe·TFA (0.087 g, 0.30 mmol) and *N*,*N*-diisopropylethylamine (0.092 mL, 0.53 mmol). After being stirred at 40 °C under microwave irradiation in a sealed vessel for 2 h, the solvent was removed under vacuum. The residue was dissolved in EtOAc and washed successively with 10% aq. soln. citric acid (2x), 10% aq. soln. $NaHCO_3$ (2x), H_2O (1x) and brine (1x). The organic phase was dried over Na_2SO_4 and evaporated to dryness. The residue was purified on a silica gel column, using a gradient from 100:1 to 10:1 of MeOH:CH_2Cl_2 as solvent, yielding **8** as a white amorphous solid (0.022 g, 17%).

HPLC: t_R = 8.34 min (gradient from 15% to 95% of CH_3CN-0.1% formic acid in H_2O-0.1% formic acid over 10 min).

^{1}H NMR (400 MHz, DMSO-*d*$_6$, 50 °C): δ 7.63 (bs, 1H, N*H*Me), 7.52 (bs, 1H, α-NH, Lys), 7.35 (m, 5H, Ph, Bn), 7.07 (s, 1H, NHZ), 6.72 (bs, 1H, 4-NH), 5.93 (ddt, 1H, *J* = 17.0, 10.5 and 5.0, 2’-H, Alloc), 5.26 (dq, 1H, *J* = 17.0 and 1.5, 3’-H, Alloc), 5.17 (dq, 1H, *J* = 10.5 and 1.5, 3’-H, Alloc), 5.00 (s, 2H, CH_2, Z), 4.53 (d, 3H, *J* = 5.0, 1’-H, Alloc), 4.15 (q, 1H, *J* = 4.5, α-CH, Lys), 3.54 (m, 1H, 7-H), 3.49 (m, 1H, 2-H), 3.33 (m, 1H, 2-H), 3.11 (m, 1H, 7-H), 2.94 (d, 2H, *J* = 7.0, NCH_2, Lys), 2.57 (d, 3H, *J* = 4.5, NCH_3), 2.29 (m, 1H, 5-H), 2.19 (m, 1H, 3-H), 1.72 (m, 2H, CH_2, Lys), 1.72 (m, 1H, 5-H), 1.65 (m, 1H, 6-H), 1.49 (m, 1H, 6-H), 1.43 (m, 2H, CH_2, Lys), 1.41 (s, 9H, CH_3, Boc), 1.26 (m, 2H, CH_2, Lys), 0.83 (d, 3H, *J* = 7.0, 3-CH_3).*

^{13}C{^{1}H} NMR (100 MHz, DMSO-*d*$_6$, 50 °C, two rotamers, Mr/mr = 1.1:1): δ 173.3 and 171.7 (CONH), 156.0, 155.9 and 155.3 (CO; Alloc, Boc and Z), 137.2 (1’-C, Ph, Z), 133.5 (2’-C, Alloc), 128.1, 127.5 and 127.4 (Ph, Z), 116.5 (3’-C, Alloc), 78.7 (C, Boc), 64.9 (CH_2, Z), 64.8 (1’-C, Alloc), 63.8 (4-C), 69.3 (β-C, Ser), 52.9 (α-C, Lys), 47.4 (2-C), 45.5 (7-C, mr), 45.3 (7-C, Mr), 40.1 (α-CH, Lys), 39.8 (3-C), 30.9 (CH_2, Lys), 30.4 (5-C, Mr), 30.1 (5-C, mr), 28.9 (CH_2, Lys), 28.0 (CH_3, Boc), 25.2 (NCH_3), 22.8 (CH_2, Lys), 20.7 (6-C, Mr), 20.1 (6-C, mr), 13.0 (3-CH_3).*

MS (ES+): *m*/*z* = 632.50 $[M+H]^+$.

Elemental analysis: calcd (%) for $C_{32}H_{49}N_5O_8$: C 60.84, H 7.82, N 11.09. Found (%): C 60.88, H 7.81, N 11.08.

***** 2D methods used for NMR peak assignments: COSY, TOCSY and HSQC (spectra provided)

^{1}H NMR (400 MHz, DMSO-d_6, 50 °C) compound 8

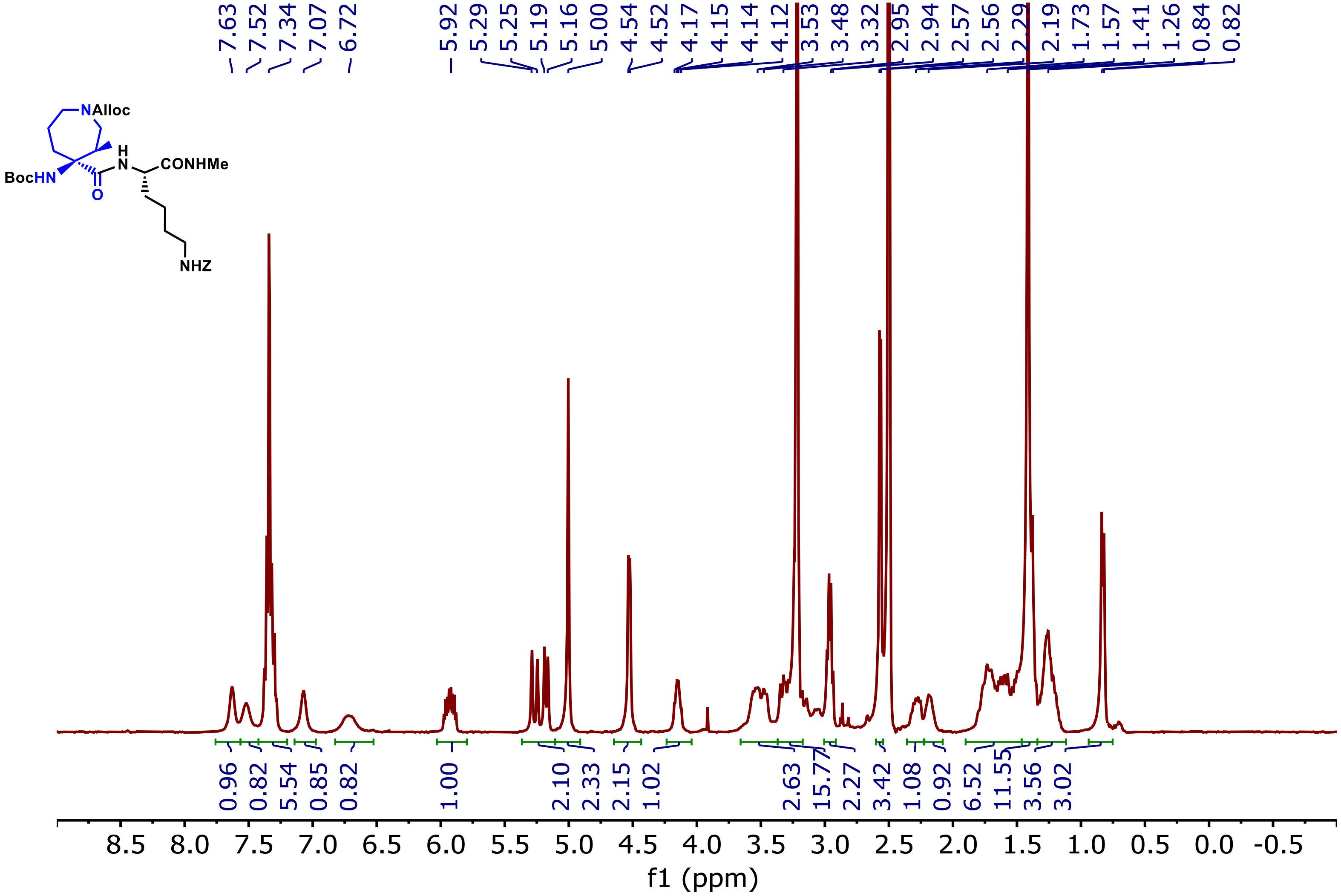

$^{13}C\{^{1}H\}$ NMR (100 MHz, DMSO-d_6, 50 °C) compound 8

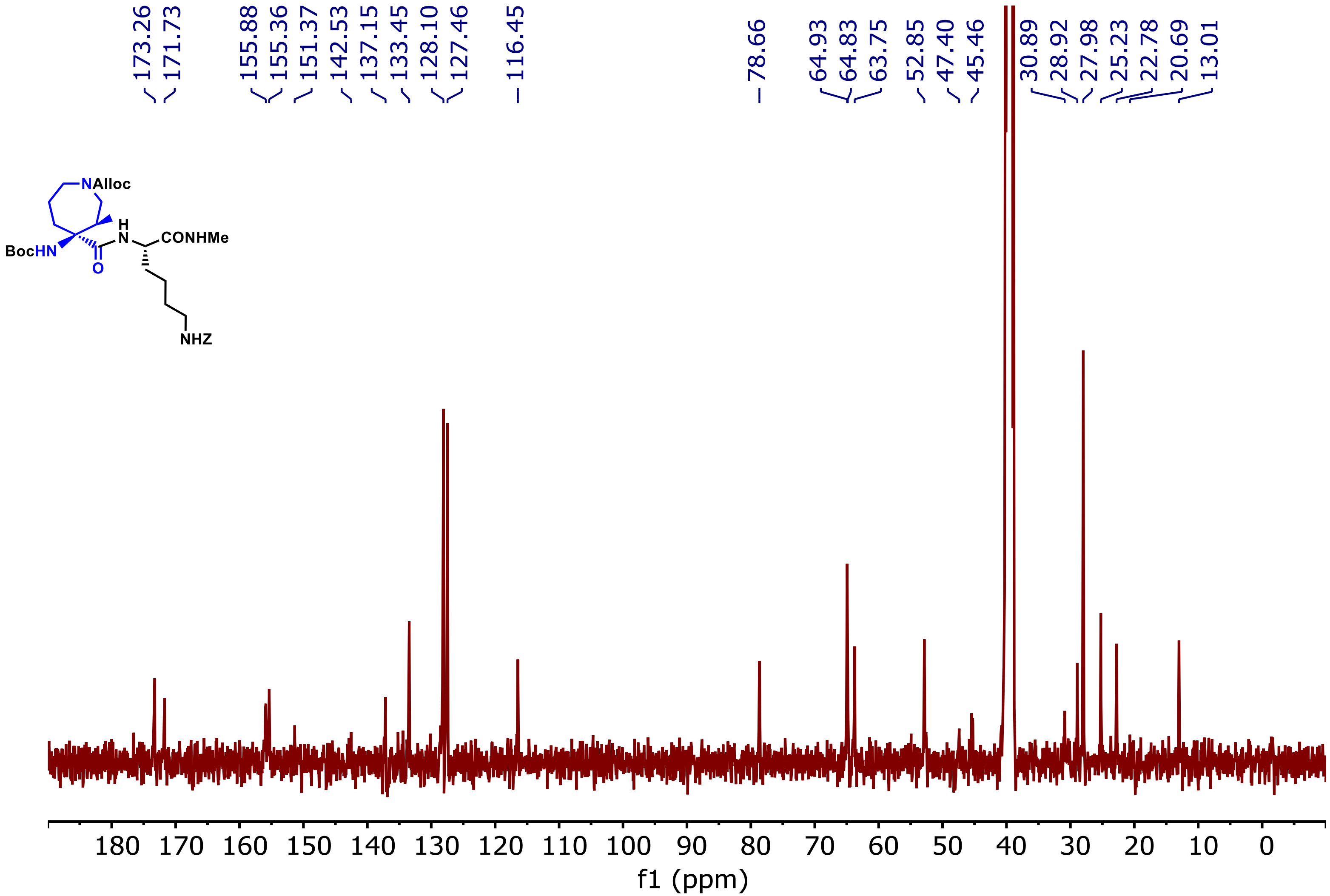

COSY compound 8

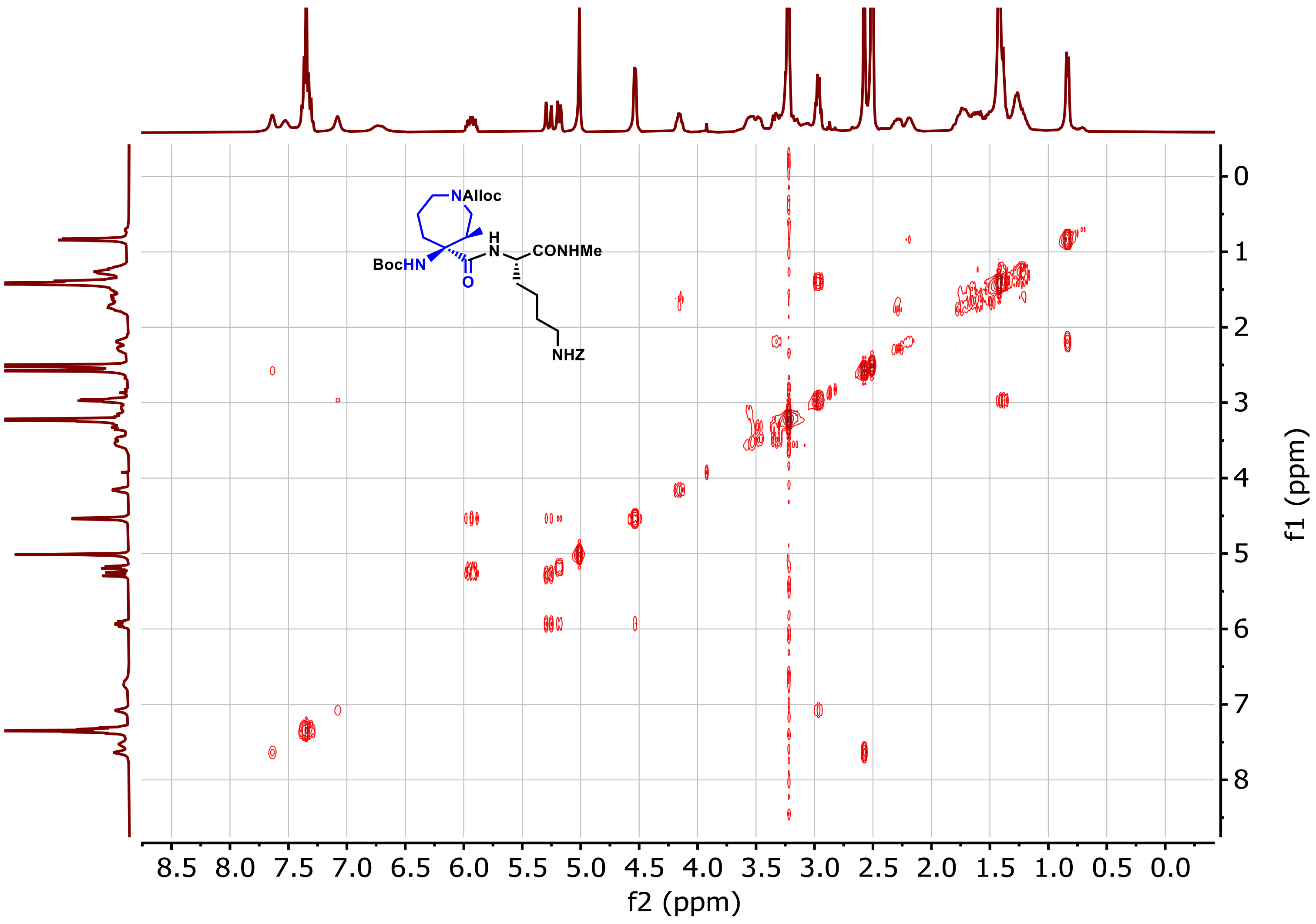

TOCSY compound 8

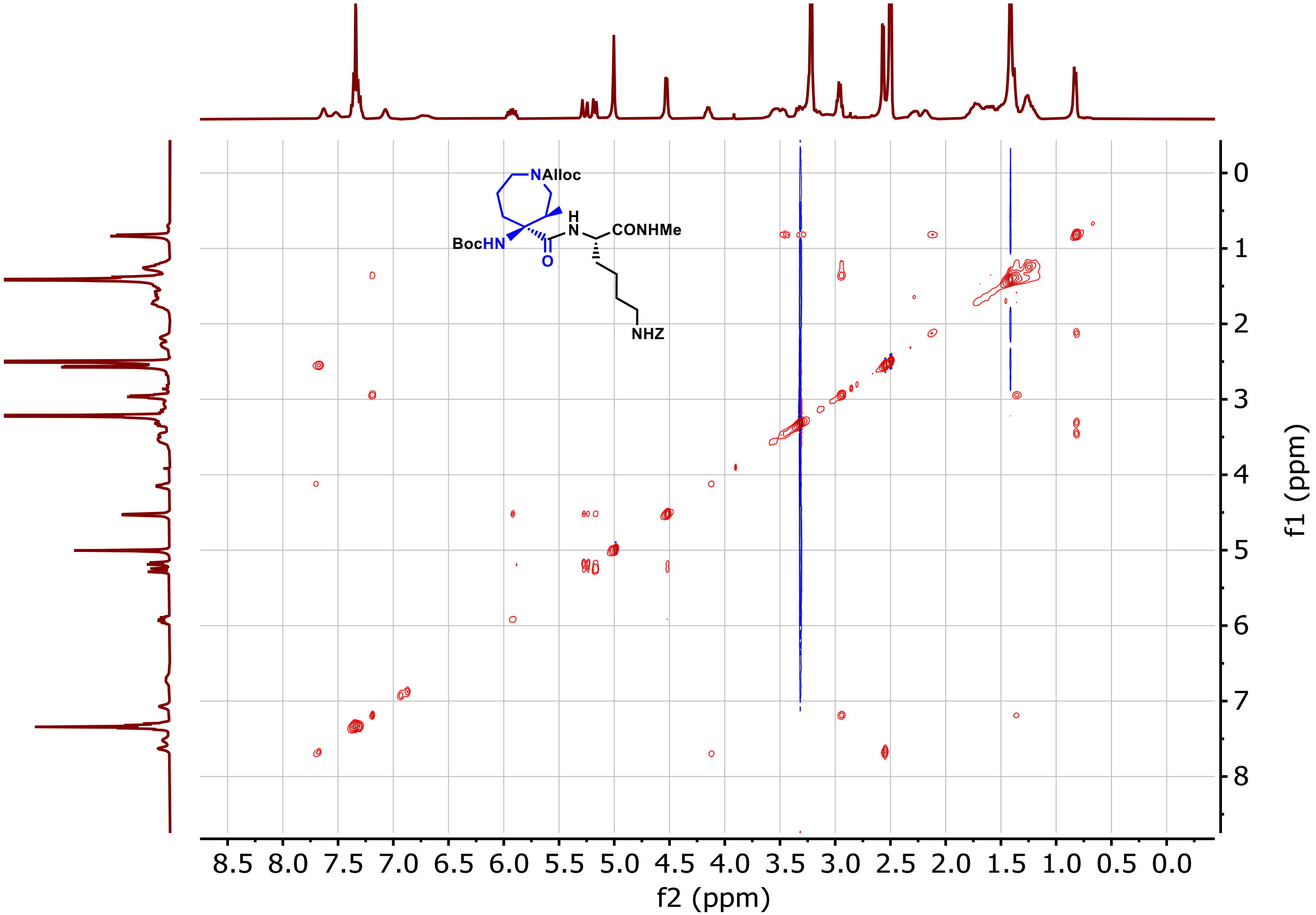

HSQC compound 8

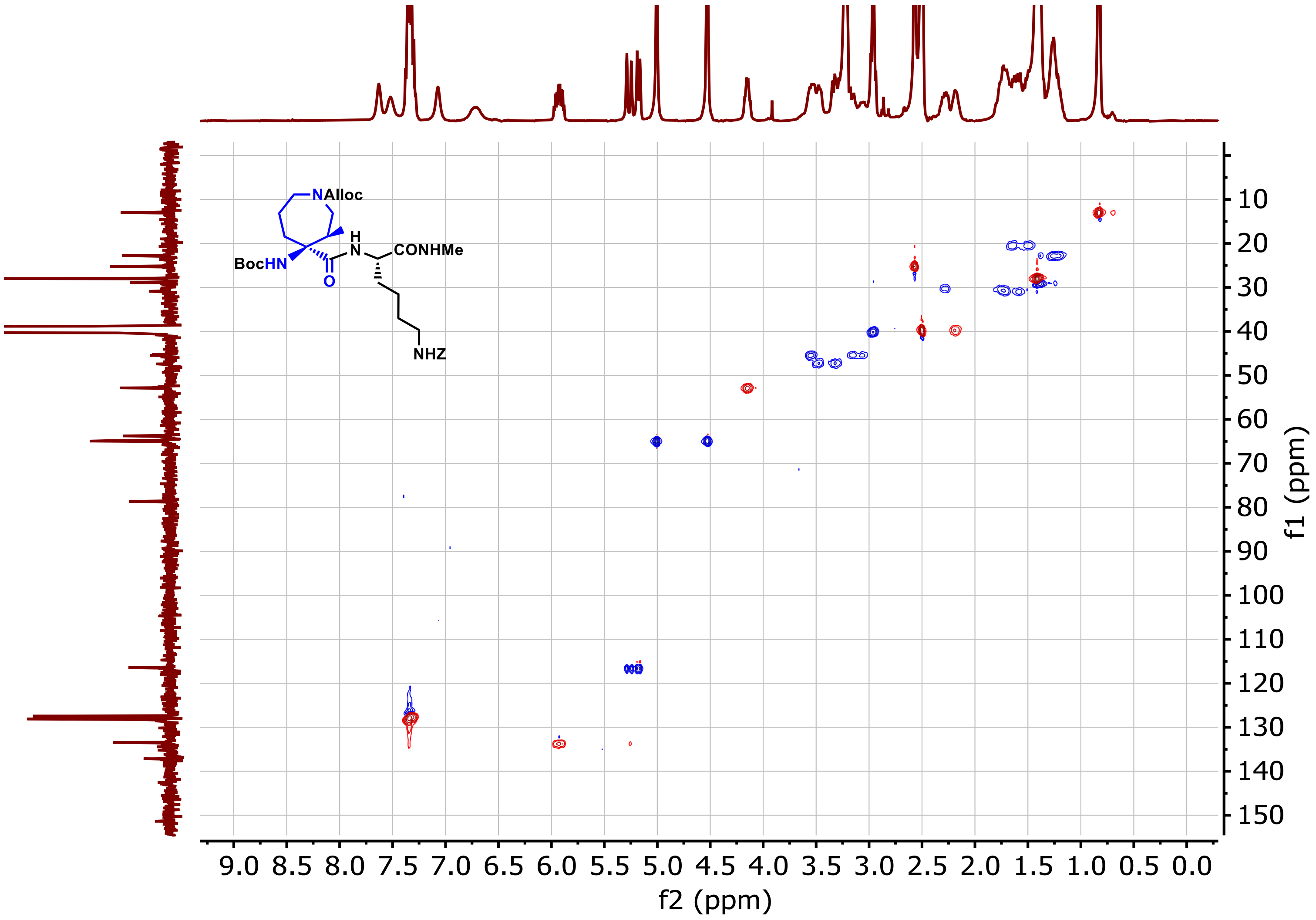

3.9. Synthesis of Boc-Aze(Alloc)-NHMe (9)

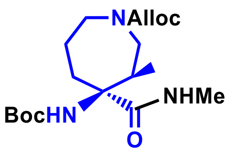

A solution of the azepane-derived amino acid **1** (0.044 g, 0.12 mmol) in dry CH_2Cl_2 (8 mL) was treated with benzotriazol-1-yloxy)tris(dimethylamino)phosphonium hexafluorophosphate (BOP, 0.066 g, 0.15 mmol), $MeNH_2$·HCl (0.083 g, 1.23 mmol) and triethylamine (0.192 mL, 1.38 mmol). After stirring at room temperature for 15 h, the solvent was evaporated to dryness. The residue was dissolved in EtOAc and washed successively with 10% aq. soln. citric acid (2x), 10% aq. soln. $NaHCO_3$ (2x), H_2O (1x) and brine (1x). The organic phase was dried over Na_2SO_4 and evaporated to dryness. The residue was purified on a silica gel column, using EtOAc:hexane (1:2) as solvent, yielding **9** as a white amorphous solid (0.035 g, 77%). *Caution!* The use of BOP as coupling reagent is discouraged because of the formation of carcinogenic HMPA, being PyBOP a convenient alternative.

HPLC: t_R = 10.62 min (gradient from 5% to 80% of CH_3CN-0.05% TFA in H_2O-0.05% TFA over 20 min).

^{1}H NMR (300 MHz, $CDCl_3$, two rotamers, Mr/mr = 1.1:1): δ 6.51 (s, 1H, N*H*CH_3, mr), 6.42 (s, 1H, N*H*CH_3, Mr), 5.94 (ddt, 1H, *J* = 16.5, 10.5 and 5.0, 2'-H, Alloc), 5.29 (dq, 1H, *J* = 16.5 and 1.5, 3'-H, Alloc), 5.20 (dq, 1H, *J* = 10.5 and 1.5, 3'-H, Alloc), 4.59 (d, 2H, *J* = 5.0, 1'-H, Alloc), 4.51 (s, 1H, 4-NH), 3.64 (m, 2H, 2-H, 7-H), 3.24 (m, 1H, 7-H), 3.05 (m, 1H, 2-H), 2.81 (d, 3H, *J* = 5.0, NCH_3), 2.61 (m, 1H, 5-H), 2.52 (m, 1H, 3-H), 1.86 (m, 1H, 5-H), 1.83 (m, 1H, 6-H), 1.57 (m, 1H, 6-H), 1.46 (s, 9H, CH_3, Boc), 0.87 (d, 3H, *J* = 7.1, 3-CH_3, mr), 0.85 (d, 3H, *J* = 7.0, 3-CH_3, Mr).*

^{13}C{^{1}H} NMR (75 MHz, $CDCl_3$): δ 174.7 (CONH, mr), 174.5 (CONH, Mr), 156.5 and 155.5 (CO, Alloc and Boc, Mr), 156.1 and 155.6 (CO, Alloc and Boc, mr), 133.3 (2'-C, Alloc), 117.5 (3'-C, Alloc), 80.8 (C, Boc), 66.3 (1'-C, Alloc), 65.2 (4-C), 47.6 (2-C, mr), 47.0 (2-C, Mr), 46.3 (7-C, Mr), 46.2 (7-C, mr), 41.4 (3-C, mr), 41.3 (3-C, Mr), 31.9 (5-C, Mr), 31.2 (5-C, mr), 28.5 (CH_3, Boc), 26.7 (NCH_3), 21.9 (6-C, Mr), 21.1 (6-C, mr), 14.4 (3-CH_3, Mr), 14.2 (3-CH_3, mr).*

MS (ES+): *m/z* = 370.2 $(M+H)^+$, 392.3 $(M+Na)^+$, 761.5 $(2M+Na)^+$.

Elemental analysis: calcd (%) for $C_{18}H_{31}N_3O_5$: C 58.52, H 8.46, N 11.37. Found (%): C 58.60, H 8.39, N 11.42.

***** 2D method used for NMR peak assignments: HSQC (spectrum provided)

^1H NMR (300 MHz, $CDCl_3$) compound 9

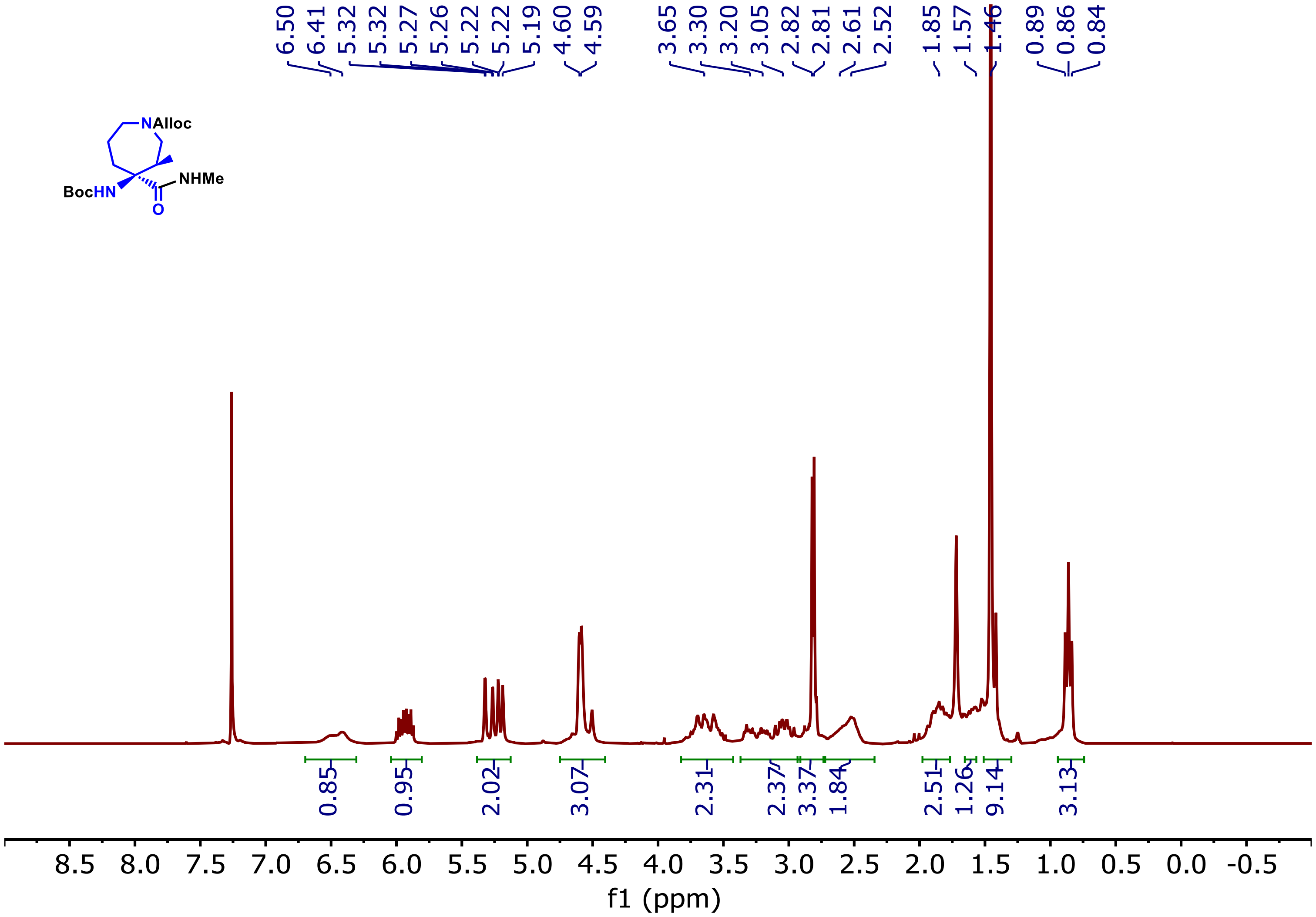

$^{13}C\{^1H\}$ NMR (75 MHz, $CDCl_3$) compound 9

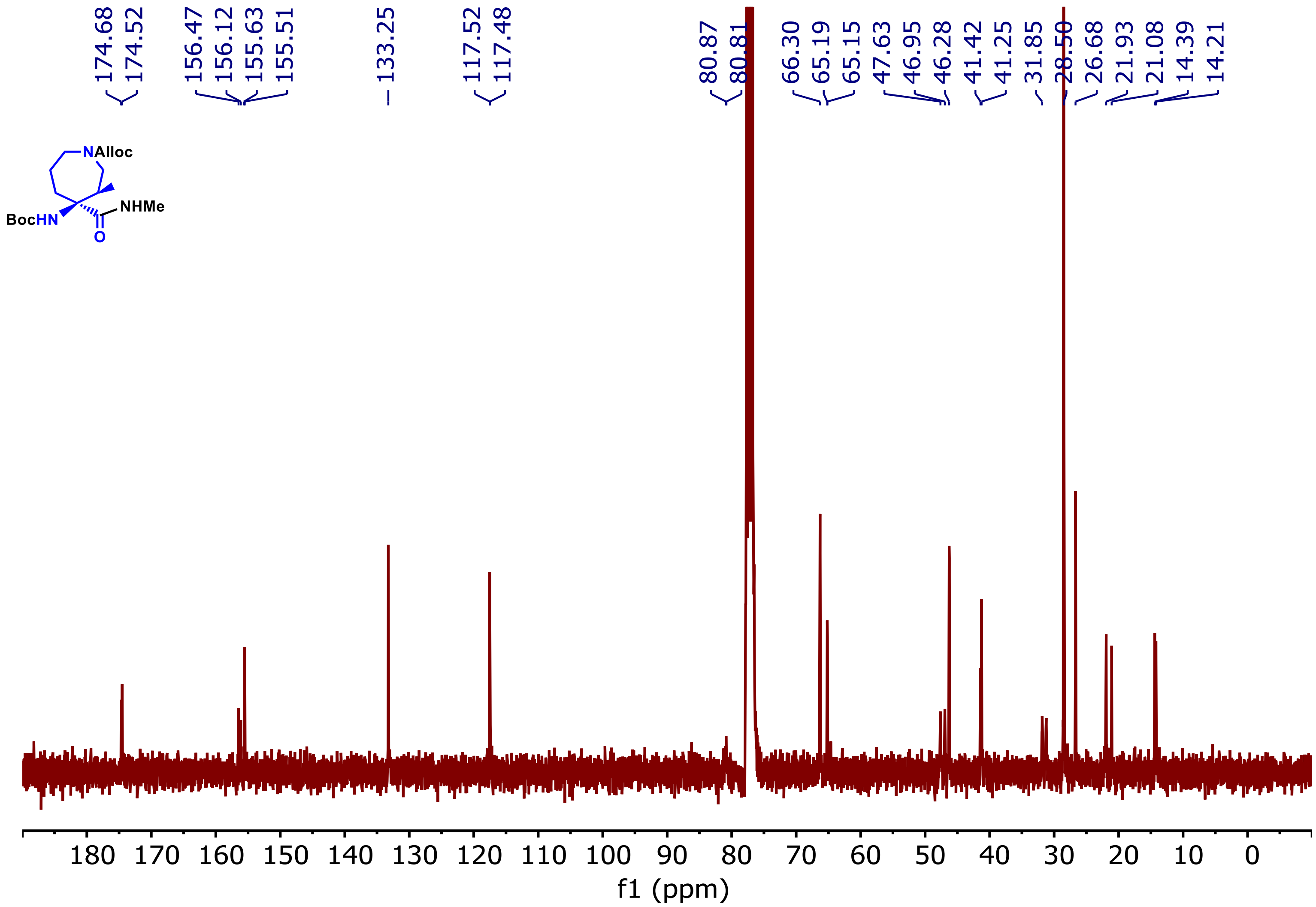

HSQC compound 9

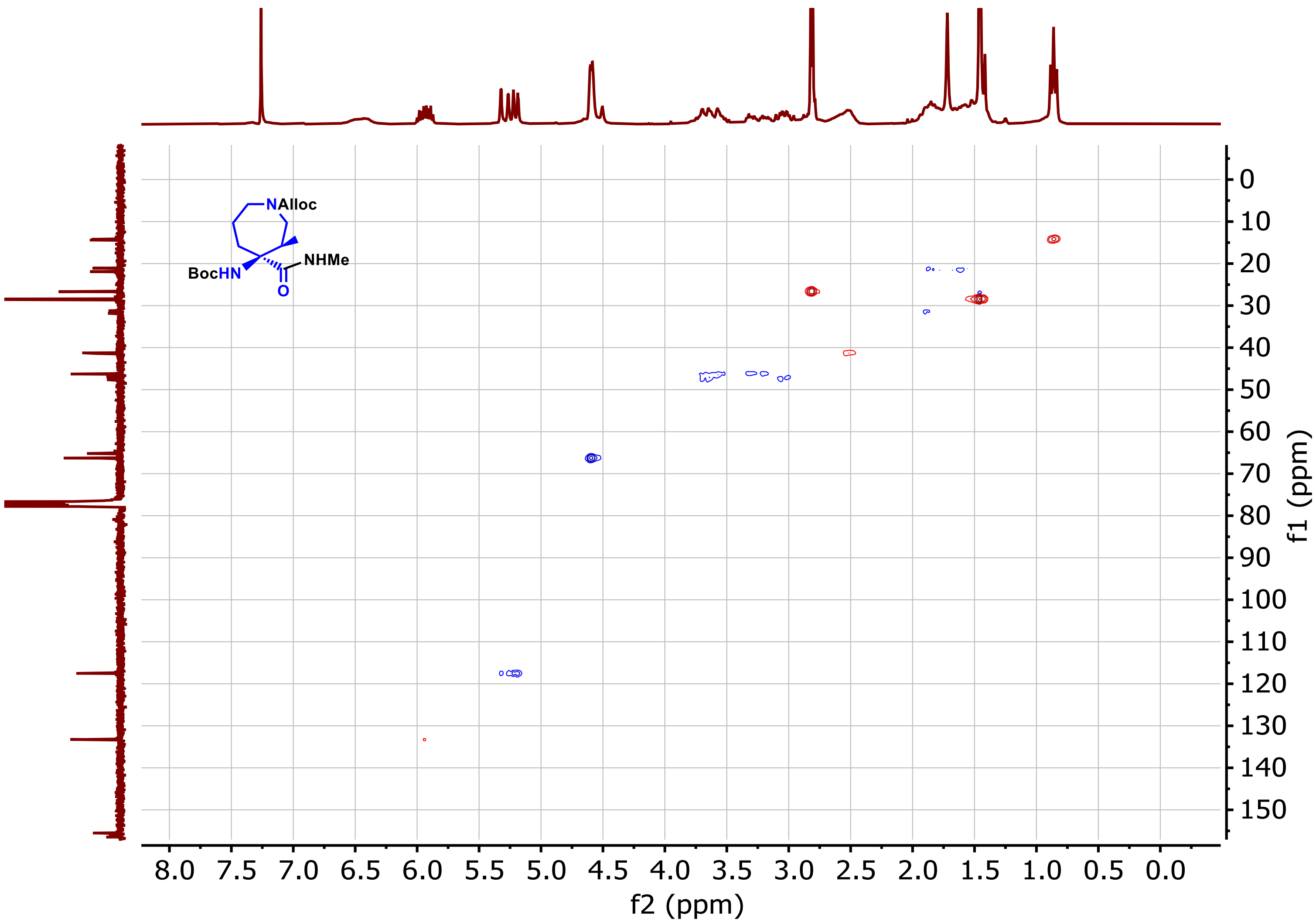

3.10. Synthesis of Boc-Ala-Aze(Alloc)-NHMe (10).

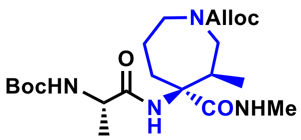

A solution of HCl/EtOAc (3 mL, 3.2 M) was added to a solution of azepane derivative **9** (0.029 g, 0.08 mmol) in EtOAc (2 mL) and stirred at room temperature for 4 h. Then, the solvent was evaporated under vacuum. The obtained residue was dissolved in dry THF (6 mL) and treated with benzotriazol-1-yloxy)tris(dimethylamino)phosphonium hexafluorophosphate (BOP, 0.059 g, 0.13 mmol), Boc-Ala-OH (0.025 g, 0.13 mmol) and triethylamine (0.030 mL, 0.22 mmol). After stirring at 65 °C for 48 h using an oil bath, the solvent was evaporated to dryness. The residue was dissolved in EtOAc and washed successively with 10% aq. soln. citric acid (2x), 10% aq. soln. $NaHCO_3$ (2x), H_2O (1x) and brine (1x). The organic phase was dried over Na_2SO_4 and evaporated to dryness. The residue was purified on a silica gel column, using EtOAc:hexane (6:1) as solvent, yielding **10** as an oil (0.018 g, 53%). *Caution!* The use of BOP as coupling reagent is discouraged because of the formation of carcinogenic HMPA, being PyBOP a convenient alternative.

HPLC: t_R = 11.14 min (gradient from 5% to 80% of CH_3CN-0.05% TFA in H_2O-0.05% TFA over 20 min).

^{1}H NMR (400 MHz, DMSO-*d*$_6$, two rotamers, Mr/mr = 1.1:1)*:* δ 7.29 (q, 1H, *J* = 4.5, N*H*CH_3), 7.26 (d, 1H, *J* = 7.0, α-NH, Ala), 7.04 (s, 1H, 4-NH), 5.92 (ddt, 1H, *J* = 17.0, 10.5 and 5.0, 2'-H, Alloc), 5.29 (dq, 1H, *J* = 17.0 and 1.5, 3'-H, Alloc), 5.18 (dq, 1H, *J* = 10.5 and 1.5, 3'-H, Alloc), 4.59 (m, 2H, 1'-H, Alloc), 3.98 (q, 1H, *J* = 7.0, α-H, Ala), 3.56 (m, 2H, 2-H, 7-H), 3.04 (m, 2H, 2-H, 7-H), 2.98 (td, 1H, *J* = 13.0 and 6.0, 7-H, mr), 2.55 (d, 3H, *J* = 4.5, NCH_3), 2.45 (m, 1H, 5-H), 2.11 (m, 1H, 3-H), 1.67 (m, 1H, 5-H), 1.63 (m, 1H, 6-H), 1.42 (m, 1H, 6-H), 1.39 (s, 9H, CH_3, Boc), 1.20 (d, 3H, *J* = 7.0, α-CH_3, Ala), 0.76 (d, 3H, *J* = 7.0, 3-CH_3, mr), 0.75 (d, 3H, *J* = 7.0, 3-CH_3, Mr).*

^{13}C{^{1}H} NMR (75 MHz, DMSO-*d*$_6$): δ 173.1 and 173.0 (CONH, Mr), 173.1 and 172.9 (CONH, mr), 156.3 and 155.3 (CO, Alloc and Boc, Mr), 156.3 and 155.1 (CO, Alloc and Boc, mr), 133.6 (2'-C, Alloc), 116.7 (3'-C, Alloc, Mr), 116.5 (3'-C, Alloc, mr), 78.8 (C, Boc), 65.1 (1'-C, Alloc), 63.9 (4-C), 50.8 (α-CH, Ala), 47.7 (2-C, Mr), 47.2 (2-C, mr), 45.6 (7-C, mr), 45.4 (7-C, Mr), 41.2 (3-C), 30.3 (5-C, Mr), 30.0 (5-C, mr), 28.2 (CH_3, Boc), 25.9 (NCH_3), 20.9 (6-C, Mr), 20.3 (6-C, mr), 16.4 (α-CH_3, Ala), 13.3 (3-CH_3, mr), 13.2 (3-CH_3, Mr).*

MS (ES+): *m*/*z* = 441.2 $(M+H)^+$, 463.3 $(M+Na)^+$, 903.5 $(2M+Na)^+$.

Elemental analysis: calcd (%) for $C_{21}H_{36}N_4O_6$: C 57.25, H 8.24, N 12.72. Found (%): C 57.18, H 8.20, N 12.78.

***** 2D methods used for NMR peak assignments: COSY and HSQC (spectra provided)

^{1}H NMR (400 MHz, DMSO-d_6) compound 10

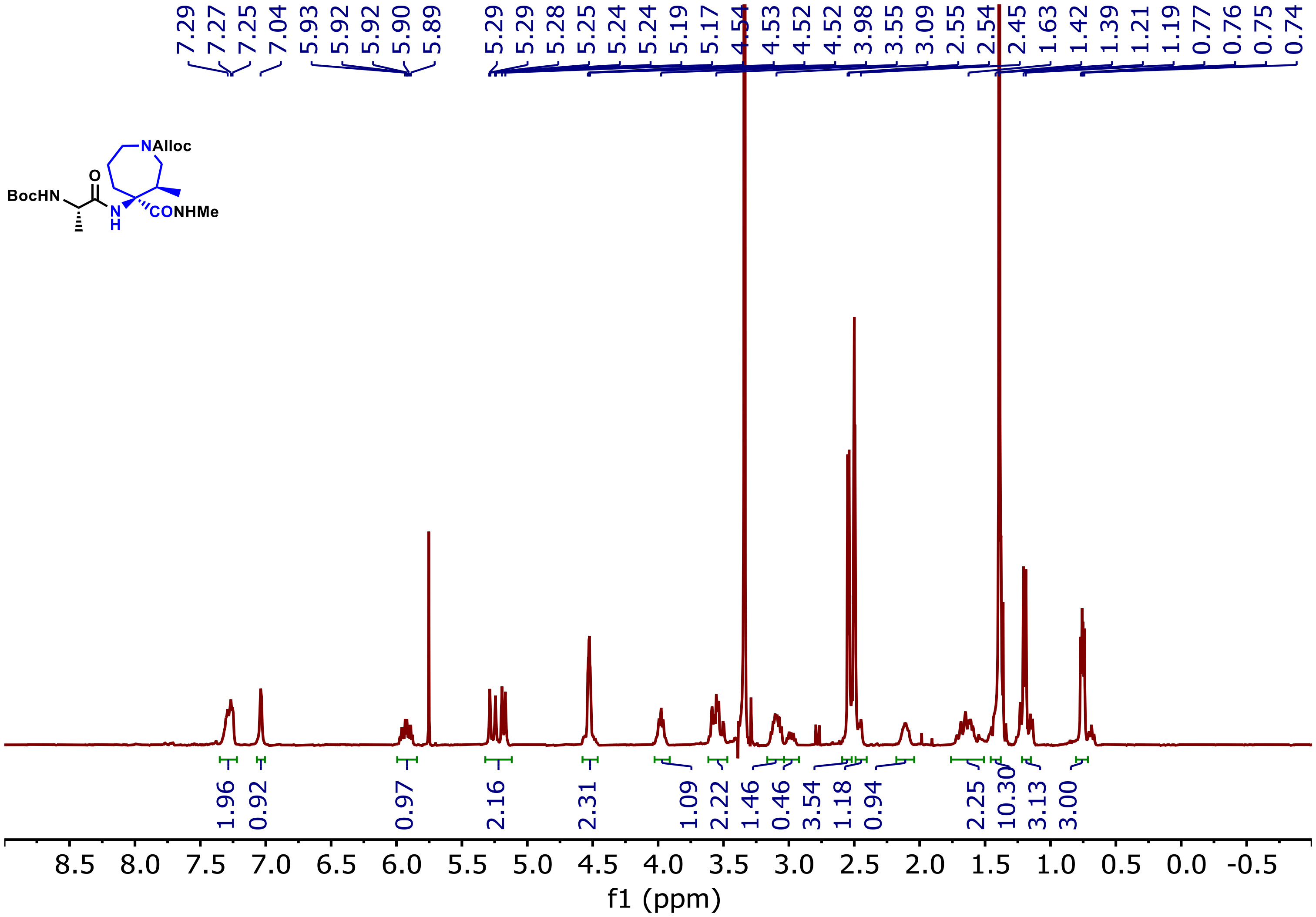

$^{13}C\{^{1}H\}$ NMR (75 MHz, DMSO-d_6) compound 10

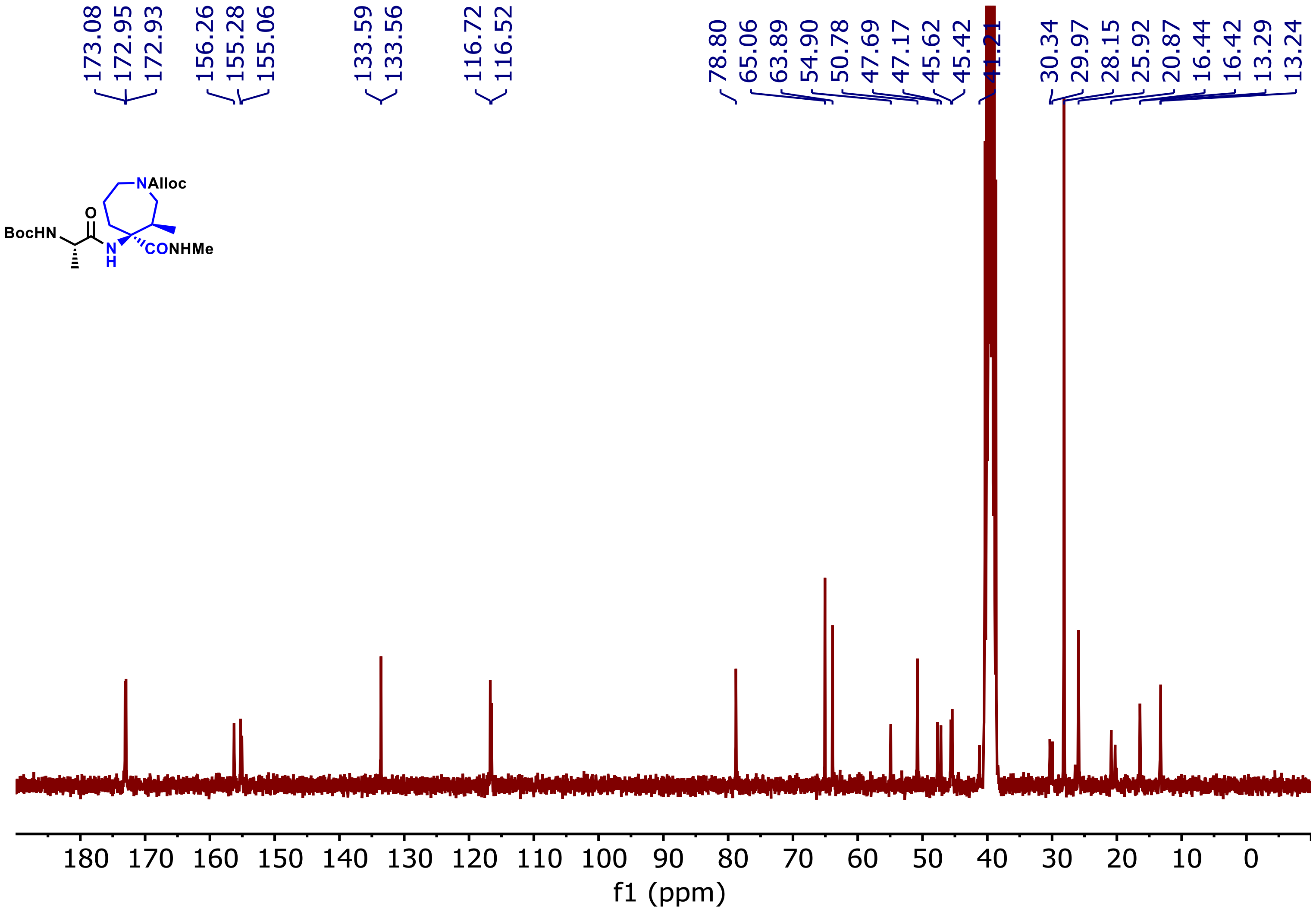

COSY compound 10

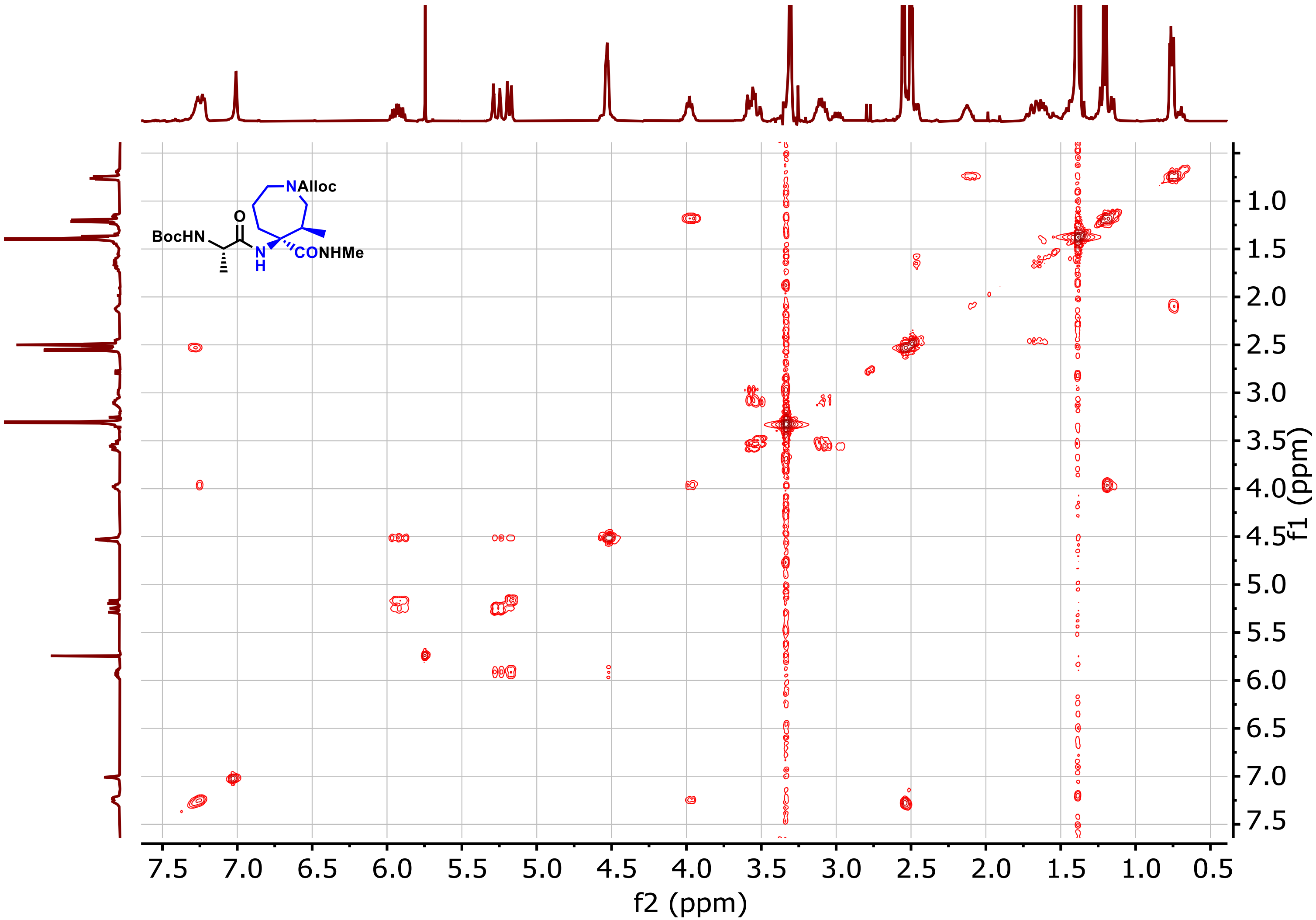

HSQC compound 10

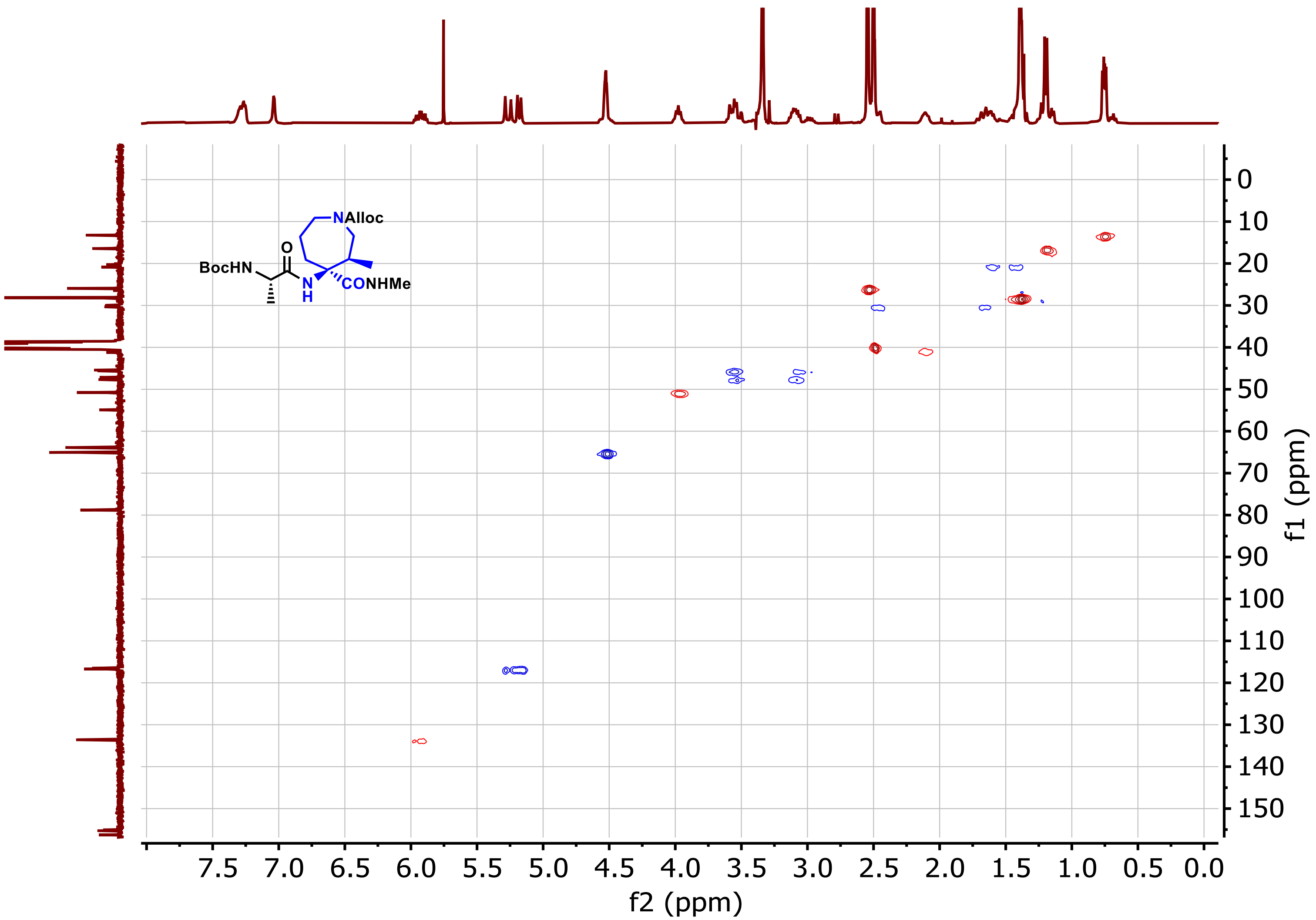

4. Variable temperature NMR experiments

Samples of compounds **2**-**8** and **10** at 7-10 mM concentration in DMSO-d_6 were prepared and transferred to an NMR tube. ^{1}H-NMR spectra were acquired at different temperatures (25-85 °C, 10 °C steps) in a Bruker Avance III HD 400 Mhz. The variation of the amide proton chemical shifts (Δδ, ppb) was plotted against the variation of temperature (ΔT, K) and the data fitted to a straight line (equation and R^2 shown in the graphs). The slope of the equation represents the temperature coefficient (Δδ/ΔT, ppb·K^{-1}). The average temperature coefficients are reported when there is splitting in the NMR signals due to the existence of rotamers. In DMSO-d_6, it has been stablished that temperature coefficients below 3 ppb·K^{-1} (in absolute value) are indicative of intramolecular hydrogen bond in small peptides, while solvent exposed NHs typically display values over 4 ppb·K^{-1}.[S10,S11] Values in between 3 and 4 are not conclusive.

Figs S2-S9 show the amide proton region of the ^{1}H-NMR spectra at different temperatures for compounds **2**-**8** and **10**, the plots of Δδ against ΔT and the fitted lines, including the linear equation and R^2.

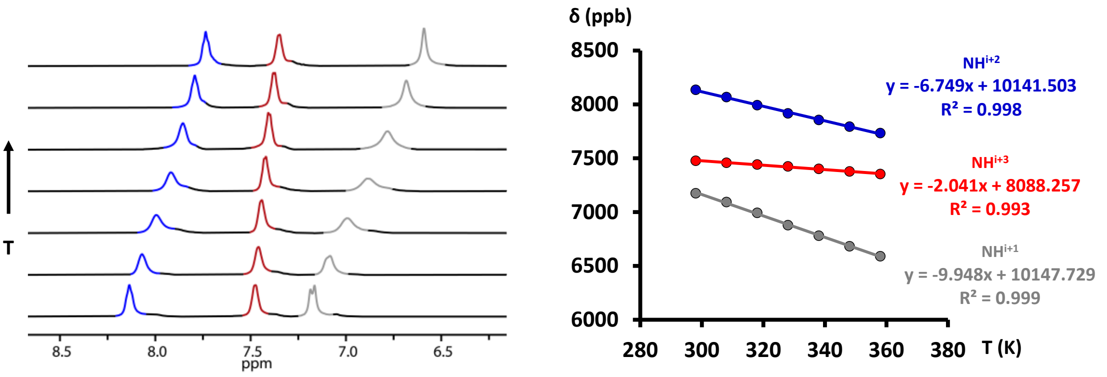

Fig S2. Amide proton region of the ^{1}H-NMR spectra at different temperatures for compound Boc-**Aze(Alloc)**-Gly-NHMe **2** (DMSO-d_6, 400 MHz) and plot of Δδ against ΔT for all the NH protons (NH^{i+1} in grey, NH^{i+2} in blue and NH^{i+3} in red).

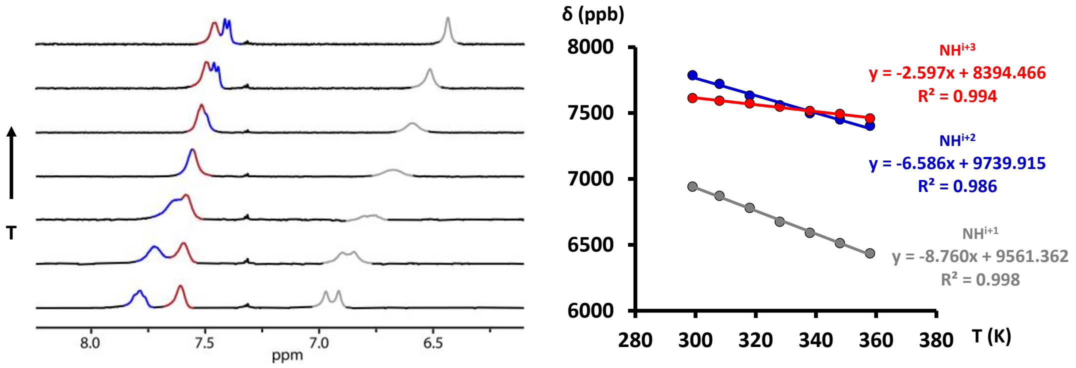

Fig S3. Amide proton region of the ^{1}H-NMR spectra at different temperatures for compound Boc-**Aze(Alloc)**-Ala-NHMe **3** (DMSO-d_6, 400 MHz) and plot of Δδ against ΔT for all the NH protons (NH^{i+1} in grey, NH^{i+2} in blue and NH^{i+3} in red).

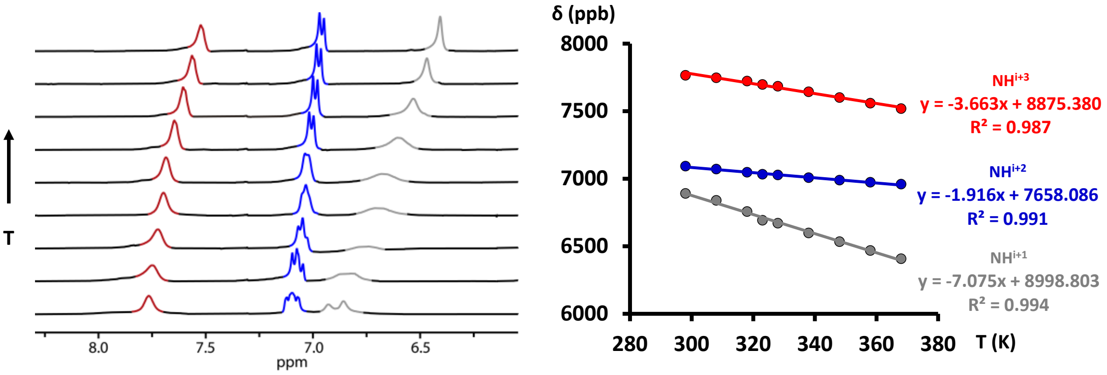

Fig S4. Amide proton region of the ^{1}H-NMR spectra at different temperatures for compound Boc-**Aze(Alloc)**-Val-NHMe **4** (DMSO-d_6, 400 MHz) and plot of Δδ against ΔT for all the NH protons (NH^{i+1} in grey, NH^{i+2} in blue and NH^{i+3} in red).

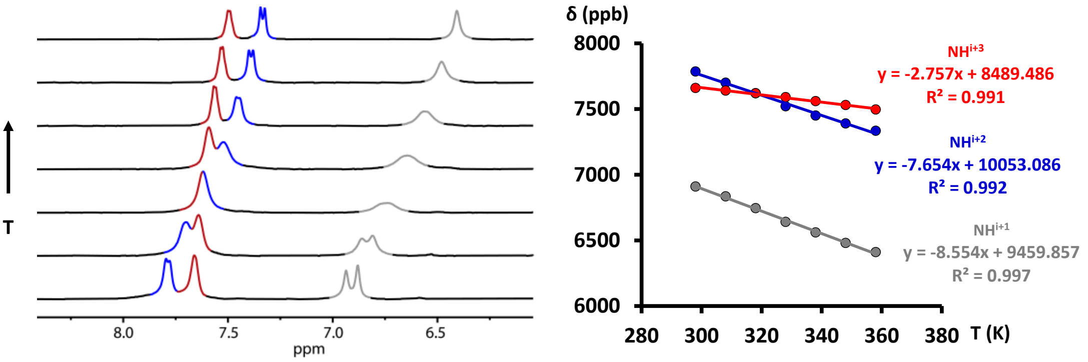

Fig S5. Amide proton region of the ^{1}H-NMR spectra at different temperatures for compound Boc-**Aze(Alloc)**-Leu-NHMe **5** (DMSO-d_6, 400 MHz) and plot of Δδ against ΔT for all the NH protons (NH^{i+1} in grey, NH^{i+2} in blue and NH^{i+3} in red).

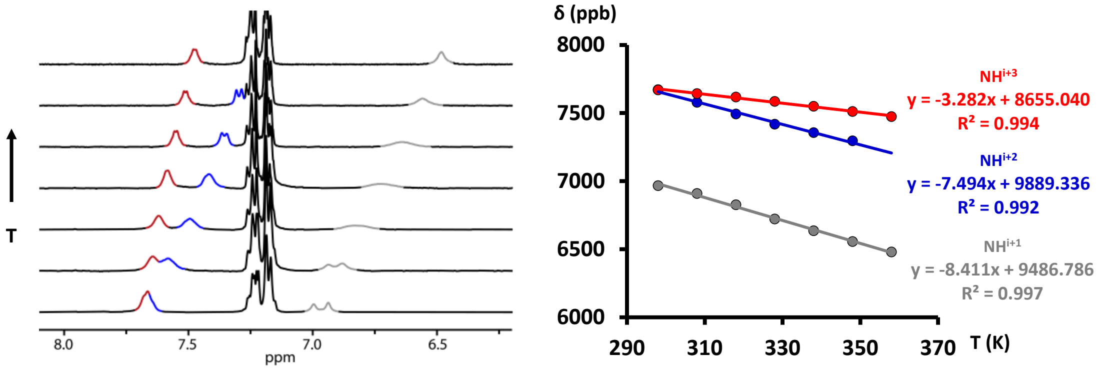

Fig S6. Amide proton region of the ^{1}H-NMR spectra at different temperatures for compound Boc-**Aze(Alloc)**-Phe-NHMe **6** (DMSO-d_6, 400 MHz) and plot of Δδ against ΔT for all the NH protons (NH^{i+1} in grey, NH^{i+2} in blue and NH^{i+3} in red).

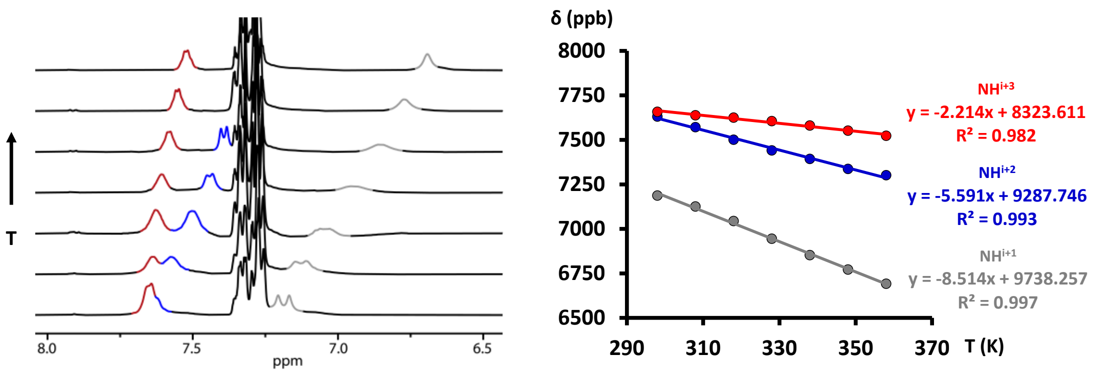

Fig S7. Amide proton region of the ^{1}H-NMR spectra at different temperatures for compound Boc-**Aze(Alloc)**-Ser(Bn)-NHMe **7** (DMSO-*d*$_6$, 400 MHz) and plot of Δδ against ΔT for all the NH protons (NH^{i+1} in grey, NH^{i+2} in blue and NH^{i+3} in red).

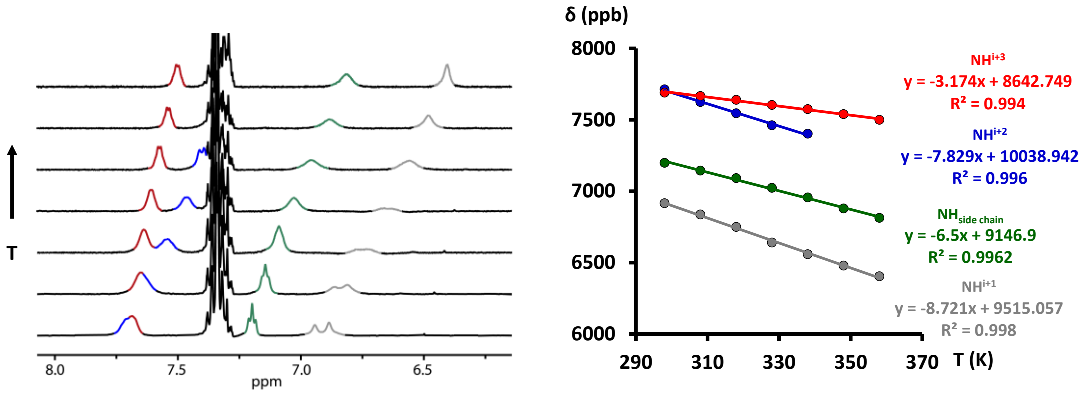

Fig S8. Amide proton region of the ^{1}H-NMR spectra at different temperatures for compound Boc-**Aze(Alloc)**-Lys(Z)-NHMe **8** (DMSO-*d*$_6$, 400 MHz) and plot of Δδ against ΔT for all the NH protons (NH^{i+1} in grey, NH^{i+2} in blue, NH^{i+3} in red and NH$_{\text{side chain}}$ in green).

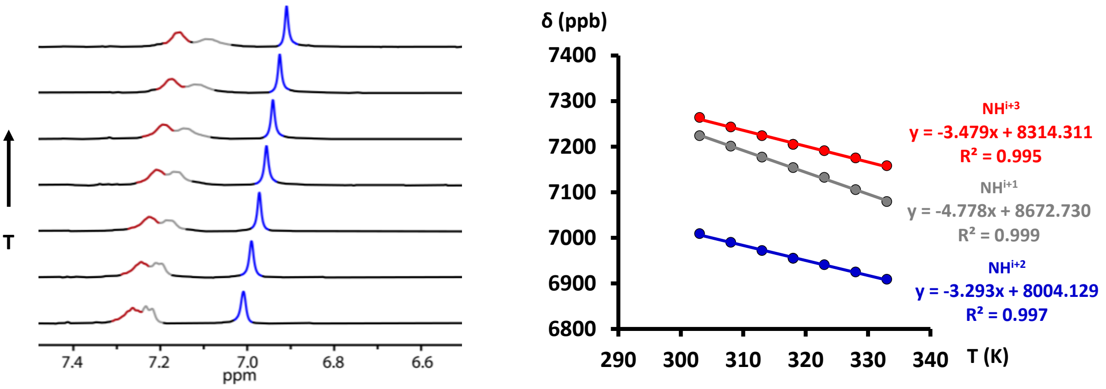

Fig S9. Amide proton region of the ^{1}H-NMR spectra at different temperatures for compound Boc-Ala-**Aze(Alloc)**-NHMe **10** (DMSO-*d*$_6$, 400 MHz) and plot of Δδ against ΔT for all the NH protons (NH^{i+1} in grey, NH^{i+2} in blue and NH^{i+3} in red).

5. X-Ray crystallography

5.1. X-ray structure of compound 2 [Boc-Aze(Alloc)-Gly-NHMe]

(a) Preparation of single crystals for X-ray diffraction analysis: pure compound **2** (1.5 mg) was dissolved in MeOH (1 mL) and the mixture was put in a crystallizing dish, resulting in spontaneous crystallization after 12 days at 4 ^{o}C in a closed jar. Crystal data on Table S3. X-ray diffraction was performed at BL13-XALOC beamline at ALBA Synchrotron with the collaboration of ALBA staff.

*(b) X-ray structure of compound **2** (single molecule):* Fig S10a shows the X-ray structure of a single molecule from the asymmetric unit, showing the existence of an intramolecular hydrogen bond between the NH of the *N*-methylamide moiety and the Boc carbonyl oxygen (distance O-N 3.04 Å, angle N-H···O 162.4^{o}). This intramolecular interaction is indicative of the existence of a type I β-turn (dihedral angles of the central residues ϕ_{i+1}= -59.2; ψ_{i+1}= -30.8; ϕ_{i+2}= -87.1; ψ_{i+2}= 4.3). Dihedral angle measurements for the central residues of the other four molecules of the asymmetric unit are presented in Table 2, main text.

(a) (b)

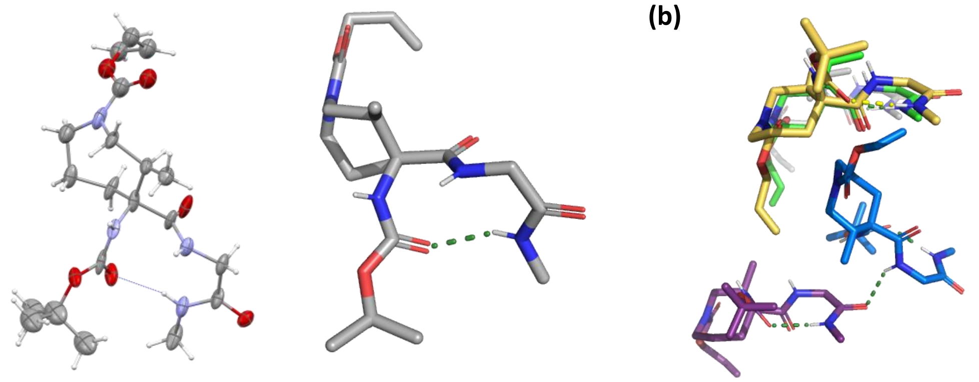

Fig S10. (a) X-ray structure of derivative **2** [Boc-Aze(Alloc)-Gly-NHMe], both in ORTEP (ledt) showing 30% probability displacement ellipsoids for non-H atoms and fixed-size spheres of radius 0.1 Å for hydrogen atoms, and stick (right) representation showing the intramolecular H-bond (for clarity only polar hydrogens are shown). (b) Representation of the asymmetric unit. ORTEP image generated with Mercury.[S12] Stick representations generated with PyMOL.[S13]

*(c) Asymmetric unit of compound **2***: Fig S10b shows the asymmetric unit for the obtained crystal structure, composed of five molecules displaying the type I β-turn intramolecular H-bond. There is one additional intermolecular H-bond in the asymmetric unit between de CO^{i+2} of one molecule and the NH^{i+2} of a different molecule (distance O-N 2.95 Å, angle N-H···O 161.4^{o}).

*(d) Packing of compound **2***: Crystal packing of compound **2** shows that the five independent molecules of the asymmetric unit form three independent H-bonded chains, two of them between two of the independent molecules and the third just with one of them. Fig. S11 show each independent molecule in a different color. The chain formed by molecules in grey form a 2_1 helical axis, while the other two chains are stacked by translation. There are intermolecular bifurcated H-bonds between the CO^{i+2} of one molecule and the NH^{i+1} and NH^{i+2} of different molecules. The NH^{i+3} is always involved in the β-turn intramolecular H-bond with the CO^{i} while the CO^{i+1} is not involved in any H-bonding probably because it is hindered by the nearby azepane ring.

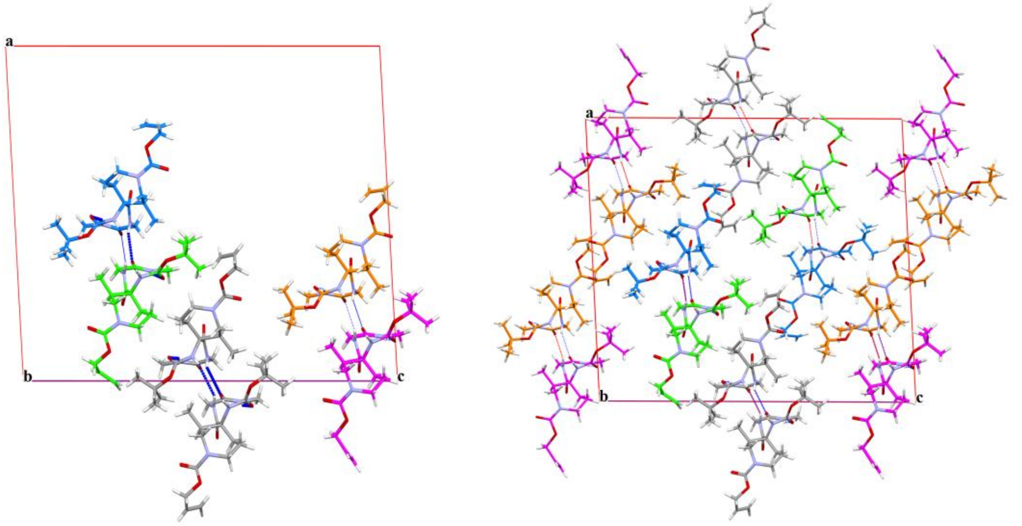

Fig S11: Molecular packing of compound **2** [Boc-Aze(Alloc)-Gly-NHMe]. Each molecule of the asymmetric unit is displayed in a different color. Image generated with Mercury.[S12]

5.2. X-ray structure of compound 3 [Boc-Aze(Alloc)-Ala- NHMe]

(a) Preparation of single crystals for X-ray diffraction analysis: pure compound **3** (5 mg) was dissolved in MeOH (5 mL) and the mixture was put in a crystallizing dish, resulting in spontaneous crystallization after 20 days at 4 ºC in a closed jar. Crystal data on Table S3. X-ray diffraction was performed in a Bruker MicroStar 2.7 kW, with a four-circle goniometer, with Kappa geometry and Bruker CCD detector, using CuKα radiation.

*(b) X-ray structure of compound **3** (single molecule):* Fig S12a shows the x-ray structure of one of the two independent molecules from the asymmetric unit, showing the existence of an intramolecular hydrogen bond between the NH of the *N*-methylamide moiety and the Boc carbonyl oxygen (distance O-N 2.923 Å, angle N-H···O 166.2º). This intramolecular interaction is indicative of the existence of a type I β-turn (dihedral angles of the central residues ϕ_{i+1}= -67.3; ψ_{i+1}= -8.7; ϕ_{i+2}= -106.6; ψ_{i+2}= -0.9). Dihedral angle measurements for the central residues of the other molecule of the asymmetric unit are presented in table 2, main text.

(a) **(b)**

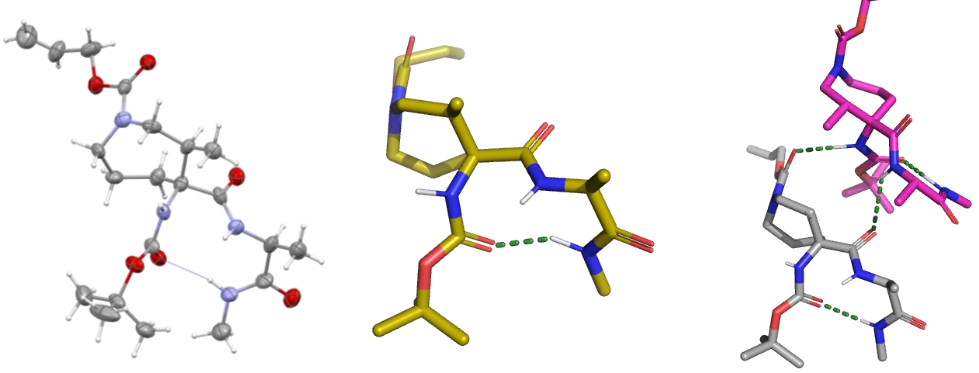

Fig S12. (a) X-ray structure of derivative **3** [Boc-Aze(Alloc)-Ala-NHMe], both in ORTEP (left) showing 30% probability displacement ellipsoids for non-H atoms and fixed-size spheres of radius 0.1 Å for hydrogen atoms, and in stick (stick) representation showing the intramolecular H-bond (for clarity only polar hydrogens are shown). (b) Representation of the asymmetric unit. ORTEP image generated with Mercury.[S11] Stick representations generated with PyMOL.[S13]

*(c) Asymmetric unit of compound **3***: Fig S12b shows the asymmetric unit for the obtained crystal structure, composed of two molecules forming a head to tail dimer stabilized with two intermolecular hydrogen bonds: one between the carbonyl of the Alloc group and the NH^{i+1} (distance O-N 2.98 Å, angle N-H···O=177.8°) and another between CO^{i+1} and NH^{i+2} (distance O-N 3.22 Å, angle N-H···O 138.6°).

*(d) Packing of compound **3**:* Crystal packing of derivative **3** shows that the crystals belong to the tetragonal system, with a quaternary axis. One of the independent molecules of the asymmetric unit (A in Fig. S13) forms an infinite right-handed helix, while the other independent molecule (B in Fig. S13) is bound to this helix arrangement via a series of intermolecular H-bonds. Thus, the structure is formed by four dimers stabilized by inter-dimer H-bonds. For dimer A1-B1, these H-bonds are:

- CO^{i+2} of molecule A1 - NH^{i+2} of molecule A2 (distance O-N 2.80 Å, angle N-H···O 152.0°).

- NH^{i+1} of molecule A1 - CO^{i+2} of molecule B4 (distance O-N 3.07 Å, angle N-H···O 176.5°).

Hydrophobic interactions are also observed in the stacking of the allyl groups as well as in the packing between adjacent helices.

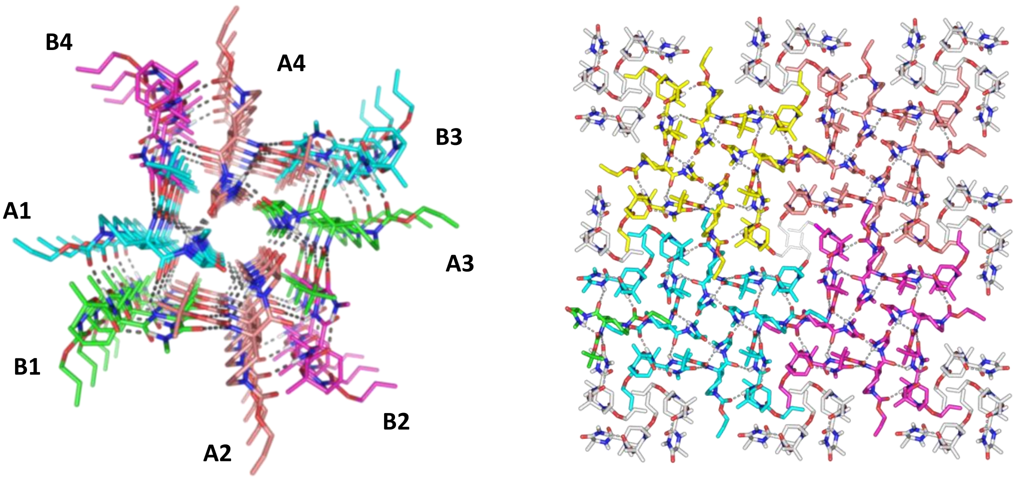

Fig S13: Two projections of the molecular packing of compound **3** [Boc-Aze(Alloc)-Ala-NHMe], with the molecules colored and displaying only polar hydrogens for clarity. On the left-hand side, a helical arrangement composed by 4 dimers (A1-B1, A2-B2, A3-B3 and A4-B4) is shown. On the right-hand side, the packing of 4 helical arrangements (colored in cyan, yellow, orange and pink respectively) is shown. Image generated with PyMOL.[S13]

Table S3. Crystal data and structure refinement for compounds **2** and **3.**

Compound	2	3
Empirical formula	$C_{20}H_{34}N_4O_6$	$C_{42}H_{72}N_8O_{12}$
Formula weight	427.958	881.07
Temperature (K)	100	120
Crystal system	monoclinic	tetragonal
Space group	$P2_1$	$P4_1$
a, b, c (Å)	24.457(13), 8.811(19), 27.27(2)	19.726(1), 19.726(1), 11.851(1)
α, β, γ (°)	90, 93.07(6), 90	90, 90, 90
Volume/Å^3	5869(14)	4611.4(5)
Z	10	4
ρ_{calc}g/cm^3	1.211	1.269
μ/mm^{-1}	0.129	0.768
F(000)	2310.3	1904.0
Radiation	synchrotron (λ = 0.82654)	CuKα (λ = 1.54184)
2Θ range for data collection (°)	1.74 to 53.38	8.704 to 108.282
Index ranges	-27 ≤ h ≤ 30, -11 ≤ k ≤ 11, -30 ≤ l ≤ 34	-19 ≤ h ≤ 19, -13 ≤ k ≤ 12, -11 ≤ l ≤ 6
Reflections collected	23084	2964
Independent reflections	12145 [R_{int} = 0.0523, R_{sigma} = 0.1137]	2964 [R_{int} = ?, R_{sigma} = 0.0806]
Data/restraints/parameters	12145/1906/1511	2964/10/571
Goodness-of-fit on F^2	1.716	0.989
Final R indexes [I>=2σ (I)]	R_1 = 0.1538, wR_2 = 0.4022	R_1 = 0.0573, wR_2 = 0.1259
Final R indexes [all data]	R_1 = 0.2369, wR_2 = 0.5007	R_1 = 0.0770, wR_2 = 0.1342
Largest diff. peak/hole / e Å^{-3}	0.79/-0.44	0.21/-0.15

6. References.

[S1] D. A. Case, T. A. Darden, T. E. Cheatham, III, C. L. Simmerling, J. Wang, R. E. Duke, R. Luo, M. Crowley, R. C. Walker, W. Zhang, K. M. Merz, B. Wang, S. Hayik, A. Roitberg, G. Seabra, I. Kolossvary, K. F. Wong, F. Paesani, J. Vanicek, X. Wu, S.R. Brozell, T. Steinbrecher, H. Gohlke, L. Yang, C. Tan, J. Mongan, V. Hornak, G. Cui, D. H. Mathews, M. G. Seetin, C. Sagui, V. Babin, P. A. Kollman (2008), AMBER 10, University of California, San Francisco.

[S2] Y. Cao, T.-X. Xiang, B. D. Anderson. *Mol. Pharmaceutics* **2008**, *5*, 371.

[S3] D. M. Shendage, R. Fröhlich, G. Haufe. *Org. Lett.* **2004**, *6*, 3675.

[S4] J. Y. Choi, R. Fuerst, A. M. Knapinska, A. B. Taylor, L. Smith, X. Cao, P. J. Hart, G. B. Fields, W. R. Roush. *J. Med. Chem.* **2017**, *60*, 5816.

[S5] C. Rabong, U. Jordis, J. B. Phopase. *J. Org. Chem.* **2010**, *75*, 2492.

[S6] A. Volonterio, S. Bellosta, P. Bravo, M. Canavesi, E. Corradi, S. V. Meille, M. Monetti, N. Moussier, M. Zanda. *Eur. J. Org. Chem.* **2002**, 4282.

[S7] A. H. Van Oijen, C. Erkelens, J. H. Van Boom, R. M. J. Liskamp. *J. Am. Chem. Soc.* **1989**, *111*, 9103.

[S8] M. Lista, J. Areephong, N. Sakai, S. Matile. *J. Am. Chem. Soc.* **2011**, *133*, 15228.

[S9] D. Núñez-Villanueva, L. Infantes, M. T. García-López, R. González-Muñiz, M. Martín-Martínez. *J. Org. Chem.* **2012**, *77*, 9833.

[S10] H. Kessler. *Angew. Chem. Int. Ed.* **1982**, *21*, 512.

[S11] L. Belvisi, C. Gennari, A. Mielgo, D. Potenza, C. Scolastico. *Eur. J. Org. Chem.* **1999**, *1999*, 389.

[S12] C. R. Groom, I. J. Bruno, M. P. Lightfoot, S. C. Ward. *Acta Crystallogr. B Struct. Sci. Cryst. Eng. Mater.* **2016**, *72*, 171.

[S13] The PyMOL Molecular Graphics System (open-source PyMOL). Version 2.3.0a0. Schrödinger, LLC.
